# Purification and Inhibitor Screening of the Full-Length SARS-CoV-2 Nucleocapsid Protein

**DOI:** 10.3390/molecules30132679

**Published:** 2025-06-20

**Authors:** Chen Chen, Zhengfu Zhang, Qiao Zheng, Yingshun Zhou, Shujun Zhang

**Affiliations:** Department of Biochemistry and Molecular Biology, School of Basic Medical Sciences, Southwest Medical University, Luzhou 646000, China; 20220199120044@stu.swmu.edu.cn (C.C.); 20200140330135@stu.swmu.edu.cn (Z.Z.); 20220140330107@stu.swmu.edu.cn (Q.Z.)

**Keywords:** SARS-CoV-2, COVID-19, nucleocapsid protein, expression and purification, virtual screening, inhibitor

## Abstract

Severe acute respiratory syndrome coronavirus 2 has undergone several mutations since 2020, and novel variants continue to emerge to this day. The immune escape ability of the emerging mutants is enhanced and results in robust transmissibility. The neutralizing ability of the antibodies produced in the human body during previous infections is decreased against some of these mutants, which poses a severe challenge to the preventive and therapeutic effectiveness of vaccines and antibody drugs. The nucleocapsid protein is one of the main structural proteins of the coronavirus and plays an important role in the life cycle of the novel coronavirus. This protein is one of the key targets for drug development, and the first major step in drug development is to obtain pure nucleocapsid proteins. However, since nucleocapsid proteins have a nucleic acid-binding function and automatically undergo liquid–liquid phase separation and agglomeration, the purification of full-length nucleocapsids is challenging. In this context, a set of easy-to-operate processes was developed in this study for the purification of nucleocapsid proteins. Finally, a pure full-length nucleocapsid protein without nucleic acid contamination was obtained, which exhibited significantly enhanced accessibility for structural and functional virological studies, vaccine development, and related research applications. Further, the nucleic acid-binding domain of the nucleocapsid protein was targeted, and potential severe acute respiratory syndrome coronavirus 2 inhibitors were identified using virtual screening and biolayer interferometry technology. Notably, the eukaryotically expressed nucleocapsid protein demonstrated a significantly greater binding affinity for Light Green SF Yellowish (K_D_ = 119.7 nM) compared to that demonstrated by its prokaryotic counterpart (K_D_ = 19.9 × 10^3^ nM). The findings of this study suggest the importance of considering both protein source and post-translational modifications of the target proteins to be used in drug screening workflows. Therefore, this compound not only represents a novel therapeutic candidate for COVID-19 but also a critical tool for elucidating antiviral mechanisms.

## 1. Introduction

The coronavirus disease 2019 (COVID-19) pandemic had various implications at the medical, social, political, and financial levels [1,2,3,4]. Although the introduction of genetically engineered vaccines, mRNA vaccines, live adenovirus vector vaccines, and inactivated vaccines effectively controlled the spread of severe acute respiratory syndrome coronavirus 2 (SARS-CoV-2) and considerably reduced the severe morbidity and mortality associated with COVID-19, numerous mutations in SARS-CoV-2 strains emerged toward the end of 2020 [5,6,7,8]. Owing to the increase in the immune escape ability of these emerging mutants, the proportion of mutants among the global epidemic strains rapidly increased, demonstrating a stronger transmission advantage [9,10]. The neutralizing ability of the antibodies produced in the human body during previous infections was decreased against some of these mutants, which raised concerns regarding “viral immune escape” [11,12]. Therefore, it is important to closely monitor future mutations in this virus and other types of transmission alongside the development of universal and novel coronavirus vaccines and drugs.

SARS-CoV-2 is the seventh coronavirus that has caused infection in humans, after the low pathogenic members HCoV-OC43, HCoV-HKU1, HCoV-NL63, and HCoV-229E, as well as the highly pathogenic SARS-CoV and MERS-CoV [13]. The SARS-CoV-2 genome consists of about 30,000 bases containing two large overlapping open reading frames (ORF1a and ORF1b) and encodes four structural proteins, namely, spike, envelope, membrane, and nucleocapsid (N) proteins, along with nine contactable proteins [14]. ORF1a and ORF1b undergo further processing to produce 16 non-structural proteins (Nsp1–16) [13]. Among these virus proteins, the N protein is the core component of the virus [15]. It is a heterostructured, 419 amino acid-long, multidomain RNA-binding protein. Similarly to the other novel coronaviruses, the SARS-CoV-2 N protein has two conserved, independently folded domains, the N-terminal domain (NTD) and the C-terminal domain (CTD), connected by an inherently disordered region (IDR) referred to as the central linking region (LKR) [16]. LKR includes a Ser/Arg (SR)-rich region that contains putative phosphorylation sites. In addition, two IDRs are present on both sides of the NTD and CTD of the coronavirus and are known as the N-arm and C-tail, respectively [16,17]. The NTD, CTD, and IDR are responsible for RNA binding, RNA binding and dimerization, and regulation of the RNA binding activity and oligomerization of NTD and CTD, respectively [15,18].

Multiple structural and non-structural proteins of SARS-CoV-2 have been extensively investigated as therapeutic targets in the development of antiviral drugs [19,20,21]. Notably, the N protein is the most abundant protein in the virion and is the decisive factor in its virulence and pathogenesis [22]. The N protein binds to the viral genomic RNA and packages the RNA into a ribonucleoprotein complex [14,15,22]. In addition to their ability to assemble, N proteins have other functions, including the transcription and replication of viral mRNA and immune regulation [23]. The N protein of SARS-CoV-2, in particular, has been found to counteract the host RNAi-mediated antiviral response through its double-stranded RNA binding activity, which enables it to act as a viral inhibitor for RNA silencing, thereby making it suitable as a key target for viral diagnosis and the development of vaccines and drugs [13,22,23,24]. Royster et al. demonstrated that the novel compound K31 could bind to the SARS-CoV-2 N protein and competitively inhibit its interaction with the 3′-terminal region of the viral genomic RNA, thereby suppressing SARS-CoV-2 replication in Caco2 cells [25]. Kumari et al. established a high-throughput screening (HTS) method to identify high-affinity ligands that target the NTD of the N protein. Three inhibitors—ceftazidime, sennoside A, and tannic acid—were identified using this method. All three inhibitors disrupted the interaction between the N protein and RNA probes. Notably, ceftazidime and sennoside A exhibited nanomolar-range binding affinities for the N protein [26]. Using network pharmacology and big data analysis, Chen et al. determined and validated that folic acid targets the N protein to suppress host RNA interference (RNAi) [27]. The interaction between the SARS-CoV-2 N protein and genomic RNA is initiated at specific RNA regions, which subsequently induce the formation of consecutive polymeric viral structures with characteristic structural motifs. Kuge et al. discovered one such functional RNA element, the guanine 12-mer (G12, Gggggggggggg) [28]. The presence of G12 either inhibits the formation of small N protein assemblies or significantly alters phase separation, resulting in large droplets with indistinct phase boundaries, which is indicative of G12 acting as a potent inhibitor of RNA–N protein interactions [28]. In addition to the development of direct inhibitors targeting the N protein, viral replication can also be suppressed through the inhibition of key enzymatic components in the viral replication cycle. A notable example was demonstrated by Han et al. in their investigation of papain-like protease (PLpro), a critical enzyme for SARS-CoV-2 replication [29]. These authors identified a chloroacetamide fragment designated Compound **1**, which inhibited SARS-CoV-2 replication in cellular models by forming covalent bonds with the catalytic cysteine residue in the active site of PLpro. This compound exhibited low non-specific reactivity toward thiol groups and is, therefore, considered a highly promising candidate for SARS-CoV-2 inhibition.

However, the basic function of N proteins is to bind to the genomic RNA and form protective nucleocapsids in mature viruses [14,30,31]. The inherent ability of the N protein to interact with nucleic acids renders it difficult to purify [30,32]. Moreover, N proteins can undergo liquid–liquid phase separation (LLPS) and thereby promote the formation of dense liquid condensates, increasing the difficulty of purification [15,33,34,35]. Notably, significant differences in the structure and phase separation properties have been observed between nucleic acid-contaminated and uncontaminated N proteins [32]. Nucleic acid contamination can seriously affect the molecular properties of purified N protein, thereby hampering the results of subsequent research and development of vaccines and drugs prepared using these N proteins. Currently, the integration and optimization of diverse protein purification techniques has enabled the development of nucleic acid-free, full-length N protein with high purity [32,36,37,38,39]. Building upon this foundation, in this study, a further streamlined purification methodology to significantly enhance operational efficiency while maintaining structural integrity was developed. Polyethyleneimine (PEI) is a linear polymer, with (-CH_2_CH_2_-NH-)*n* as the repeating units [40]. The *n* value is generally 700–2000, and the overall molecular weight of this polymer is 30–90 kDa. The presence of numerous repeated imine units leads to a high positive charge of PEI in near-neutral solutions; thus, PEI can adsorb negatively charged biomolecules, such as nucleic acids or acidic proteins, resulting in complexes, and also initiate flocculation, resulting in the coprecipitation of PEI and bound biomolecules [40,41,42,43]. In this study, the above phenomenon was exploited, and PEI binding to nucleic acids was utilized to remove nucleic acids from the N protein. Ammonium sulfate precipitation, dialysis, and nickel column purification were then performed to obtain the uncontaminated N protein. Thus, a simple process for the purification of the full-length N protein without nucleic acid contamination was developed. Moreover, using virtual screening, the potential inhibitors for nucleocapsid proteins were identified.

## 2. Results

### 2.1. N Protein Purification Process

**Figure 1:**
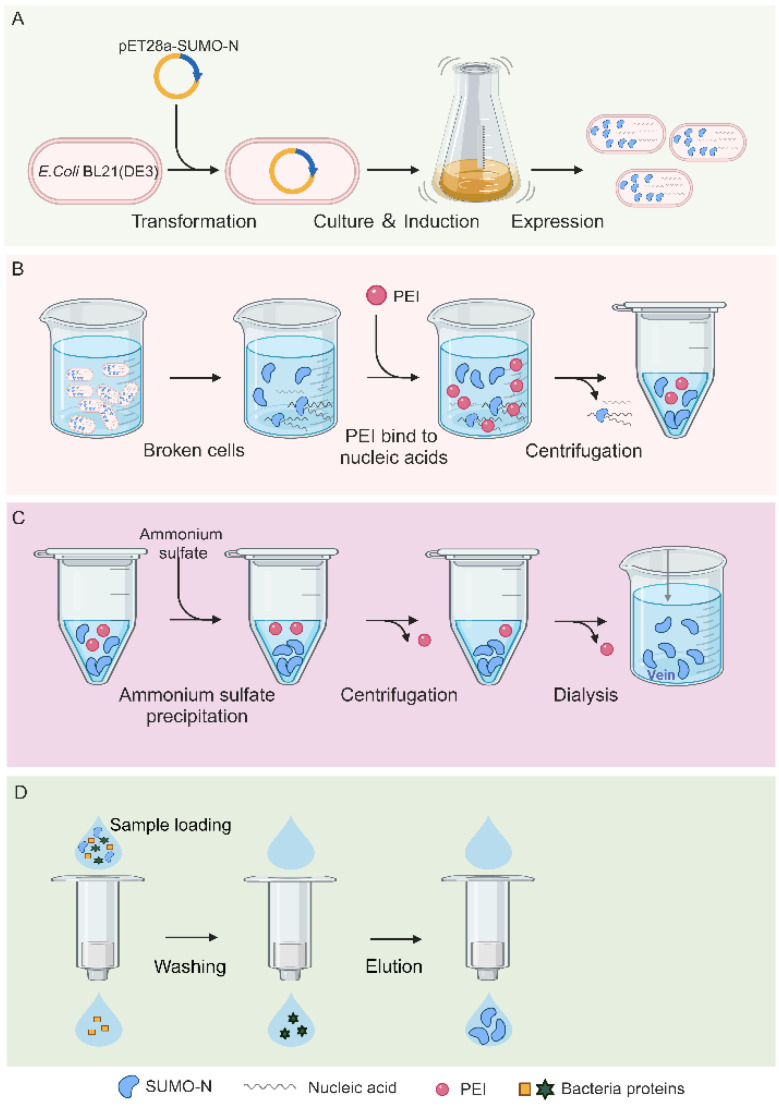
The expression and purification process of the full-length N protein. (**A**) The process of prokaryotic expression of the N protein. (**B**) PEI precipitates nucleic acid. (**C**) The enrichment of the N protein and removal of free PEI using ammonium sulfate precipitation. (**D**) N protein purification using a nickel column.

In order to obtain the N protein without nucleic acid contamination, the following purification process was used (Figure 1): first, the soluble N protein was expressed in *E. coli* (Figure 1A), following which the nucleic acid in the N protein was removed by exploiting the characteristic PEI binding to nucleic acid (Figure 1B). The N protein was subsequently precipitated with ammonium sulfate, and the free and unbound PEI was retained in the supernatant (Figure 1C). The N protein was enriched using centrifugation, and the free PEI was removed. The dialysis step was performed to completely remove the residual PEI and ammonium sulfate. Finally, the purified N protein was obtained using nickel column purification (Figure 1D). The experimental results of each step are described in detail below.

### 2.2. Prokaryotic Expression System

Compared to the eukaryotic expression system, the prokaryotic expression system offers the advantages of high efficiency and low cost when performing protein expression. *E. coli* is a good choice for expressing the N protein. Soluble N protein was previously reported to be expressed under mild induction conditions, although with low expression levels [39]. In a study, solubilizing the N protein by fusing with SUMO and then expressing considerably increased the expression of soluble N protein [32]. Therefore, in this study, the N protein was fused with SUMO for expression.

### 2.3. Ultrasonic Fragmentation

**Figure 2:**
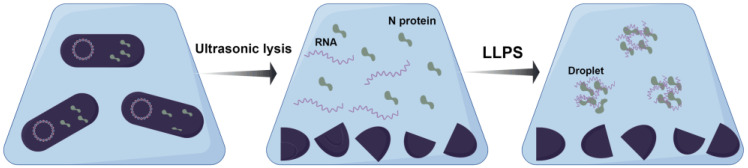
A schematic illustration of droplet formation using the N protein and RNA. In *E. coli*, the N protein and RNA remain spatially segregated until cell disruption releases the two into the solution (**left**). Upon mixing (**middle**), these components spontaneously undergo LLPS, forming dynamic liquid droplets that coalesce into larger structures upon contact (**right**).

First, the collected bacteria were resuspended in conventional buffer B, and it was observed that the partitioned liquid was turbid. Even when these aggregates were removed through high-speed centrifugation, flocs slowly appeared in the supernatant, causing the solution to become sticky. These findings, in conjunction with previous experimental evidence, strongly suggested the occurrence of the LLPS phenomenon due to the release of bacterial nucleic acid because of the fragmentation and binding of the N protein to nucleic acid. Under low salt concentrations, the N protein can bind to nucleic acids, and the LLPS phenomenon occurs automatically [33,35]. The positively charged N protein has an electrostatic effect on negatively charged RNA/DNA, which drives the formation of condensates (Figure 2) [33]. When buffer A was used with a high salt concentration (1 M NaCl) to partition the suspension, the partitioned solution was clarified, and no obvious LLPS phenomenon occurred. Adding solid NaCl directly to the partitioned supernatant in which the LLPS phenomenon had occurred could reverse the LLPS phenomenon. These results indicated that increasing the salt concentration can prevent or weaken the formation of LLPS. Finally, the buffer containing 1 M NaCl was selected for ultrasonic fragmentation experiments.

### 2.4. PEI Precipitation of Nucleic Acids

**Figure 3:**
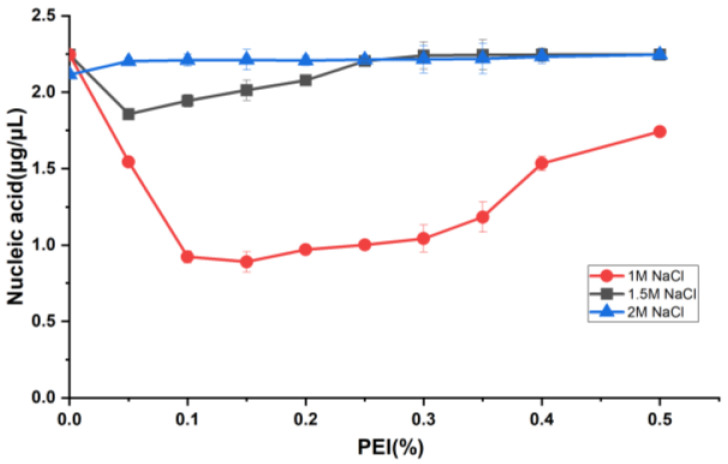
PEI precipitates nucleic acid.

**Figure 4:**
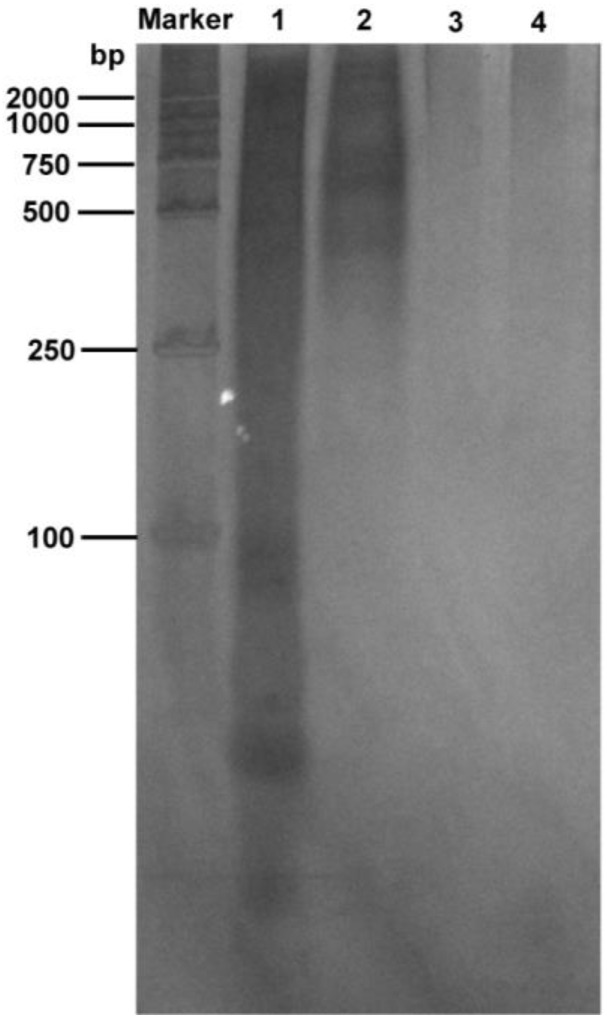
Silver staining results. M, marker; lane 1, the nucleic acid contained in the fragmented supernatant; lane 2, the residual nucleic acid contained in the fragmented supernatant after PEI treatment; lane 3, the residual nucleic acid in the sample after PEI, ammonium sulfate precipitation, and nickel column purification; lane 4, the control (buffer containing 50 mM Tris-HCl, 1 M NaCl, pH 8.0).

PEI is a linear polymer rich in positive charge and can form charge-neutralizing precipitates with many negatively charged nucleic acid molecules, allowing for the removal of nucleic acids [40]. Therefore, in this study, the nucleic acid in the N protein sample was removed using the PEI precipitation method. In order to study the effect of nucleic acid precipitation using different PEI concentrations under different salt concentrations, the NaCl concentration gradient of 1, 1.5, and 2 M was selected. Since the full-length N protein demonstrated the LLPS phenomenon during the initial cell fragmentation step at low salt concentrations, PEI binding to nucleic acid at low salt concentrations was not performed. The PEI concentration of each sample under three NaCl concentrations was varied from 0.05% to 0.5% in increments of 0.05%. The nucleic acid content in the supernatants of each sample after PEI precipitation was determined. As shown in Figure 3, with 1 M NaCl, the nucleic acid residue in the sample gradually decreased with increasing PEI concentration. The final concentration of 0.15% PEI resulted in the best nucleic acid precipitation effect, as the nucleic acid residue in the sample was the lowest. Further, the silver staining experiments revealed that PEI removed most of the nucleic acids from the samples (Figure 4, lane 2). Thereafter, an increase in the PEI concentration significantly decreased nucleic acid precipitation. However, at 1.5 and 2 M NaCl, there was no visible effect of PEI on nucleic acid precipitation (Figure 3), indicating that the effect of PEI on nucleic acid precipitation decreased with increasing salt concentration. This was primarily attributed to the interaction between PEI and negatively charged molecules being affected by pH and salt concentration. An increase in salt concentration weakened the binding between PEI and the interacting molecules. Therefore, 0.15% PEI under 1 M NaCl was selected as the best condition for removing most of the nucleic acids from the protein sample. The residual nucleic acid was removed in the subsequent purification step.

### 2.5. Results of Ammonium Sulfate Precipitation

**Figure 5:**
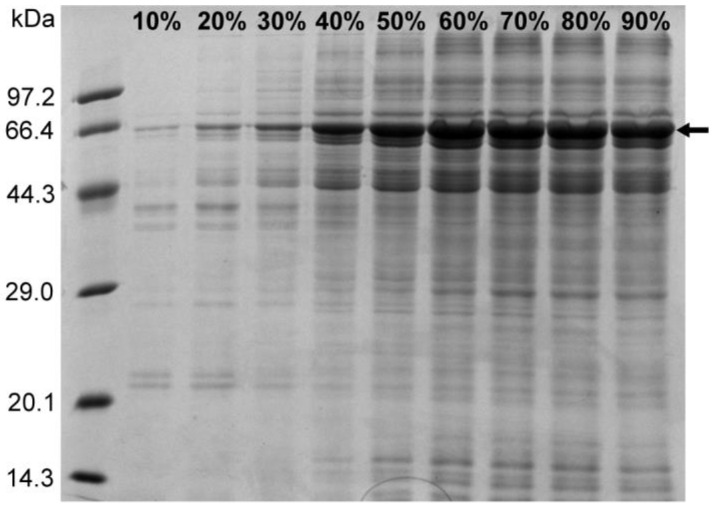
SDS-PAGE results for the ammonium sulfate-precipitated protein (the arrow indicates the N protein).

**Figure 6:**
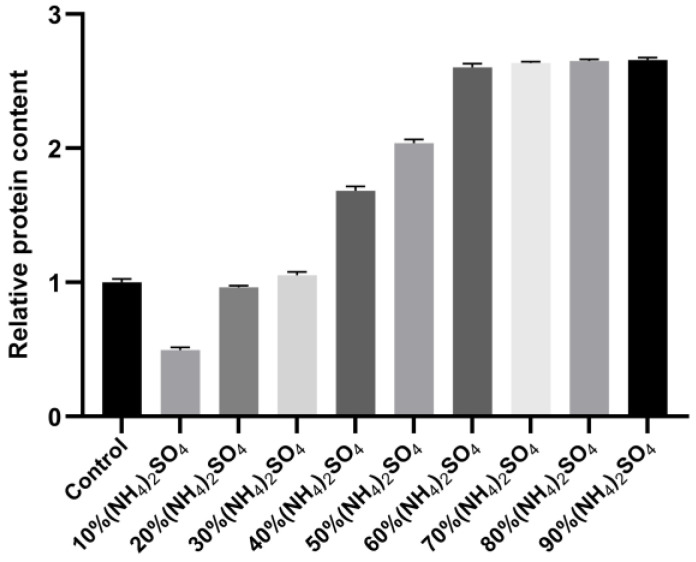
Normalization analysis of N protein treated with different ammonium sulfate concentrations. Color coding is for visual clarity only without semantic meaning.

The ammonium sulfate precipitation method enriches the sample protein and also exerts a certain purification effect by reducing the nucleic acid content in the precipitated protein. In addition, certain free PEI molecules existing in the sample treated with PEI can interfere with the subsequent purification step using the nickel column, and the ammonium sulfate precipitation step removes these free PEI molecules. In order to study the relationship between the concentration of ammonium sulfate and the precipitation effect of the target protein, saturated ammonium sulfate solution was added to the sample containing 1 M NaCl after PEI treatment, in amounts that resulted in ammonium sulfate concentrations varying from 10% to 90% in increments of 10%. The sample was subsequently centrifuged at 4 °C for 2 h, after which the protein precipitate was resuspended in buffer C (50 mM Tris-HCl, 1 M NaCl, pH 8.0), followed by an SDS-PAGE analysis. The results revealed that the amount of N protein precipitated using ammonium sulfate increased with increasing ammonium sulfate concentration at a NaCl concentration of 1 M (Figure 5). At 60% ammonium sulfate concentration, the precipitation of N protein did not increase significantly, as no visible difference was observed on the gel map (Figure 6). Therefore, 60% ammonium sulfate was selected for enriching the target protein while removing the residual nucleic acid and PEI.

### 2.6. Results of N Protein Purification by Nickel Column

**Figure 7:**
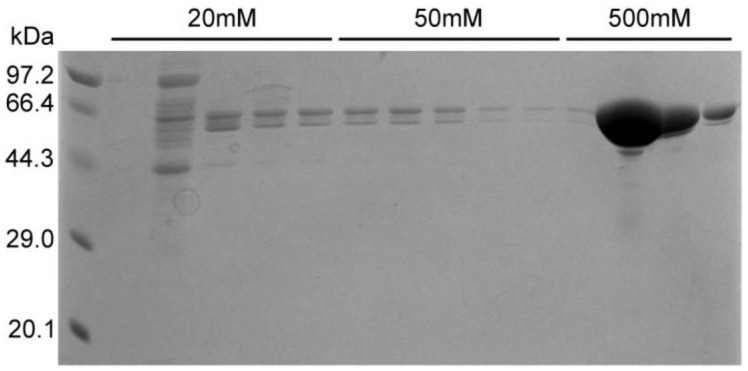
SDS-PAGE results for N protein.

After treatment with ammonium sulfate, traces of free PEI may remain; therefore, the sample in this study was dialyzed and then passed through a nickel column to obtain pure N protein. After washing with 20 mM and 50 mM imidazole, pure N protein was obtained at a concentration of 500 mM imidazole (Figure 7). The purified samples were also subjected to silver staining. Pure N protein without nucleic acid contamination was obtained after performing the abovementioned purification steps (Figure 4, lane 3). In addition, the N protein was purified directly using a nickel column after crushing and centrifugation in buffer C (without PEI or ammonium sulfate treatment). Although the N protein was also obtained, the N protein precipitated in the subsequent dialysis step because the obtained N protein contained nucleic acid (Figure S1A). The N protein treated with PEI and ammonium sulfate did not precipitate (Figure S1B).

### 2.7. Screening and Identification of Inhibitors Targeting N Protein

**Figure 8:**
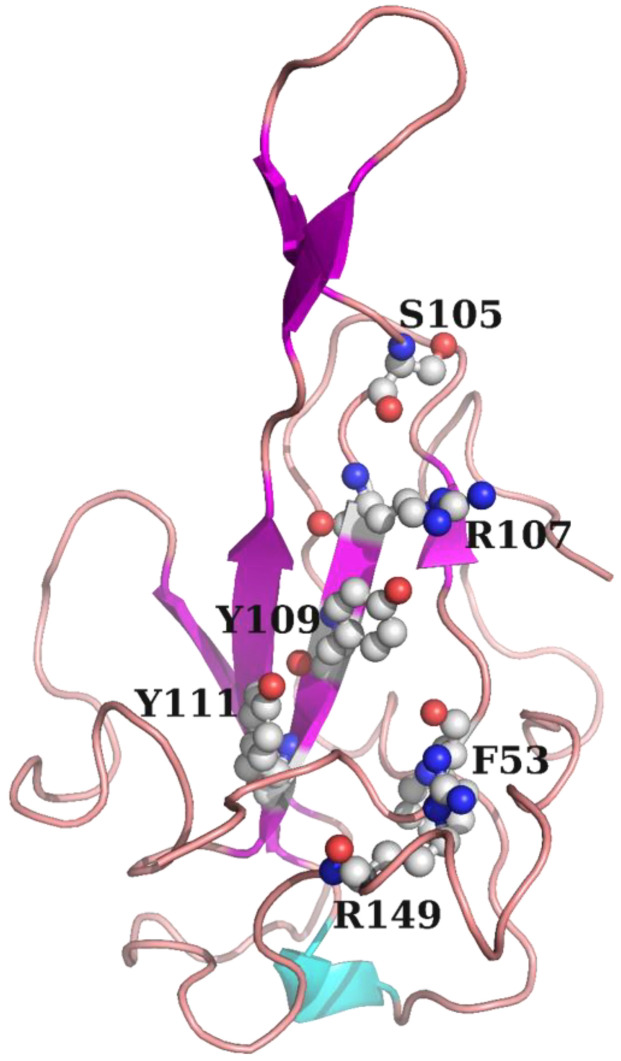
Critical residues in the N protein NTD. The site of the white spheres was selected as the binding pocket.

The N protein of SARS-CoV-2 is crucial for its diagnosis, vaccine production, and drug development [13,15,19]. The present study was mainly aimed at the development of novel coronavirus drugs. Therefore, using the NTD of the N protein of SARS-CoV-2 as the receptor protein, 6000 natural products and their derivatives, as well as 2800 approved drug molecules, were screened using Molecular Operating Environment (MOE) software (v.2022.02; Chemical Computing Group) to identify SARS-CoV-2 inhibitors [44]. The molecular docking pocket of the N protein is shown in Figure 8. The structure and docking score of the top 20 natural products and their derivatives are presented in Figure S2. The top 20 approved drug molecules are shown in Figure S3. The affinity between these potential small-molecule inhibitors and N proteins was determined using the biolayer interferometry (BLI) technique.

### 2.8. Analysis of the Interaction Between the N Protein and the Small-Molecule Inhibitor Light Green SF Yellowish (LGSFY)

**Figure 9:**
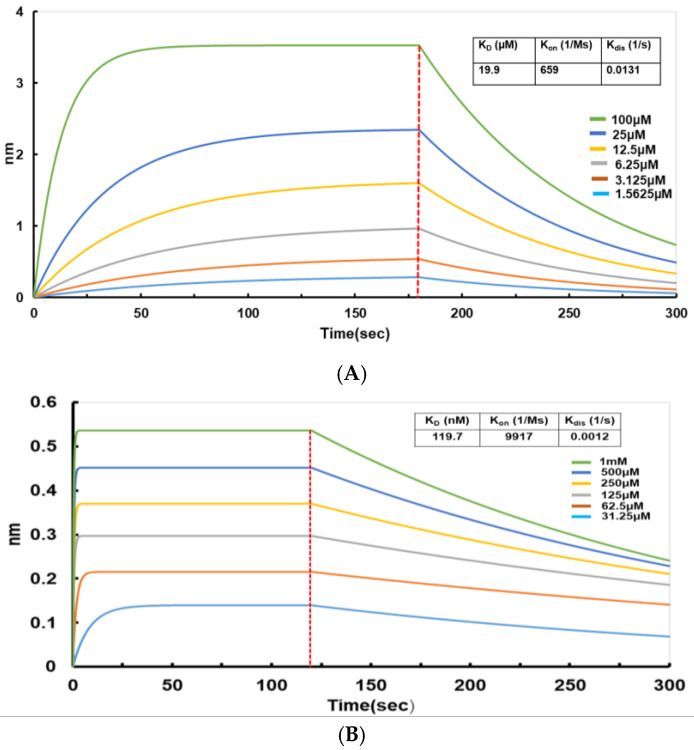
Real-time kinetic binding sensorgrams for different concentrations of Light Green SF Yellow to the N protein. (**A**) Real-time kinetic binding sensorgrams for the purified N protein expressed in a prokaryote to Light Green SF Yellowish. (**B**) Real-time kinetic binding sensorgrams for the purified N protein expressed in a eukaryote to Light Green SF Yellowish.

BLI is an unmarked, real-time optical detection technology that is primarily used for the omnidirectional quantitative analysis of biomolecule interactions. BLI allows for monitoring the entire intermolecular binding process in real-time and calculating crucial data, such as intermolecular affinity (K_D_), binding rate (K_on_), and dissociation rate (K_dis_). Several studies have successfully demonstrated the interactions between notable proteins and small molecules using BLI technology [45,46,47]. Therefore, in this study, BLI technology was used to determine the affinity between small molecules and the full-length N protein expressed in a prokaryote and then purified. Among the screened small molecules, Light Green SF Yellowish exhibited a good affinity for the N protein. The binding affinity K_D_ was 19.9 × 10^3^ nM (Table 1), suggesting that this protein is potentially a good inhibitor of the N protein (Figure 9A). The proteins produced in *E. coli* lack the post-translational modifications typically found in mammalian cells, such as complex phosphorylation and glycosylation modifications, and such deficiencies may directly alter the binding interfaces and thereby influence the screening outcomes of small-molecule inhibitors. Consequently, binding affinity assays between LGSFY and the N protein purified from human 293 cells (HEK293) were also performed (Figure 9B). Surprisingly, the binding affinity was determined to be 119.7 nM (Table 1), which is about an order of magnitude greater than that observed using prokaryotic expression of N protein (K_D_ = 19.9 × 10^3^ nM). This disparity in affinity could be attributed to the eukaryotic-specific modifications to the N protein, which may have altered the protein’s binding interface with LGSFY and potentially enhanced the interaction.

### 2.9. Virtual Screening and Molecular Modeling

**Figure 10:**
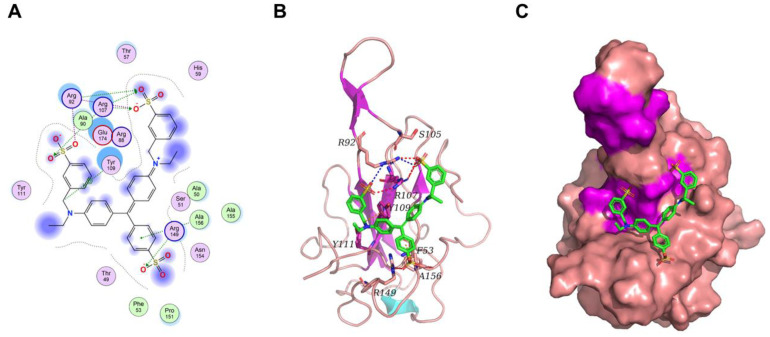
The binding mode of Light Green SF Yellowish and the N protein. (**A**) The 2D binding mode of Light Green SF Yellowish and the N protein. (**B**) The 3D binding mode of Light Green SF Yellowish and the N protein. (**C**) The surface binding mode of Light Green SF Yellowish and the N protein. The Light Green SF Yellowish is depicted in green. The backbone, surface, and residue of the N protein are depicted in pink (turns), magenta (β-sheets), and cyan (α-helices), respectively. The hydrogen bonds are depicted using red dashed lines. The blue dashes represent salt bridges. The orange dashes represent H-π conjugates.

In order to elucidate the differential binding affinities of Light Green SF Yellowish to the N protein obtained using distinct expression systems, bioinformatic analyses were performed. The binding between LGSFY and the N protein was studied using molecular docking. The binding mode between LGSFY and the N protein is depicted in Figure 10. Within the binding pocket, two oxygen atoms on the sulfate radical of LGSFY formed salt bridges with the two nitrogen atoms of Arg92, and four oxygen atoms on the three sulfate radicals of LGSFY, regarded as hydrogen bond acceptors, formed three hydrogen bonds with the nitrogen atoms of Arg92, Arg107, and Arg149. The carbon atom of LGSFY formed an H–π conjugate with the benzene ring of Tyr109, and the benzene ring of LGSFY formed an H–π conjugate with the nitrogen atom of Ala156.

**Figure 11:**
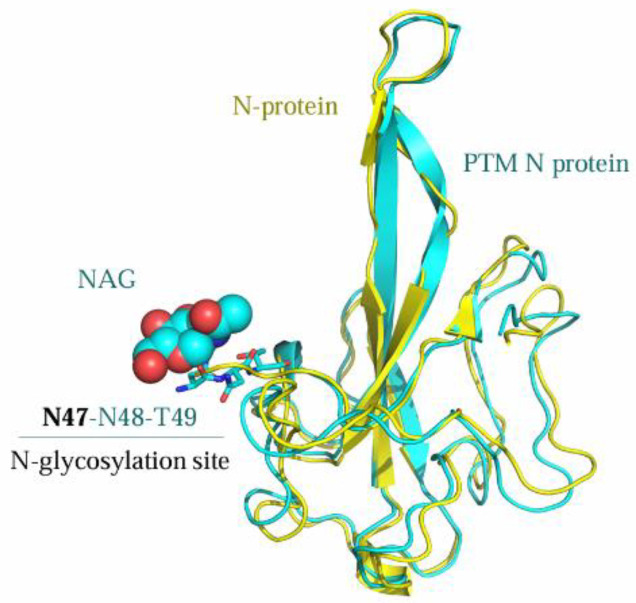
The sequence and structural alignment of the N-glycosylated N protein (cyan) and the unmodified N protein (yellow).

**Figure 12:**
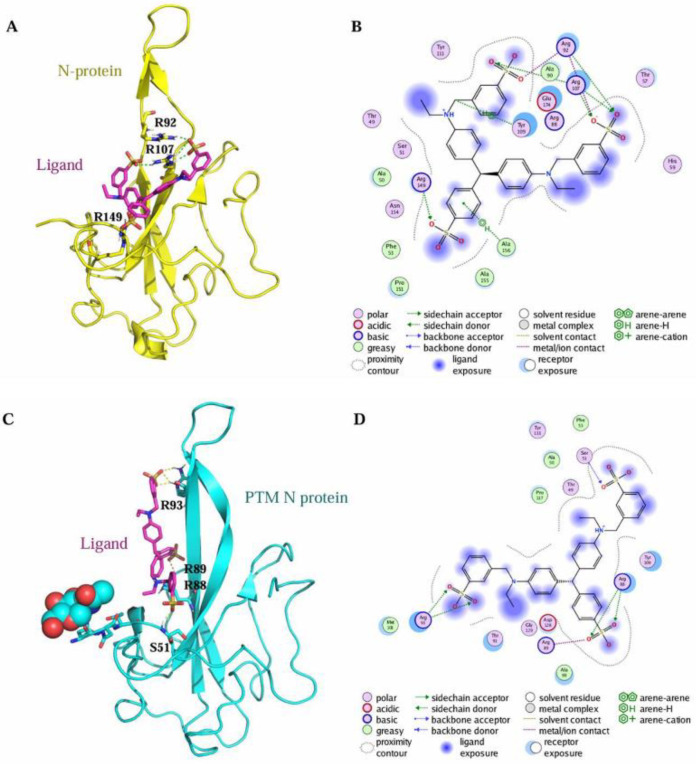
The characterization of the interaction between the N protein and the ligand Light Green SF Yellowish. (**A**) The docking pose of the ligand within the unmodified N protein (yellow). (**B**) The 2D interactions between the ligand and the unmodified N protein. (**C**) The docking pose of the ligand within the N-glycosylated N protein (cyan). (**D**) The 2D interactions between the ligand and the N-glycosylated N protein.

In order to further understand the structural changes of the N protein after post-translational modifications (PTMs), an N-acetylglucosamine (NAG) moiety was introduced at the N47 glycosylation site of the N protein (PDB ID: 7CDZ), which constitutes the amino acid sequence N47-N48-T49 satisfying the consensus motif (-NXS/T- with X ≠ P) for N-glycosylation. Supekar et al. have confirmed through an MS/MS analysis that 53% of N-glycosylation of the N protein commercially produced with a signal peptide (SP) occurs at N47 [24]. The structural refinement of the N47-NAG-N protein was performed using MOE with the AMBER99 force field, followed by sequence and structural alignment to compare the spatial structural differences between the modified and unmodified N proteins (Figure 11). Additionally, molecular docking studies were conducted to determine the binding affinities of the ligand Light Green SF Yellowish with both forms. Under the default docking function of MOE (GBVI/WSA dG, Amber10:EHT force field), the calculated docking scores of the ligands within the N47-NAG-N protein pocket and the unmodified N protein were −7.60 and −6.57 kcal/mol, respectively. A detailed binding mode analysis is presented in Figure 12A–D. Based on the docking results, the ligand might exhibit stronger binding affinity with the N-glycosylated N protein than with the unmodified form. However, no direct interaction between the ligand and the N47-NAG moiety is observed in Figure 11. Given that glycosylation frequently modulates protein–ligand binding via allosteric effects [48,49], we propose that post-translational modifications (e.g., glycosylation) may facilitate the association between the N protein and the ligand through conformational remodeling.

## 3. Materials and Methods

### 3.1. Prokaryotic Expression of the Nucleocapsid Protein

The N protein expression vector (GenBank: UBE86422.1) was constructed using gene synthesis. In order to promote the soluble expression of the N protein, a small ubiquitin-like modifier (SUMO) was fused at the amino terminus of the N protein. Then, to express the SUMO and N proteins in the pET28a plasmid vector, the complementary DNAs of both proteins and the pET28a vector were digested using the upstream and downstream restriction enzymes NcoI and XhoI, respectively. This was followed by ligation, which resulted in the recombinant pET28a-SUMO-N expression vector. The pET28a-SUMO-N expression vector was then transformed into *E. coli* competent BL21 (DE3) cells and the transformants were selected through overnight incubation in 5 mL of liquid Luria–Bertani (LB) medium supplemented with 50 μg/mL kanamycin at 37 °C inside a shaker incubator at 220 rpm. Subsequently, 1 mL of the culture was added to 1 L of LB medium containing 50 μg/mL kanamycin under continuous shaking at 220 rpm at 37 °C. When the OD600 was 1.0, IPTG (5 mM) was added to induce the expression of the N protein at 20 °C for 16 h at 220 rpm. The cells were harvested through centrifugation at 6000 rpm for 10 min.

### 3.2. Purification of Nucleocapsid Protein

#### 3.2.1. Ultrasonic Fragmentation of Bacteria

One portion of the collected bacteria was resuspended in buffer A (50 mM Tris-HCl, 1 M NaCl, 10% glycerol, DNase I, RNase A, 1 mM DTT, pH 8.0), to be used as the control, while the other portion was resuspended in buffer B (50 mM Tris-HCl, 0.3 M NaCl, 10% glycerol, DNase I, RNase A, and 1 mM DTT, pH 8.0) and used for ultrasonic fragmentation.

#### 3.2.2. PEI-Precipitated Nucleic Acid

In order to study the effects of nucleic acid precipitation using different PEI concentrations under different salt concentrations, buffer C (50 mM Tris-HCl, 1 M NaCl, pH 8.0), buffer D (50 mM Tris-HCl, 1.5 M NaCl, pH 8.0), and buffer E (50 mM Tris-HCl, 2 M NaCl, pH 8.0) were prepared and pooled to prepare a 5% PEI solution, with the final pH adjusted to 8.0. The bacterial samples obtained from the above-stated three solutions were resuspended. The NaCl concentration in the lytic solution was 1 M, 1.5 M, or 2 M. The supernatants from the three different cell lysis media, each featuring different NaCl concentrations, were subsequently divided evenly into an equivalent number of samples.

Each sample was introduced with 5% polyethyleneimine (PEI), yielding PEI concentrations corresponding to the NaCl concentrations, as follows: 0.05%, 0.1%, 0.15%, 0.2%, 0.25%, 0.3%, 0.4%, and 0.5%. Finally, the sample volumes were adjusted to match those of the buffer solution containing the corresponding NaCl concentration. The resulting mixtures were incubated at 4 °C for 30 min, followed by centrifugation at 15,000 rpm for 10 min. The subsequent determination of the nucleic acid content was conducted using the Nano100 instrument (Allsheng Allsheng Co., Ltd., Suzhou, China).

#### 3.2.3. Ammonium Sulfate Precipitation

A saturated ammonium sulfate solution was prepared using buffers C, D, and E. In a number of partitioned liquid samples, NaCl concentration was adjusted to 1, 1.5, and 2 M, followed by the addition of saturated ammonium sulfate solution with the corresponding NaCl concentration, resulting in the following ammonium sulfate concentration gradients in the samples containing different NaCl concentrations: 10%, 20%, 30%, 40%, 50%, 60%, 70%, 80%, and 90%. Finally, the sample volume was adjusted to the same volume by adding the buffer containing the corresponding NaCl concentration. The samples were incubated at 4 °C for 2 h, followed by centrifugation at 15,000 rpm for 30 min. The supernatant was discarded, the protein precipitates were resuspended in buffer (50 mM Tris-HCl, 1 M NaCl, pH 8.0), and SDS-PAGE was performed to analyze the effect of ammonium sulfate on the precipitation of N protein at different concentrations.

#### 3.2.4. The Purification of N Protein Using a Nickel Column

The samples without PEI and ammonium sulfate were used as controls and purified directly using a nickel column, whereas the samples treated with PEI and ammonium sulfate were dialyzed to remove PEI and ammonium sulfate. The bag size in dialysis was 35,000 D, and the dialysate composition was 50 mM Tris-HCl, 1 M NaCl, and 5 mM imidazole, pH 8.0. The protein sample was applied to the nickel column, and 20 mM and 50 mM imidazole buffer were used to remove the impure proteins. Finally, the N protein was eluted with buffer containing 50 mM Tris-HCl, 1 M NaCl, and 500 mM imidazole, pH 8.0. The purity of the protein was assessed using SDS-PAGE.

### 3.3. N Protein Expressed in Mammalian Cells

The N protein expressed in human 293 cells (HEK293) and then purified was purchased from ACROBiosystems (Newark, DE, USA. Accession # QHO62115.1).

### 3.4. Silver Staining Experiment

An appropriate amount of sample was collected from each of the above steps and reserved for the analysis of nucleic acid residues using the rapid nucleic acid silver staining kit purchased from Coolaber (Beijing, China). A 12% nucleic acid PAGE gel was prepared with the following ingredients: water (7.9 mL), 30% acrylamide (4 mL), 5× tris borate EDTA buffer (3 mL), 10% ammonium persulfate (0.11 mL), and tetramethylethylenediamine (0.01 mL). Each sample (9 μL) was mixed with 1 μL of 10× loading buffer, and nucleic acid PAGE was performed for 1 h at 120 V and 250 mA.

After PAGE, the PAGE gel was transferred to a glass Petri dish containing deionized water and rinsed 3 times for 2 min each. The PAGE gel was subsequently transferred to an appropriate volume of fixing liquid (10% ethanol and 1% nitric acid), such that the PAGE gel remained immersed during gentle shaking at 40–60 rpm for 10 min. Thereafter, the fixing solution was discarded, and the PAGE gel was quickly rinsed three times with deionized water, each time for 30 s. The PAGE gel was then transferred to an appropriate volume of dyeing solution (0.2% silver nitrate), such that the PAGE gel remained immersed during gentle shaking for 5 min at room temperature. The dyed gel was quickly rinsed three times with deionized water, each time for 30 s. Finally, the gel was transferred to an appropriate volume of chromogenic solution (3% Na_2_CO_3_, 60 μL of formaldehyde, and 10 mg of Na_2_SO_4_), and 600 mL of deionized water was added until the DNA marker or positive control band was clearly visible. The PAGE gel was then photographed using a gel imager (Bio-Rad Laboratories, Hercules, CA, USA).

### 3.5. SDS-PAGE

In all sample treatments, an appropriate amount of sample was retained for SDS-PAGE analysis. A 12% SDS-PAGE gel was run for 2 h at 120 V and 250 mA. After electrophoresis, the gel was stained with Coomassie Brilliant Blue, decolorized with glacial acetic acid and ethanol, and finally photographed and analyzed using the Bio-Rad imaging system.

### 3.6. Virtual Screening

The docking module in MOE was used for structure-based visual screening (SBVS). About 6000 natural products and their derivatives, along with 2800 approved drug molecules, were selected to form the visual screening library. All the compounds were prepared using the Wash module in MOE. The structure of the N protein NTD was selected as the receptor, and its PDB ID was 7CDZ. On the basis of the literature, the molecular binding site in the receptor was selected around residues F53, S105, R107, Y109, Y111, and R149 [13]. All the compounds were subsequently ranked based on flexible docking with the “induced fit” protocol. The force field of AMBER10:EHT and the implicit solvation model of the reaction field (R-field) were selected. The protonation state of the protein and the orientation of the hydrogens were optimized using the QuickPrep module at pH 7 and a temperature of 300 K. In flexible docking, initially, the docked poses were ranked based on London dG scoring; then, a force field refinement was applied to the top 10 poses, followed by a rescoring of GBVI/WSA dG, and the best-ranked pose was retained. After docking, the compounds were clustered structurally using the fingerprint cluster module in MOE. The best-ranked 1000 molecules were ultimately identified as potential hits.

### 3.7. BLI Analysis

BLI experiments were performed using Octet-RED96e (Sartorius AG, Göttingen, Germany). The experiments were performed in an assay buffer containing 50 mM PBS (pH 7.5), 1 M NaCl, and 0.2% (*v*/*v*) Tween. The samples were added to the wells of a 96-well plate containing 200 μL per well. In each experiment, anti-His tagged biosensors (HIS1K, Sartorius AG, Göttingen, Germany) were loaded with N protein ligands and then immersed in different concentrations of inhibitor analytes. The ligand concentration for loading was 1 mg/mL (His marker). All experiments were accompanied by reference measurements conducted by immersing unloaded HIS1K tips in the wells with the same analyte. The experiments with different analyte concentrations also included zero analyte reference. Octet software version 10 (Sartorius) was employed for data processing and fitting.

### 3.8. Molecular Modeling

Molecular modeling studies were carried out using MOE software. The protein structure data were downloaded from the RCSB Protein Data Bank (http://www.rcsb.org/; accessed on 24 December 2023. Post-translational modifications of the protein were added manually, followed by structural optimization under the AMBER10 force field (RMSD gradient = 0.1 kcal/mol). Next, binding mode prediction was performed using the DOCK protocol, and default values were used for all docking parameters.

## 4. Conclusions

Although the global pandemic has exhibited an overall declining trend and the pressure on global health systems has reduced, SARS-CoV-2 continues to mutate, resulting in localized outbreaks in some countries and regions. Consequently, the research and development of vaccines and therapeutic drugs against SARS-CoV-2 continue worldwide. The N protein is the most abundant protein in the virion [50,51] and a determinant of SARS-CoV-2 virulence and pathogenesis [52]. It is recognized as a highly immunogenic antigen and a potential target for developing vaccines and drugs against SARS-CoV-2 infection [53,54,55,56,57]. One of the critical steps in drug development using the N protein as a target is the acquisition of pure N protein.

While several papers describing the structure, function, and molecular characteristics of the N protein have been published to date, the N protein samples obtained using previously described purification methods may be heavily contaminated with nucleic acids derived from the recombinant expression host organism [58,59,60,61]. The N protein has a structure that binds to nucleic acids, rendering the complete elimination of nucleic acid impurities challenging. This study attempted to develop an effective purification method capable of removing all nucleic acids bound to the N protein. First, the prokaryotic expression system was utilized to achieve a high expression of soluble N protein. Cell fragmentation causes N proteins to aggregate due to the occurrence of the LLPS phenomenon at low salt concentrations. This was significantly prevented by increasing the salt concentration to 1 M NaCl in the proposed method. In addition, the occurrence of LLPS is closely related to the contents of nucleic acids and N proteins in the system. As the expression of N protein was high, it was necessary to adjust the suspension volume according to the bacterial weight. Second, the nucleic acid was precipitated by exploiting the nucleic acid-binding characteristics of PEI. The precipitation effect of nucleic acid was the greatest at 0.15% PEI with 1 M NaCl. At 1.5 M NaCl, the nucleic acid-binding ability of PEI significantly decreased, and with 2 M NaCl, PEI lost its nucleic acid-binding ability. The main reason for this observation was that the nucleic acid-binding ability of PEI is affected by pH and salt concentration. Therefore, increasing the salt concentration weakens the binding of PEI to nucleic acids. The samples were subsequently precipitated using ammonium sulfate. This step not only enriched the sample to facilitate subsequent purification but also removed free PEI, which could have otherwise interfered with the protein purification step. Finally, the full-length N protein was purified using a conventional nickel column. Therefore, full-length N protein without nucleic acid contamination was successfully obtained using a set of purification processes.

Owing to the difficulty in obtaining full-length nucleocapsid proteins without nucleic acid contamination, most researchers often express only a certain domain of the nucleocapsid protein when developing inhibitors against it, which may result in the reduced efficacy of these inhibitors due to structural differences between the partial and full-length nucleocapsid proteins targeted and because the interactions often occur among domains. Therefore, full-length nucleocapsid protein is more suitable as a drug development target. In this study, inhibitor drugs against the obtained full-length nucleocapsid protein were identified using virtual screening along with biological experiments. Ultimately, a potential inhibitor, Light Green SF Yellowish, was obtained. During the initial phase of SARS-CoV-2 replication, N proteins remain unbound to nucleic acids. In theory, the small-molecule compound Light Green SF Yellowish can specifically bind these free nucleocapsid proteins, inhibiting the formation of ribonucleoprotein (RNP) complexes and ultimately suppressing viral replication. Even when N proteins have already bound RNA to form RNPs, the interaction, which primarily relies on electrostatic and hydrophobic forces rather than covalent bonds, is theoretically dynamic and reversible. Notably, the affinity between unmodified SARS-CoV-2 N protein and RNA was about 230 μM [62], which can theoretically enable Light Green SF Yellowish to competitively displace RNA from binding to N proteins. However, in physiological environments, N proteins undergo diverse post-translational modifications. The impact of these modifications on the efficacy of Light-Green SF Yellowish remains uncertain. Therefore, to resolve the problems mentioned above, binding affinity assays between Light Green SF Yellowish and the N protein purified from human 293 cells (HEK293) were performed. Surprisingly, the binding affinity (K_D_ = 119.7 nM) was greater than that observed when using prokaryotic expression for obtaining the N protein (K_D_ = 19.9 × 10^3^ nM). Docking results suggest that, compared to the unmodified form, Light Green SF Yellowish may exhibit stronger binding affinity towards the N-glycosylated N protein (at position N47). This difference in affinity may arise from eukaryotic-specific modifications of the N protein and associated conformational changes. These alterations could potentially modify the binding interface with Light Green SF Yellowish and may enhance the interaction.

In summary, a streamlined purification method for obtaining full-length nucleocapsid proteins was developed. The method ensures high efficiency and quality. This advancement significantly enhances the accessibility of N proteins for structural and functional virology studies, vaccine development, and related research domains. In addition, Light Green SF Yellowish was identified as a potent small-molecule inhibitor. Notably, the enhanced binding affinity of Light Green SF Yellowish toward post-translationally modified N protein suggests the necessity of incorporating the protein source and post-translational modification status into drug screening paradigms. The dual contributions of this work, in terms of methodological innovations in protein purification and the discovery of competitive inhibitors, hold substantial promise for accelerating antiviral drug development, offering novel strategies to combat SARS-CoV-2 and other RNA virus infections.

## Figures and Tables

**Table 1:** Binding affinity (K_D_), association rate constant (K_on_), and dissociation rate constant (K_dis_) of Light Green SF Yellowish to N protein.

N Protein Expression Systems	K_D_ (nM)	K_on_ (1/Ms)	K_dis_ (1/s)
Eukaryotic (HEK293)	119.7 nM	9917	0.0012
Prokaryotic (*E. coli*)	19.9 × 10^3^ nM	659	0.0131

## Data Availability

Data are contained within the article and Supplementary Materials.

## Supplementary Materials

The following supporting information can be downloaded at: https://www.mdpi.com/article/10.3390/molecules30132679/s1, Figure S1: N protein dialysis results of different purification processes; Figure S2: the top 20 natural products and their derivatives; Figure S3: the top 20 approved drug molecules.

## References

[1] Li J., Lai S., Gao G.F., Shi W. (2021). The emergence, genomic diversity and global spread of SARS-CoV-2. Nature.

[2] Male V. (2022). SARS-CoV-2 infection and COVID-19 vaccination in pregnancy. Nat. Rev. Immunol..

[3] Szekely J., Mongkolprasert J., Jeayodae N., Senorit C., Chaimuti P., Swangphon P., Nanakorn N., Nualnoi T., Wongwitwichot P., Pengsakul T. (2022). Development, Analytical, and Clinical Evaluation of Rapid Immunochromatographic Antigen Test for SARS-CoV-2 Variants Detection. Diagnostics.

[4] Telenti A., Arvin A., Corey L., Corti D., Diamond M.S., García-Sastre A., Garry R.F., Holmes E.C., Pang P.S., Virgin H.W. (2021). After the pandemic: Perspectives on the future trajectory of COVID-19. Nature.

[5] El-Shabasy R.M., Nayel M.A., Taher M.M., Abdelmonem R., Shoueir K.R., Kenawy E.R. (2022). Three waves changes, new variant strains, and vaccination effect against COVID-19 pandemic. Int. J. Biol. Macromol..

[6] Mahumud R.A., Ali M.A., Kundu S., Rahman M.A., Kamara J.K., Renzaho A.M.N. (2022). Effectiveness of COVID-19 Vaccines against Delta Variant (B.1.617.2): A Meta-Analysis. Vaccines.

[7] Pormohammad A., Zarei M., Ghorbani S., Mohammadi M., Aghayari Sheikh Neshin S., Khatami A., Turner D.L., Djalalinia S., Mousavi S.A., Mardani-Fard H.A. (2022). Effectiveness of COVID-19 Vaccines against Delta (B.1.617.2) Variant: A Systematic Review and Meta-Analysis of Clinical Studies. Vaccines.

[8] Yan G., Li D., Lin Y., Fu Z., Qi H., Liu X., Zhang J., Si S., Chen Y. (2021). Development of a simple and miniaturized sandwich-like fluorescence polarization assay for rapid screening of SARS-CoV-2 main protease inhibitors. Cell Biosci..

[9] Carabelli A.M., Peacock T.P., Thorne L.G., Harvey W.T., Hughes J., de Silva T.I., Peacock S.J., Barclay W.S., de Silva T.I., Towers G.J. (2023). SARS-CoV-2 variant biology: Immune escape, transmission and fitness. Nat. Rev. Microbiol..

[10] Harvey W.T., Carabelli A.M., Jackson B., Gupta R.K., Thomson E.C., Harrison E.M., Ludden C., Reeve R., Rambaut A., COVID-19 Genomics UK (COG-UK) Consortium (2021). SARS-CoV-2 variants, spike mutations and immune escape. Nat. Rev. Microbiol..

[11] Müller K., Girl P., Giebl A., Gruetzner S., Antwerpen M., Khatamzas E., Wölfel R., von Buttlar H. (2021). Sensitivity of two SARS-CoV-2 variants with spike protein mutations to neutralising antibodies. Virus Genes.

[12] Wang Z., Schmidt F., Weisblum Y., Muecksch F., Barnes C.O., Finkin S., Schaefer-Babajew D., Cipolla M., Gaebler C., Lieberman J.A. (2021). mRNA vaccine-elicited antibodies to SARS-CoV-2 and circulating variants. Nature.

[13] Peng Y., Du N., Lei Y., Dorje S., Qi J., Luo T., Gao G.F., Song H. (2020). Structures of the SARS-CoV-2 nucleocapsid and their perspectives for drug design. EMBO J..

[14] Lu S., Ye Q., Singh D., Cao Y., Diedrich J.K., Yates J.R., Villa E., Cleveland D.W., Corbett K.D. (2021). The SARS-CoV-2 nucleocapsid phosphoprotein forms mutually exclusive condensates with RNA and the membrane-associated M protein. Nat. Commun..

[15] Wu W., Cheng Y., Zhou H., Sun C., Zhang S. (2023). The SARS-CoV-2 nucleocapsid protein: Its role in the viral life cycle, structure and functions, and use as a potential target in the development of vaccines and diagnostics. Virol. J..

[16] V’kovski P., Kratzel A., Steiner S., Stalder H., Thiel V. (2021). Coronavirus biology and replication: Implications for SARS-CoV-2. Nat. Rev. Microbiol..

[17] Baggen J., Vanstreels E., Jansen S., Daelemans D. (2021). Cellular host factors for SARS-CoV-2 infection. Nat. Microbiol..

[18] Emrani J., Ahmed M., Jeffers-Francis L., Teleha J.C., Mowa N., Newman R.H., Thomas M.D. (2021). SARS-COV-2, infection, transmission, transcription, translation, proteins, and treatment: A review. Int. J. Biol. Macromol..

[19] Mohan A., Rendine N., Mohammed M.K.S., Jeeva A., Ji H.F., Talluri V.R. (2022). Structure-based virtual screening, in silico docking, ADME properties prediction and molecular dynamics studies for the identification of potential inhibitors against SARS-CoV-2 M(pro). Mol. Divers..

[20] Nazir F., John Kombe Kombe A., Khalid Z., Bibi S., Zhang H., Wu S., Jin T. (2024). SARS-CoV-2 replication and drug discovery. Mol. Cell. Probes.

[21] Luan X., Li X., Li Y., Su G., Yin W., Jiang Y., Xu N., Wang F., Cheng W., Jin Y. (2022). Antiviral drug design based on structural insights into the N-terminal domain and C-terminal domain of the SARS-CoV-2 nucleocapsid protein. Sci. Bull..

[22] Bai Z., Cao Y., Liu W., Li J. (2021). The SARS-CoV-2 Nucleocapsid Protein and Its Role in Viral Structure, Biological Functions, and a Potential Target for Drug or Vaccine Mitigation. Viruses.

[23] Wang W., Chen J., Yu X., Lan H.Y. (2022). Signaling mechanisms of SARS-CoV-2 Nucleocapsid protein in viral infection, cell death and inflammation. Int. J. Biol. Sci..

[24] Supekar N.T., Shajahan A., Gleinich A.S., Rouhani D.S., Heiss C., Chapla D.G., Moremen K.W., Azadi P. (2021). Variable posttranslational modifications of severe acute respiratory syndrome coronavirus 2 nucleocapsid protein. Glycobiology.

[25] Royster A., Ren S., Ma Y., Pintado M., Kahng E., Rowan S., Mir S., Mir M. (2023). SARS-CoV-2 Nucleocapsid Protein Is a Potential Therapeutic Target for Anticoronavirus Drug Discovery. Microbiol. Spectr..

[26] Kumari S., Mistry H., Bihani S.C., Mukherjee S.P., Gupta G.D. (2024). Unveiling potential inhibitors targeting the nucleocapsid protein of SARS-CoV-2: Structural insights into their binding sites. Int. J. Biol. Macromol..

[27] Chen Y.M., Wei J.L., Qin R.S., Hou J.P., Zang G.C., Zhang G.Y., Chen T.T. (2022). Folic acid: A potential inhibitor against SARS-CoV-2 nucleocapsid protein. Pharm. Biol..

[28] Sekine R., Tsuno S., Irokawa H., Sumitomo K., Han T., Sato Y., Nishizawa S., Takeda K., Kuge S. (2023). Inhibition of SARS-CoV-2 nucleocapsid protein-RNA interaction by guanosine oligomeric RNA. J. Biochem..

[29] Han H., Gracia A.V., Røise J.J., Boike L., Leon K., Schulze-Gahmen U., Stentzel M.R., Bajaj T., Chen D., Li I.C. (2023). A covalent inhibitor targeting the papain-like protease from SARS-CoV-2 inhibits viral replication. RSC Adv..

[30] Forsythe H.M., Galvan J.R., Yu Z., Pinckney S., Reardon P., Cooley R.B., Zhu P., Rolland A.D., Prell J.S., Barbar E. (2021). Multivalent binding of the partially disordered SARS-CoV-2 nucleocapsid phosphoprotein dimer to RNA. Biophys. J..

[31] Jack A., Ferro L., Trnka M., Wehri E., Nadgir A., Nguyenla X., Fox D., Costa K., Stanley S., Schaletzky J. (2021). SARS-CoV-2 nucleocapsid protein forms condensates with viral genomic RNA. PLoS Biol..

[32] Tarczewska A., Kolonko-Adamska M., Zarębski M., Dobrucki J., Ożyhar A., Greb-Markiewicz B. (2021). The method utilized to purify the SARS-CoV-2 N protein can affect its molecular properties. Int. J. Biol. Macromol..

[33] Matsuo T. (2021). Viewing SARS-CoV-2 Nucleocapsid Protein in Terms of Molecular Flexibility. Biology.

[34] Zhao D., Xu W., Zhang X., Wang X., Ge Y., Yuan E., Xiong Y., Wu S., Li S., Wu N. (2021). Understanding the phase separation characteristics of nucleocapsid protein provides a new therapeutic opportunity against SARS-CoV-2. Protein Cell.

[35] Zhao M., Yu Y., Sun L.-M., Xing J.-Q., Li T., Zhu Y., Wang M., Yu Y., Xue W., Xia T. (2021). GCG inhibits SARS-CoV-2 replication by disrupting the liquid phase condensation of its nucleocapsid protein. Nat. Commun..

[36] Maiti A., Matsuo H. (2024). Affinity Tag-Free Purification of SARS-CoV-2 N Protein and Its Crystal Structure in Complex with ssDNA. Biomolecules.

[37] De Vos J., Pereira Aguilar P., Köppl C., Fischer A., Grünwald-Gruber C., Dürkop M., Klausberger M., Mairhofer J., Striedner G., Cserjan-Puschmann M. (2021). Production of full-length SARS-CoV-2 nucleocapsid protein from *Escherichia coli* optimized by native hydrophobic interaction chromatography hyphenated to multi-angle light scattering detection. Talanta.

[38] Di D., Dileepan M., Ahmed S., Liang Y., Ly H. (2021). Recombinant SARS-CoV-2 Nucleocapsid Protein: Expression, Purification, and Its Biochemical Characterization and Utility in Serological Assay Development to Assess Immunological Responses to SARS-CoV-2 Infection. Pathogens.

[39] Li G., Li W., Fang X., Song X., Teng S., Ren Z., Hu D., Zhou S., Wu G., Li K. (2021). Expression and purification of recombinant SARS-CoV-2 nucleocapsid protein in inclusion bodies and its application in serological detection. Protein Expr. Purif..

[40] Burgess R. (2009). Protein precipitation techniques. Methods Enzymol..

[41] Duellman S.J., Burgess R.R. (2009). Large-scale Epstein–Barr virus EBNA1 protein purification. Protein Expr. Purif..

[42] Feng J., Li F.Q., Li Q., Hu H.L., Hong G.F. (2002). Expression and purification of Rhizobium leguminosarum NodD. Protein Expr. Purif..

[43] Fong B.A., Gillies A.R., Ghazi I., LeRoy G., Lee K.C., Westblade L.F., Wood D.W. (2010). Purification of Escherichia coli RNA polymerase using a self-cleaving elastin-like polypeptide tag. Protein Sci..

[44] (2022). Molecular Operating Environment (MOE), 2022.02.

[45] Dubrow A., Zuniga B., Topo E., Cho J.H. (2022). Suppressing Nonspecific Binding in Biolayer Interferometry Experiments for Weak Ligand-Analyte Interactions. ACS Omega.

[46] Murali S., Rustandi R.R., Zheng X., Payne A., Shang L. (2022). Applications of Surface Plasmon Resonance and Biolayer Interferometry for Virus-Ligand Binding. Viruses.

[47] Shi Q., Guo W., Shen Q., Han J., Lei L., Chen L., Yang L., Feng C., Zhou B. (2021). In vitro biolayer interferometry analysis of acetylcholinesterase as a potential target of aryl-organophosphorus flame-retardants. J. Hazard. Mater..

[48] Sanz-Martinez I., Pereira S., Merino P., Corzana F., Hurtado-Guerrero R. (2023). Molecular Recognition of GalNAc in Mucin-Type O-Glycosylation. Acc. Chem. Res..

[49] Kinarsky L., Suryanarayanan G., Prakash O., Paulsen H., Clausen H., Hanisch F.G., Hollingsworth M.A., Sherman S. (2003). Conformational studies on the MUC1 tandem repeat glycopeptides: Implication for the enzymatic O-glycosylation of the mucin protein core. Glycobiology.

[50] Grifoni A., Sidney J., Zhang Y., Scheuermann R.H., Peters B., Sette A. (2020). A Sequence Homology and Bioinformatic Approach Can Predict Candidate Targets for Immune Responses to SARS-CoV-2. Cell Host Microbe.

[51] Kang S., Yang M., Hong Z., Zhang L., Huang Z., Chen X., He S., Zhou Z., Zhou Z., Chen Q. (2020). Crystal structure of SARS-CoV-2 nucleocapsid protein RNA binding domain reveals potential unique drug targeting sites. Acta Pharm. Sinica B.

[52] Yasui F., Kai C., Kitabatake M., Inoue S., Yoneda M., Yokochi S., Kase R., Sekiguchi S., Morita K., Hishima T. (2008). Prior immunization with severe acute respiratory syndrome (SARS)-associated coronavirus (SARS-CoV) nucleocapsid protein causes severe pneumonia in mice infected with SARS-CoV. J. Immunol..

[53] Rakib A., Sami S.A., Islam M.A., Ahmed S., Faiz F.B., Khanam B.H., Marma K.K.S., Rahman M., Uddin M.M.N., Nainu F. (2020). Epitope-Based Immunoinformatics Approach on Nucleocapsid Protein of Severe Acute Respiratory Syndrome-Coronavirus-2. Molecules.

[54] He J., Huang J.R., Zhang Y.L., Zhang J. (2021). SARS-CoV-2 nucleocapsid protein intranasal inoculation induces local and systemic T cell responses in mice. J. Med. Virol..

[55] Bhattacharya M., Sharma A.R., Patra P., Ghosh P., Sharma G., Patra B.C., Lee S.S., Chakraborty C. (2020). Development of epitope-based peptide vaccine against novel coronavirus 2019 (SARS-CoV-2): Immunoinformatics approach. J. Med. Virol..

[56] Chukwudozie O.S., Chukwuanukwu R.C., Iroanya O.O., Eze D.M., Duru V.C., Dele-Alimi T.O., Kehinde B.D., Bankole T.T., Obi P.C., Okinedo E.U. (2020). Attenuated Subcomponent Vaccine Design Targeting the SARS-CoV-2 Nucleocapsid Phosphoprotein RNA Binding Domain: In Silico Analysis. J. Immunol. Res..

[57] Oliveira S.C., de Magalhães M.T.Q., Homan E.J. (2020). Immunoinformatic Analysis of SARS-CoV-2 Nucleocapsid Protein and Identification of COVID-19 Vaccine Targets. Front. Immunol..

[58] Zinzula L., Basquin J., Bohn S., Beck F., Klumpe S., Pfeifer G., Nagy I., Bracher A., Hartl F.U., Baumeister W. (2021). High-resolution structure and biophysical characterization of the nucleocapsid phosphoprotein dimerization domain from the COVID-19 severe acute respiratory syndrome coronavirus 2. Biochem. Biophys. Res. Commun..

[59] Perdikari T.M., Murthy A.C., Ryan V.H., Watters S., Naik M.T., Fawzi N.L. (2020). SARS-CoV-2 nucleocapsid protein phase-separates with RNA and with human hnRNPs. EMBO J..

[60] Zeng W., Liu G., Ma H., Zhao D., Yang Y., Liu M., Mohammed A., Zhao C., Yang Y., Xie J. (2020). Biochemical characterization of SARS-CoV-2 nucleocapsid protein. Biochem. Biophys. Res. Commun..

[61] Ye Q., West A.M.V., Silletti S., Corbett K.D. (2020). Architecture and self-assembly of the SARS-CoV-2 nucleocapsid protein. Protein Sci. A Publ. Protein Soc..

[62] Botova M., Camacho-Zarco A.R., Tognetti J., Bessa L.M., Guseva S., Mikkola E., Salvi N., Maurin D., Herrmann T., Blackledge M. (2024). A specific phosphorylation-dependent conformational switch in SARS-CoV-2 nucleocapsid protein inhibits RNA binding. Sci. Adv..

