# Supplementary material for: Purification and Inhibitor Screening of the Full-Length SARS-CoV-2 Nucleocapsid Protein
Source: Molecules. 2025 Jun 20;30(13):2679. doi: 10.3390/molecules30132679 (PMC12251317; doi:10.3390/molecules30132679)
Supplement: Supplementary file 1 [file molecules-30-02679-s001.zip › molecules-3608758-supplementary/Drug docking result-top1000.pdf]

1

DrugBank ID: DB14099

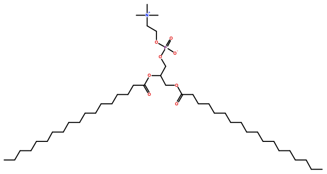

Docking Score: -9.3441

2

DrugBank ID: DB11284

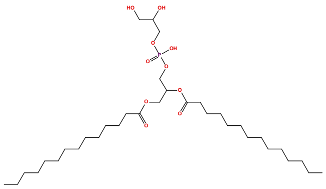

Docking Score: -8.8725

3

DrugBank ID: DB06811

Docking Score: -8.6384

4

DrugBank ID: DB09065

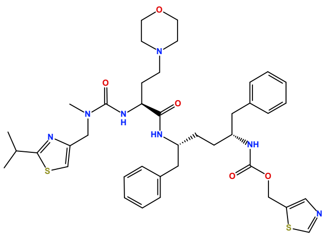

Docking Score: -8.4982

5

DrugBank ID: DB11183

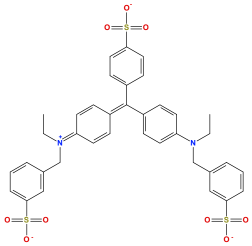

Docking Score: -8.3788

6

DrugBank ID: DB06804

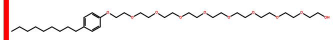

Docking Score: -8.3608

7

DrugBank ID: DB11660

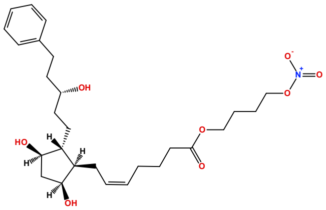

Docking Score: -8.2228

8

DrugBank ID: DB14879

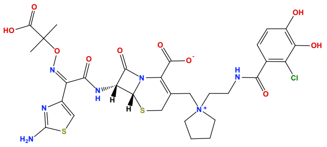

Docking Score: -8.0873

9

DrugBank ID: DB00385

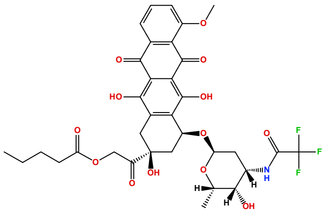

Docking Score: -8.0511

10

DrugBank ID: DB08909

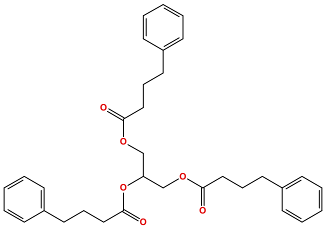

Docking Score: -7.9994

11

DrugBank ID: DB14185

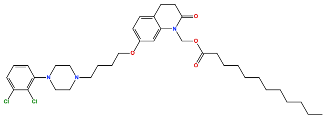

Docking Score: -7.9676

12

DrugBank ID: DB01167

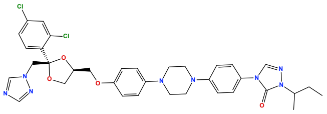

Docking Score: -7.9113

13

DrugBank ID: DB00944

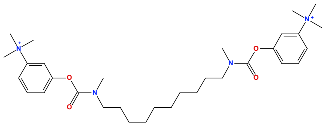

Docking Score: -7.9059

14

DrugBank ID: DB00390

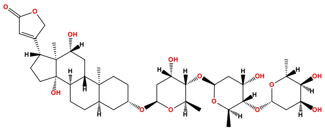

Docking Score: -7.8543

15

DrugBank ID: DB00430

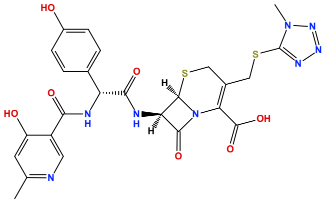

Docking Score: -7.8461

16

DrugBank ID: DB11206

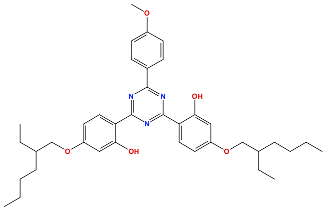

Docking Score: -7.8376

17

DrugBank ID: DB12500

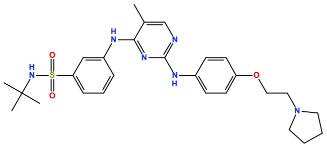

Docking Score: -7.8245

18

DrugBank ID: DB01232

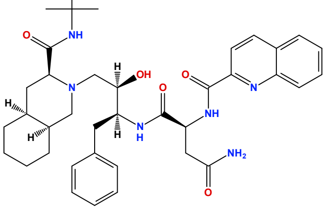

Docking Score: -7.7796

19

DrugBank ID: DB11986

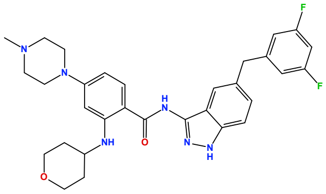

Docking Score: -7.7719

20

DrugBank ID: DB06636

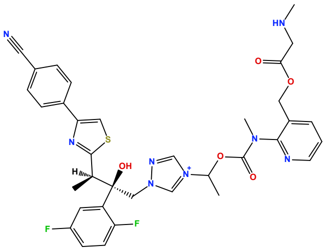

Docking Score: -7.7539

21

DrugBank ID: DB00868

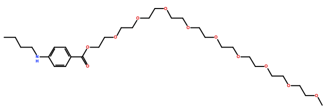

Docking Score: -7.6609

22

DrugBank ID: DB08884

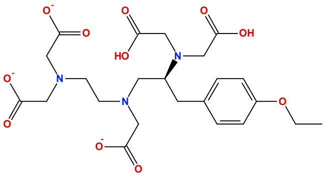

Docking Score: -7.6585

23

DrugBank ID: DB14143

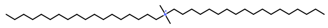

Docking Score: -7.6517

24

DrugBank ID: DB11190

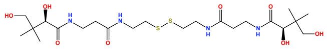

Docking Score: -7.6510

25

DrugBank ID: DB08818

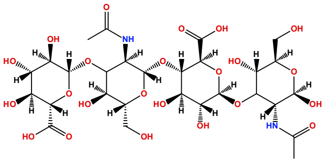

Docking Score: -7.6280

26

DrugBank ID: DB00948

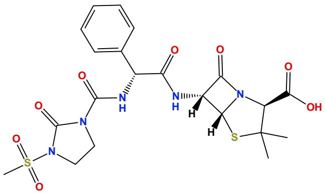

Docking Score: -7.6089

27

DrugBank ID: DB09049

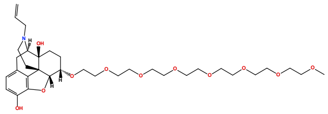

Docking Score: -7.5785

28

DrugBank ID: DB06695

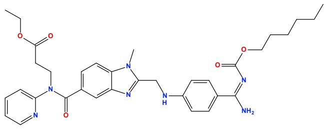

Docking Score: -7.5611

29

DrugBank ID: DB09050

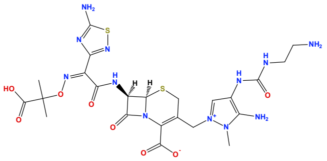

Docking Score: -7.5610

30

DrugBank ID: DB11995

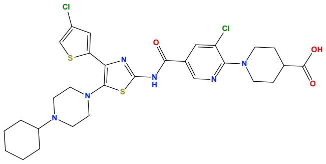

Docking Score: -7.5487

31

DrugBank ID: DB15822

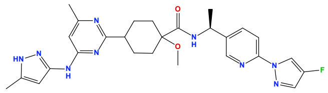

Docking Score: -7.5220

32

DrugBank ID: DB06796

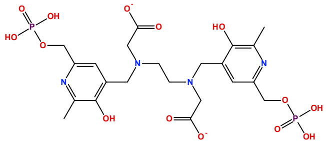

Docking Score: -7.4982

33

DrugBank ID: DB09552

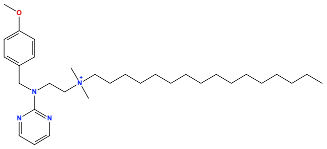

Docking Score: -7.4979

34

DrugBank ID: DB06590

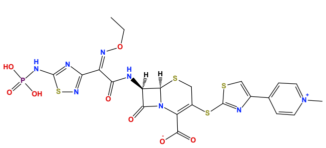

Docking Score: -7.4851

35

DrugBank ID: DB09079

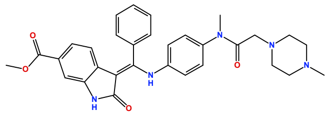

Docking Score: -7.4785

36

DrugBank ID: DB00460

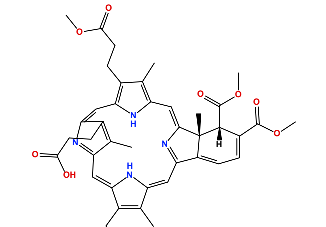

Docking Score: -7.4745

37

DrugBank ID: DB13265

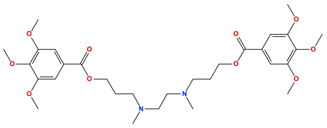

Docking Score: -7.4733

38

DrugBank ID: DB13947

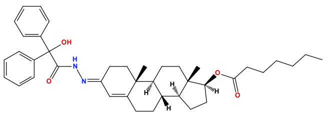

Docking Score: -7.4689

39

DrugBank ID: DB11637

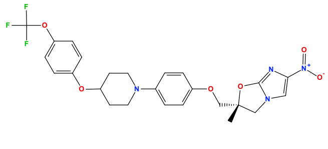

Docking Score: -7.4323

40

DrugBank ID: DB00444

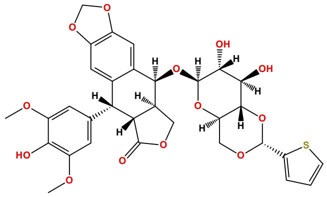

Docking Score: -7.4053

41

DrugBank ID: DB09102

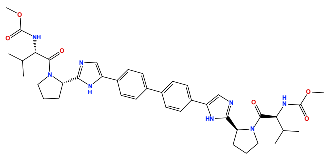

Docking Score: -7.3920

42

DrugBank ID: DB00826

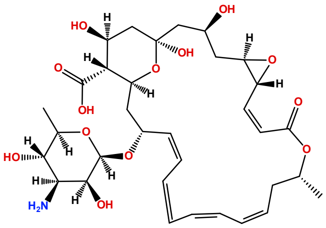

Docking Score: -7.3596

43

DrugBank ID: DB01016

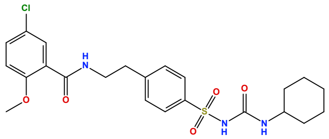

Docking Score: -7.3435

44

DrugBank ID: DB12615

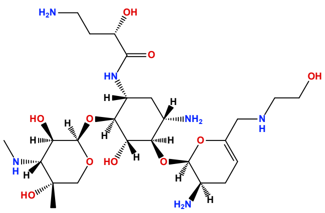

Docking Score: -7.3190

45

DrugBank ID: DB03147

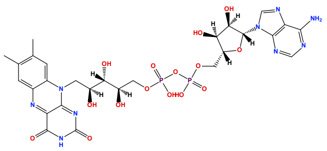

Docking Score: -7.2800

46

DrugBank ID: DB00157

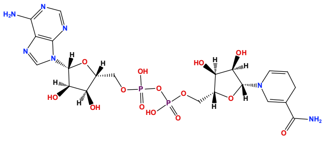

Docking Score: -7.2751

47

DrugBank ID: DB00796

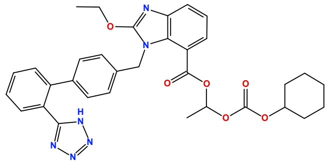

Docking Score: -7.2446

48

DrugBank ID: DB00538

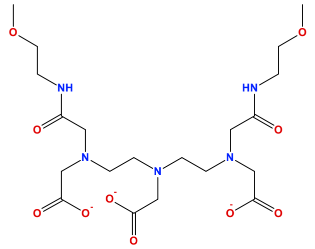

Docking Score: -7.2207

49

DrugBank ID: DB00410

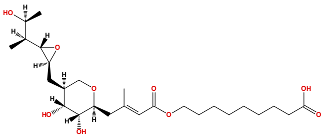

Docking Score: -7.2191

50

DrugBank ID: DB08871

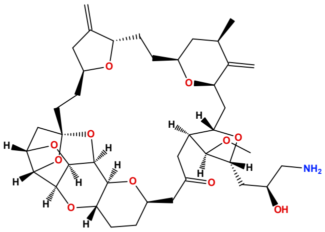

Docking Score: -7.2177

51

DrugBank ID: DB01051

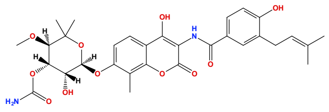

Docking Score: -7.2141

52

DrugBank ID: DB00688

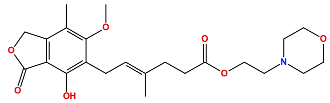

Docking Score: -7.2112

53

DrugBank ID: DB06137

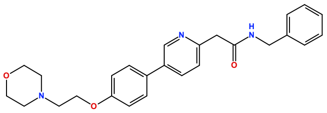

Docking Score: -7.2012

54

DrugBank ID: DB01212

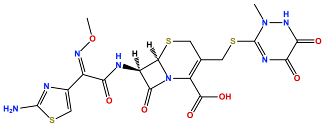

Docking Score: -7.1923

55

DrugBank ID: DB14658

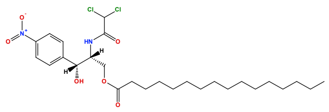

Docking Score: -7.1880

56

DrugBank ID: DB01328

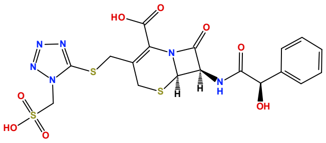

Docking Score: -7.1848

57

DrugBank ID: DB00229

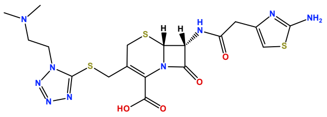

Docking Score: -7.1814

58

DrugBank ID: DB05521

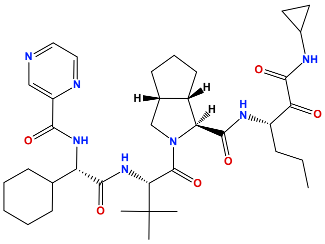

Docking Score: -7.1791

59

DrugBank ID: DB13955

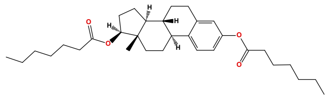

Docking Score: -7.1599

60

DrugBank ID: DB13345

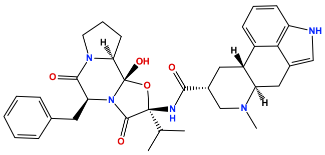

Docking Score: -7.1443

61

DrugBank ID: DB00183

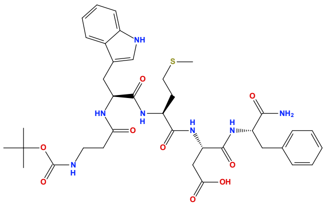

Docking Score: -7.1437

62

DrugBank ID: DB09082

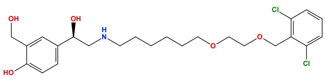

Docking Score: -7.1332

63

DrugBank ID: DB06684

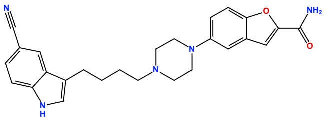

Docking Score: -7.1241

64

DrugBank ID: DB00650

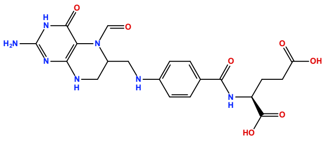

Docking Score: -7.1233

65

DrugBank ID: DB09335

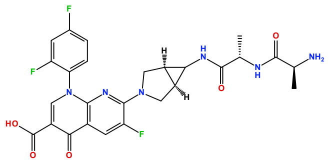

Docking Score: -7.1189

66

DrugBank ID: DB01764

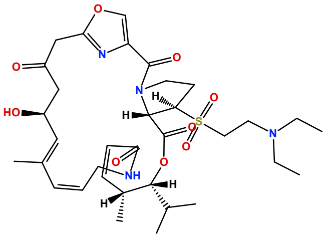

Docking Score: -7.1070

67

DrugBank ID: DB12001

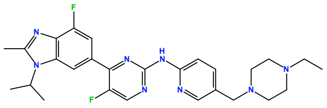

Docking Score: -7.1061

68

DrugBank ID: DB00206

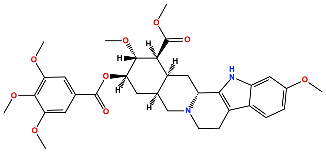

Docking Score: -7.0944

69

DrugBank ID: DB11652

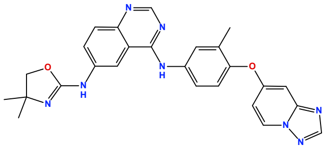

Docking Score: -7.0905

70

DrugBank ID: DB00274

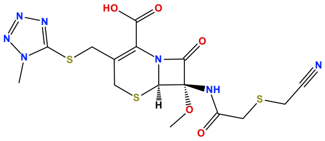

Docking Score: -7.0725

71

DrugBank ID: DB11362

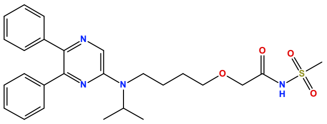

Docking Score: -7.0690

72

DrugBank ID: DB08934

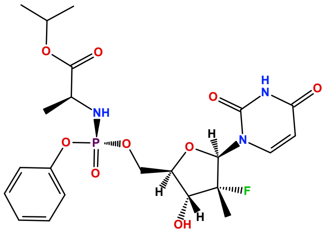

Docking Score: -7.0515

73

DrugBank ID: DB04703

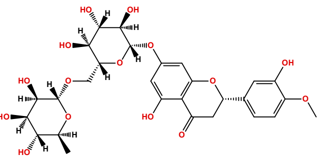

Docking Score: -7.0305

74

DrugBank ID: DB01122

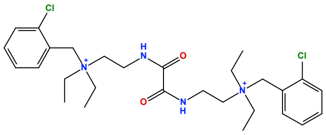

Docking Score: -7.0243

75

DrugBank ID: DB14001

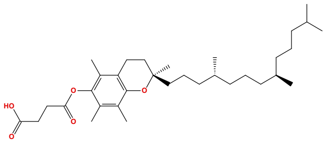

Docking Score: -7.0176

76

DrugBank ID: DB01082

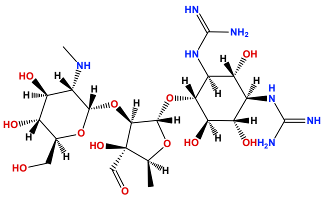

Docking Score: -7.0169

77

DrugBank ID: DB08822

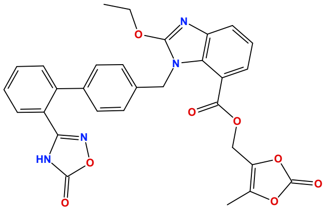

Docking Score: -6.9966

78

DrugBank ID: DB00492

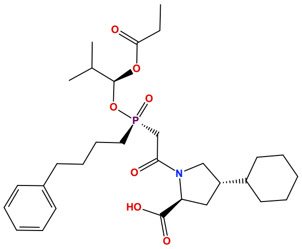

Docking Score: -6.9950

79

DrugBank ID: DB11855

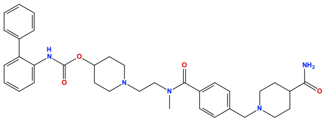

Docking Score: -6.9910

80

DrugBank ID: DB01211

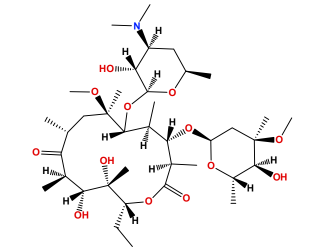

Docking Score: -6.9872

81

DrugBank ID: DB00158

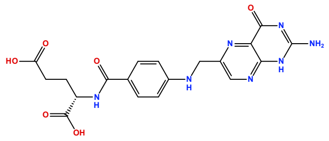

Docking Score: -6.9850

82

DrugBank ID: DB04890

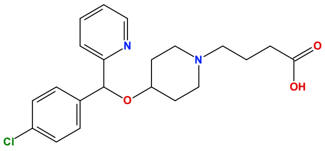

Docking Score: -6.9839

83

DrugBank ID: DB04570

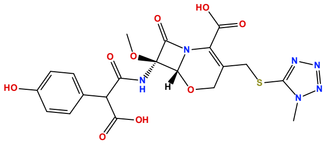

Docking Score: -6.9830

84

DrugBank ID: DB09026

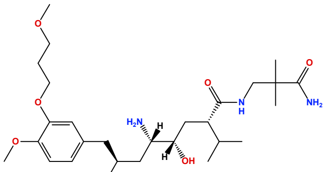

Docking Score: -6.9774

85

DrugBank ID: DB15982

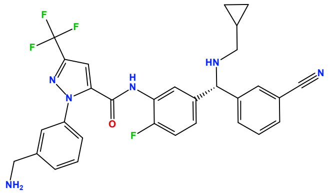

Docking Score: -6.9746

86

DrugBank ID: DB08932

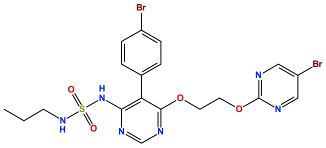

Docking Score: -6.9692

87

DrugBank ID: DB15233

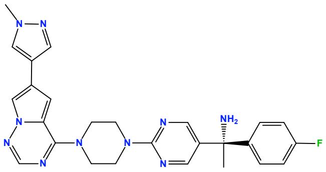

Docking Score: -6.9601

88

DrugBank ID: DB13967

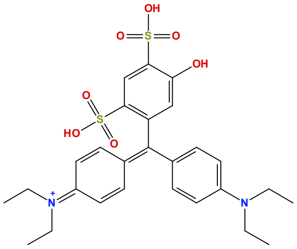

Docking Score: -6.9529

89

DrugBank ID: DB00878

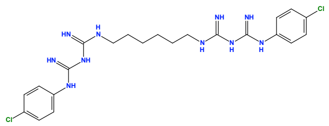

Docking Score: -6.9457

90

DrugBank ID: DB06717

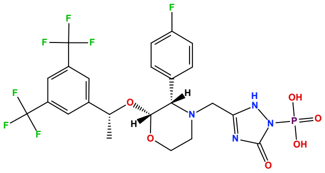

Docking Score: -6.9377

91

DrugBank ID: DB13125

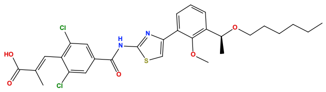

Docking Score: -6.9363

92

DrugBank ID: DB00947

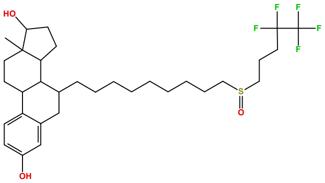

Docking Score: -6.9318

93

DrugBank ID: DB11703

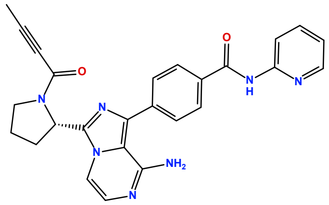

Docking Score: -6.9181

94

DrugBank ID: DB00559

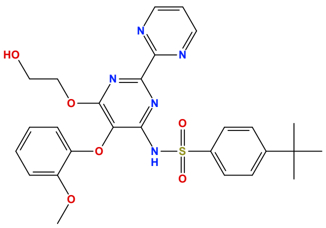

Docking Score: -6.9149

95

DrugBank ID: DB11231

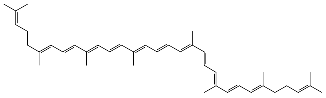

Docking Score: -6.9122

96

DrugBank ID: DB00284

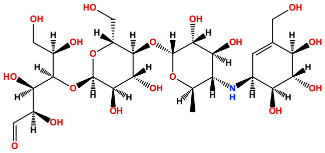

Docking Score: -6.8896

97

DrugBank ID: DB06290

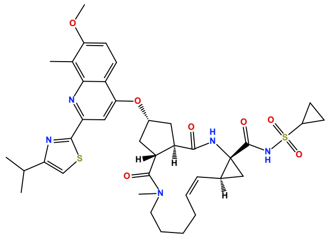

Docking Score: -6.8882

98

DrugBank ID: DB11796

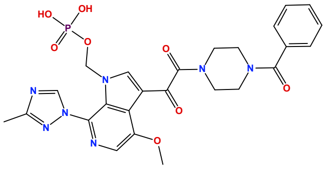

Docking Score: -6.8875

99

DrugBank ID: DB06441

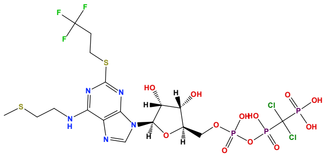

Docking Score: -6.8813

100

DrugBank ID: DB01604

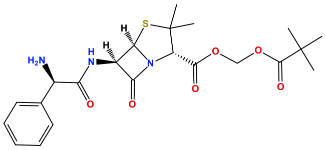

Docking Score: -6.8649

101

DrugBank ID: DB08901

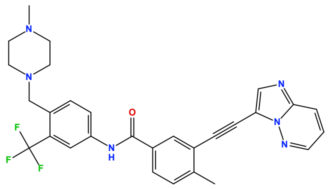

Docking Score: -6.8599

102

DrugBank ID: DB00671

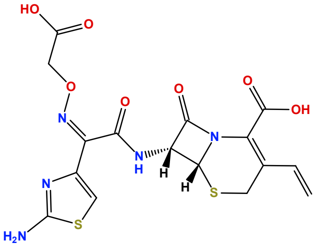

Docking Score: -6.8598

103

DrugBank ID: DB00746

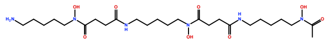

Docking Score: -6.8588

104

DrugBank ID: DB11586

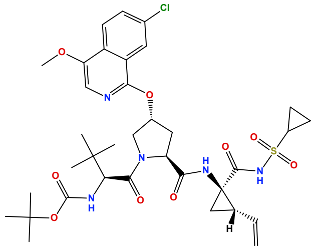

Docking Score: -6.8420

105

DrugBank ID: DB09272

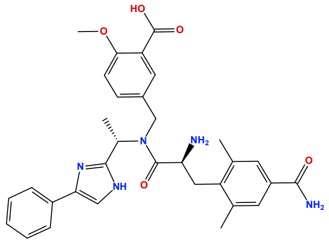

Docking Score: -6.8418

106

DrugBank ID: DB00775

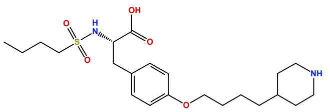

Docking Score: -6.8399

107

DrugBank ID: DB01067

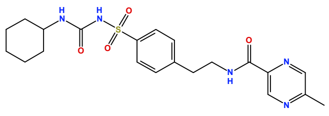

Docking Score: -6.8362

108

DrugBank ID: DB14761

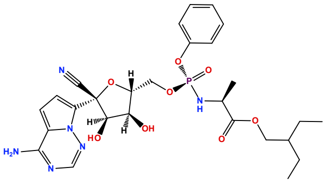

Docking Score: -6.8357

109

DrugBank ID: DB01601

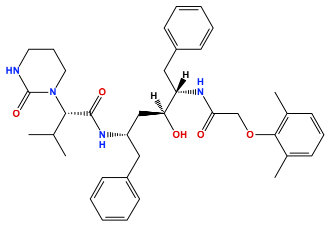

Docking Score: -6.8288

110

DrugBank ID: DB00493

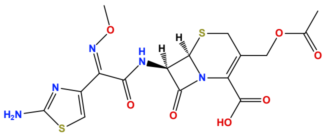

Docking Score: -6.8182

111

DrugBank ID: DB14019

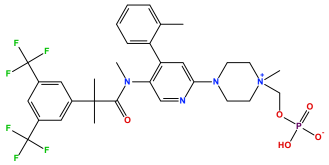

Docking Score: -6.8097

112

DrugBank ID: DB00319

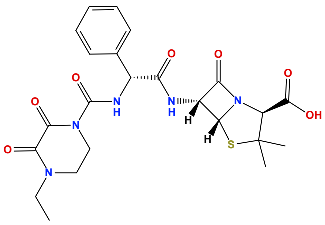

Docking Score: -6.8005

113

DrugBank ID: DB12434

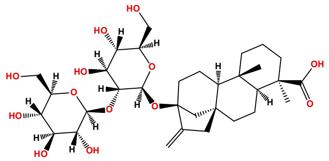

Docking Score: -6.7968

114

DrugBank ID: DB00862

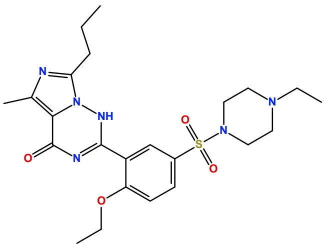

Docking Score: -6.7965

115

DrugBank ID: DB14703

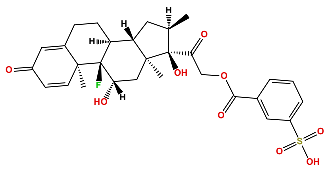

Docking Score: -6.7959

116

DrugBank ID: DB09374

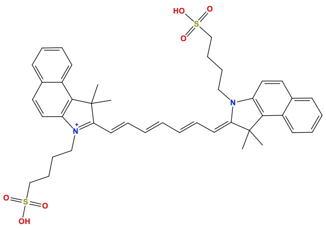

Docking Score: -6.7910

117

DrugBank ID: DB00222

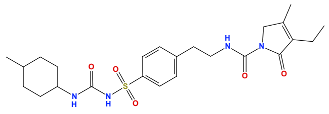

Docking Score: -6.7895

118

DrugBank ID: DB01022

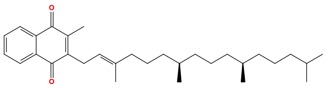

Docking Score: -6.7881

119

DrugBank ID: DB00881

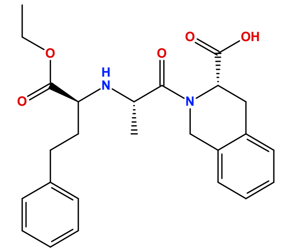

Docking Score: -6.7805

120

DrugBank ID: DB15035

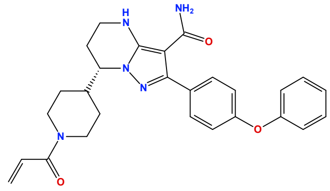

Docking Score: -6.7803

121

DrugBank ID: DB12127

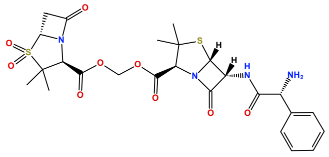

Docking Score: -6.7755

122

DrugBank ID: DB12674

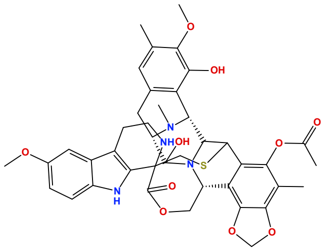

Docking Score: -6.7733

123

DrugBank ID: DB06207

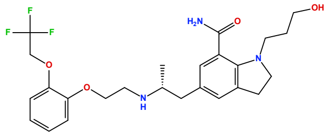

Docking Score: -6.7678

124

DrugBank ID: DB01326

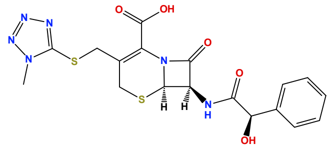

Docking Score: -6.7606

125

DrugBank ID: DB14895

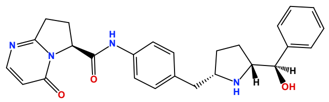

Docking Score: -6.7481

126

DrugBank ID: DB08889

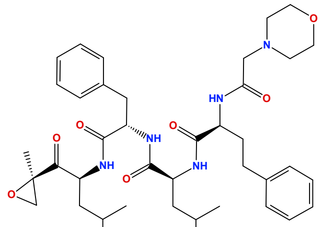

Docking Score: -6.7431

127

DrugBank ID: DB15685

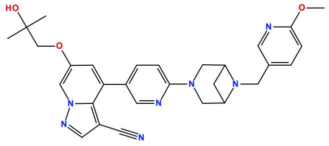

Docking Score: -6.7375

128

DrugBank ID: DB00762

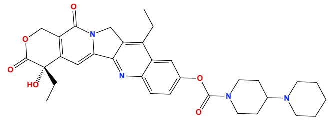

Docking Score: -6.7332

129

DrugBank ID: DB00479

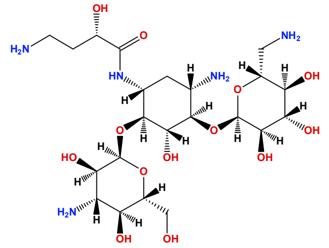

Docking Score: -6.7261

130

DrugBank ID: DB00256

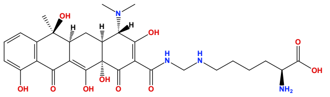

Docking Score: -6.7226

131

DrugBank ID: DB01319

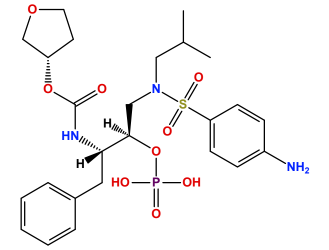

Docking Score: -6.7220

132

DrugBank ID: DB12095

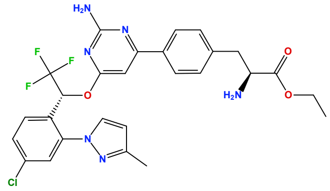

Docking Score: -6.7182

133

DrugBank ID: DB04348

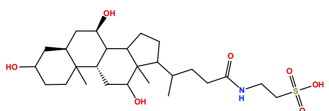

Docking Score: -6.7101

134

DrugBank ID: DB00287

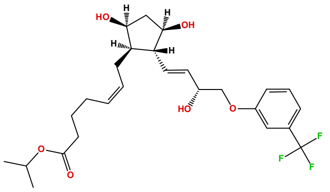

Docking Score: -6.7095

135

DrugBank ID: DB12887

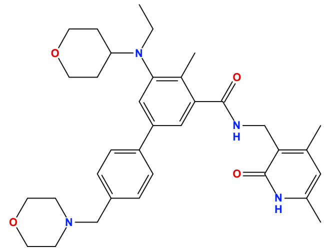

Docking Score: -6.7081

136

DrugBank ID: DB12141

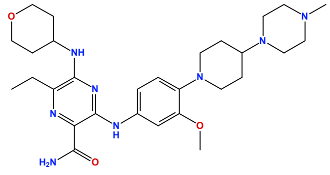

Docking Score: -6.6985

137

DrugBank ID: DB00619

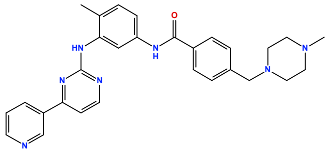

Docking Score: -6.6984

138

DrugBank ID: DB11585

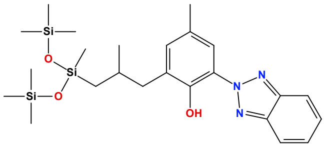

Docking Score: -6.6928

139

DrugBank ID: DB09128

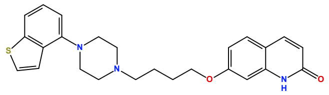

Docking Score: -6.6907

140

DrugBank ID: DB01238

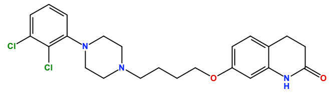

Docking Score: -6.6847

141

DrugBank ID: DB11828

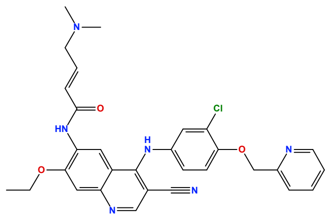

Docking Score: -6.6834

142

DrugBank ID: DB00568

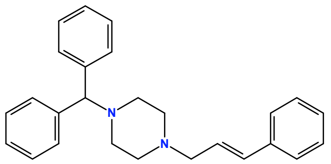

Docking Score: -6.6808

143

DrugBank ID: DB01083

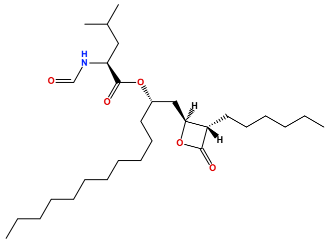

Docking Score: -6.6783

144

DrugBank ID: DB08873

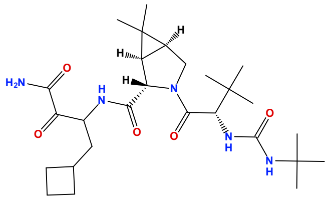

Docking Score: -6.6741

145

DrugBank ID: DB01259

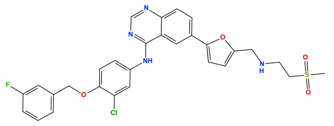

Docking Score: -6.6737

146

DrugBank ID: DB11742

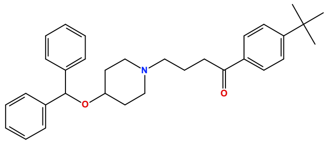

Docking Score: -6.6731

147

DrugBank ID: DB00293

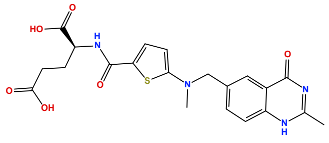

Docking Score: -6.6699

148

DrugBank ID: DB00300

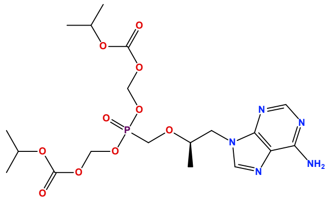

Docking Score: -6.6682

149

DrugBank ID: DB12329

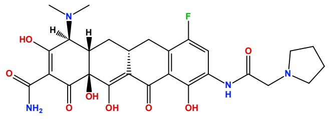

Docking Score: -6.6677

150

DrugBank ID: DB08816

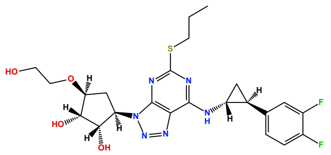

Docking Score: -6.6638

151

DrugBank ID: DB11718

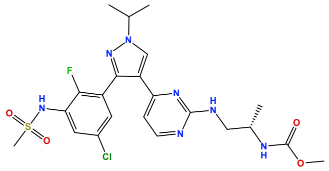

Docking Score: -6.6608

152

DrugBank ID: DB00549

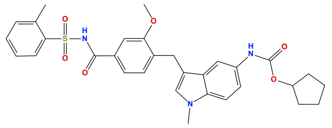

Docking Score: -6.6606

153

DrugBank ID: DB01329

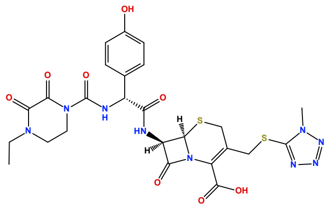

Docking Score: -6.6595

154

DrugBank ID: DB09042

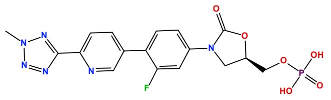

Docking Score: -6.6552

155

DrugBank ID: DB00895

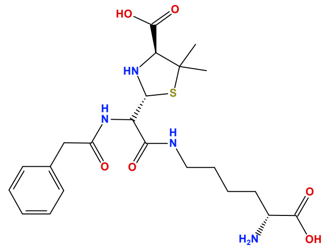

Docking Score: -6.6540

156

DrugBank ID: DB00905

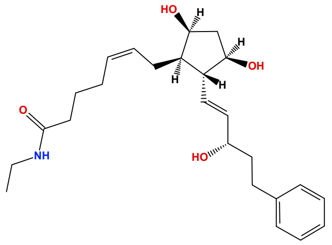

Docking Score: -6.6472

157

DrugBank ID: DB09075

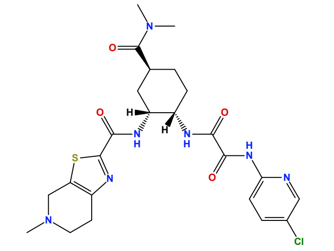

Docking Score: -6.6468

158

DrugBank ID: DB12492

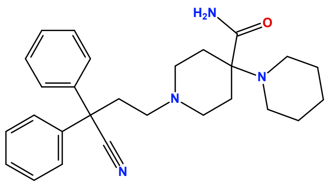

Docking Score: -6.6346

159

DrugBank ID: DB09039

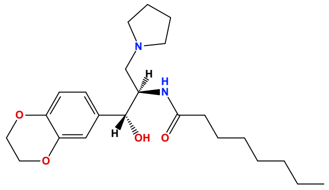

Docking Score: -6.6300

160

DrugBank ID: DB09330

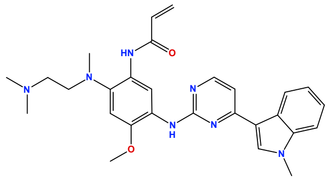

Docking Score: -6.6291

161

DrugBank ID: DB00560

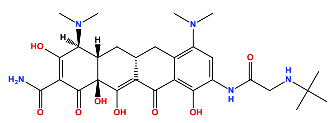

Docking Score: -6.6285

162

DrugBank ID: DB00204

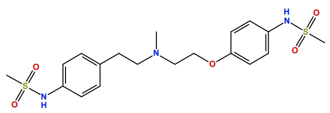

Docking Score: -6.6217

163

DrugBank ID: DB09038

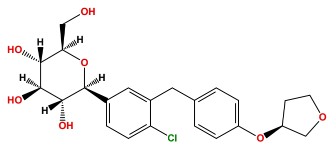

Docking Score: -6.6213

164

DrugBank ID: DB00966

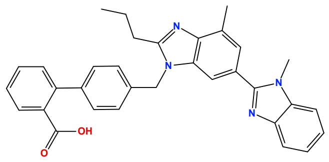

Docking Score: -6.6207

165

DrugBank ID: DB12839

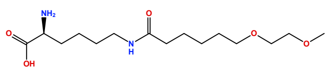

Docking Score: -6.6198

166

DrugBank ID: DB12010

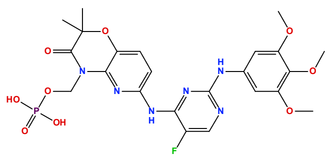

Docking Score: -6.6148

167

DrugBank ID: DB11575

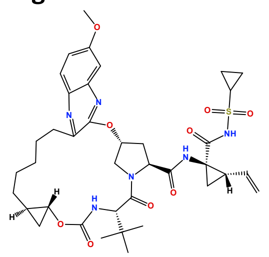

Docking Score: -6.6138

168

DrugBank ID: DB06616

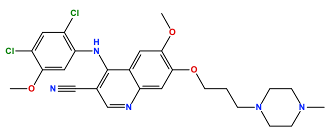

Docking Score: -6.6121

169

DrugBank ID: DB00137

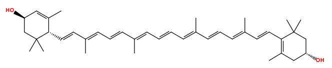

Docking Score: -6.6049

170

DrugBank ID: DB06755

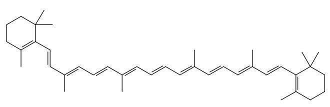

Docking Score: -6.6045

171

DrugBank ID: DB06809

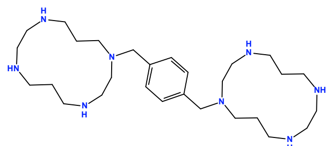

Docking Score: -6.6041

172

DrugBank ID: DB09030

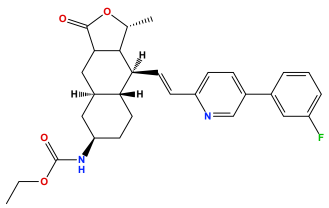

Docking Score: -6.5999

173

DrugBank ID: DB00622

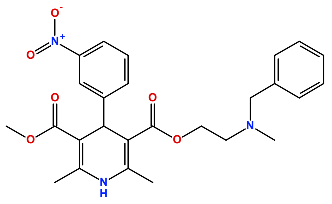

Docking Score: -6.5941

174

DrugBank ID: DB11853

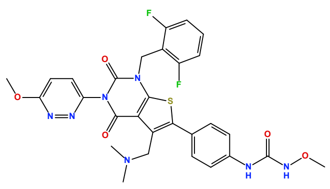

Docking Score: -6.5931

175

DrugBank ID: DB06813

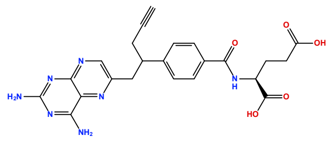

Docking Score: -6.5885

176

DrugBank ID: DB06249

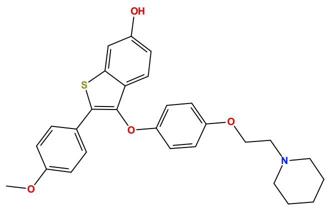

Docking Score: -6.5865

177

DrugBank ID: DB13931

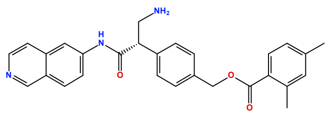

Docking Score: -6.5831

178

DrugBank ID: DB01112

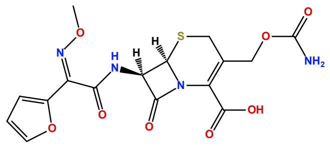

Docking Score: -6.5777

179

DrugBank ID: DB00278

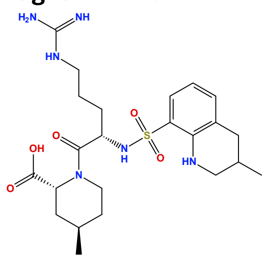

Docking Score: -6.5774

180

DrugBank ID: DB09143

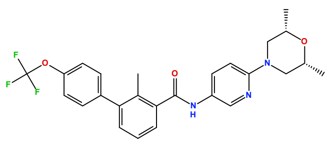

Docking Score: -6.5763

181

DrugBank ID: DB11611

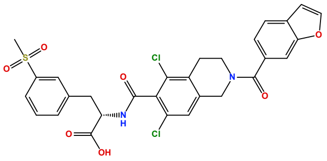

Docking Score: -6.5760

182

DrugBank ID: DB09297

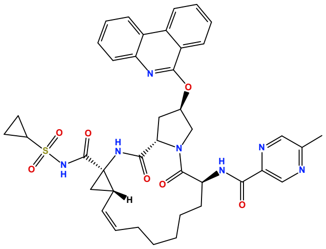

Docking Score: -6.5757

183

DrugBank ID: DB15328

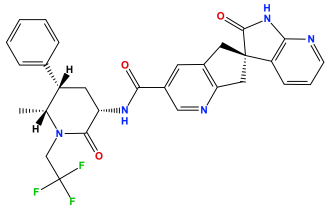

Docking Score: -6.5754

184

DrugBank ID: DB04855

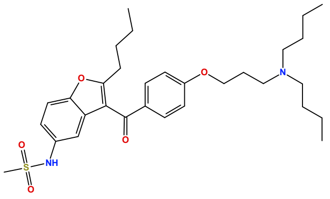

Docking Score: -6.5746

185

DrugBank ID: DB00597

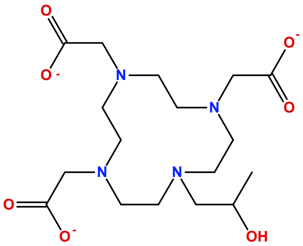

Docking Score: -6.5728

186

DrugBank ID: DB12532

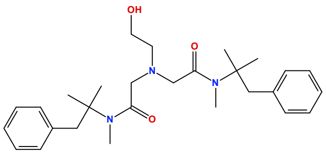

Docking Score: -6.5688

187

DrugBank ID: DB00654

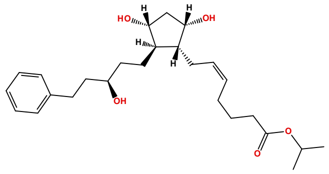

Docking Score: -6.5669

188

DrugBank ID: DB11431

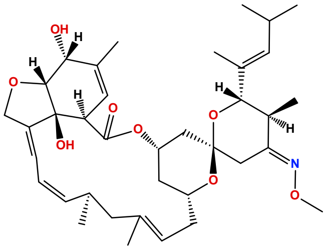

Docking Score: -6.5621

189

DrugBank ID: DB09238

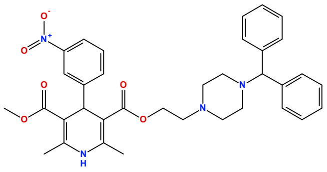

Docking Score: -6.5579

190

DrugBank ID: DB12808

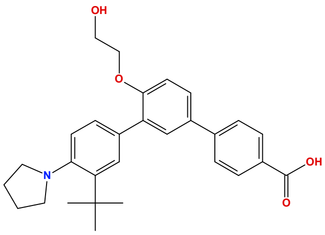

Docking Score: -6.5558

191

DrugBank ID: DB01419

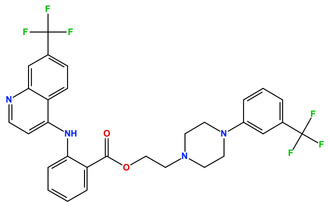

Docking Score: -6.5537

192

DrugBank ID: DB06480

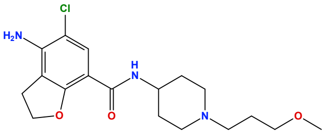

Docking Score: -6.5441

193

DrugBank ID: DB00662

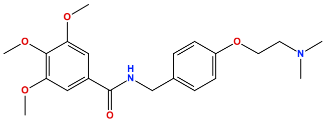

Docking Score: -6.5428

194

DrugBank ID: DB09299

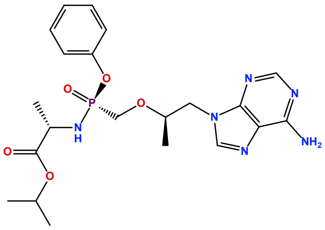

Docking Score: -6.5385

195

DrugBank ID: DB11262

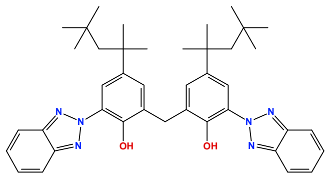

Docking Score: -6.5379

196

DrugBank ID: DB12313

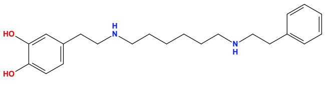

Docking Score: -6.5377

197

DrugBank ID: DB06595

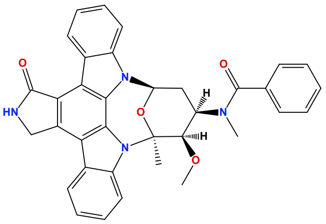

Docking Score: -6.5368

198

DrugBank ID: DB04846

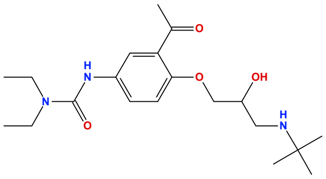

Docking Score: -6.5359

199

DrugBank ID: DB06077

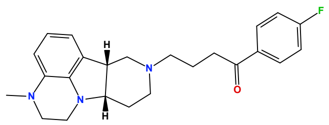

Docking Score: -6.5298

200

DrugBank ID: DB00406

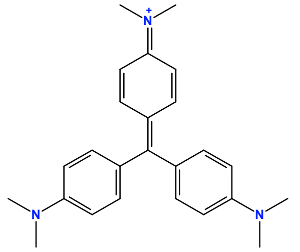

Docking Score: -6.5255

201

DrugBank ID: DB01267

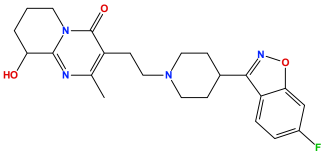

Docking Score: -6.5234

202

DrugBank ID: DB09090

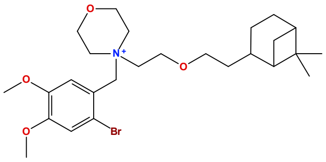

Docking Score: -6.5226

203

DrugBank ID: DB06589

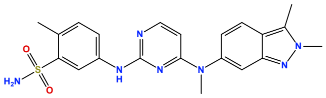

Docking Score: -6.5208

204

DrugBank ID: DB04946

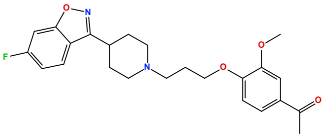

Docking Score: -6.5104

205

DrugBank ID: DB12455

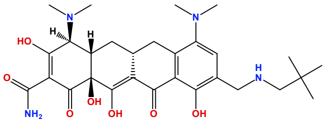

Docking Score: -6.5088

206

DrugBank ID: DB00496

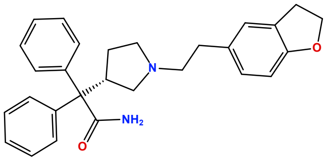

Docking Score: -6.5019

207

DrugBank ID: DB00932

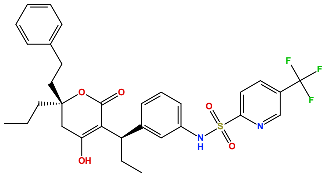

Docking Score: -6.5013

208

DrugBank ID: DB00875

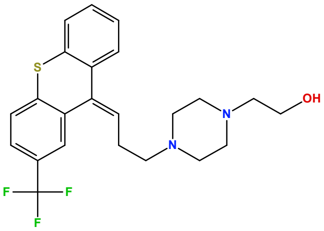

Docking Score: -6.4972

209

DrugBank ID: DB13682

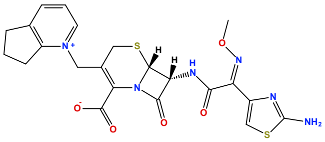

Docking Score: -6.4924

210

DrugBank ID: DB00632

Docking Score: -6.4908

211

DrugBank ID: DB13213

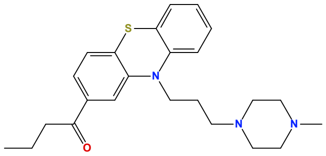

Docking Score: -6.4906

212

DrugBank ID: DB04868

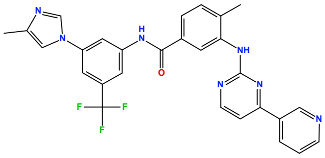

Docking Score: -6.4903

213

DrugBank ID: DB06228

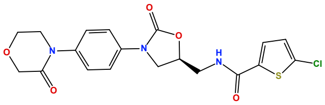

Docking Score: -6.4861

214

DrugBank ID: DB00398

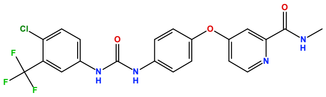

Docking Score: -6.4857

215

DrugBank ID: DB06401

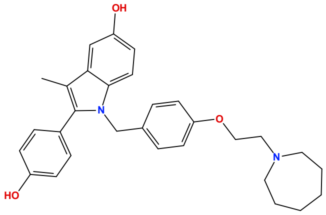

Docking Score: -6.4843

216

DrugBank ID: DB00528

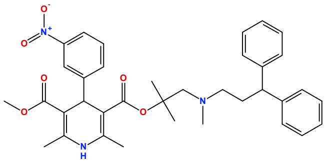

Docking Score: -6.4812

217

DrugBank ID: DB08966

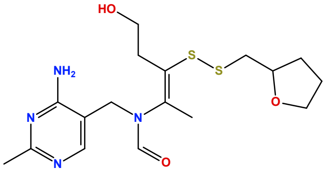

Docking Score: -6.4811

218

DrugBank ID: DB12153

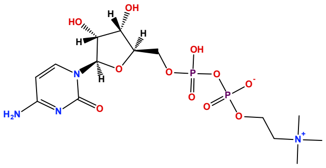

Docking Score: -6.4787

219

DrugBank ID: DB00917

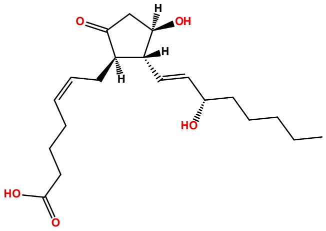

Docking Score: -6.4781

220

DrugBank ID: DB12713

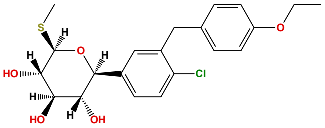

Docking Score: -6.4730

221

DrugBank ID: DB08860

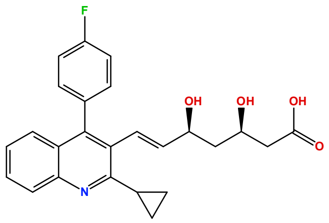

Docking Score: -6.4635

222

DrugBank ID: DB12267

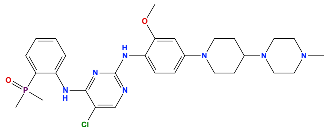

Docking Score: -6.4553

223

DrugBank ID: DB08897

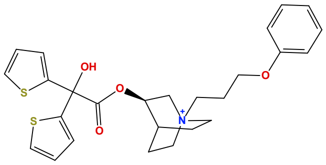

Docking Score: -6.4546

224

DrugBank ID: DB01149

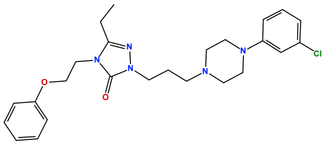

Docking Score: -6.4522

225

DrugBank ID: DB06292

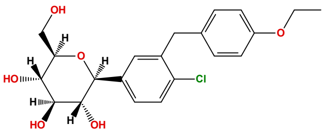

Docking Score: -6.4439

226

DrugBank ID: DB09083

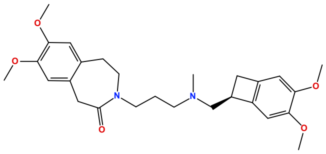

Docking Score: -6.4352

227

DrugBank ID: DB03310

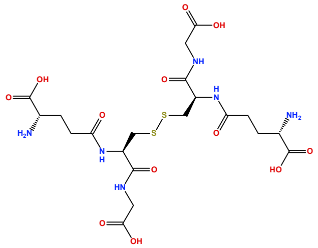

Docking Score: -6.4313

228

DrugBank ID: DB11691

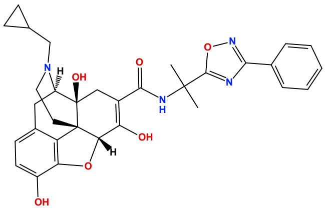

Docking Score: -6.4294

229

DrugBank ID: DB00445

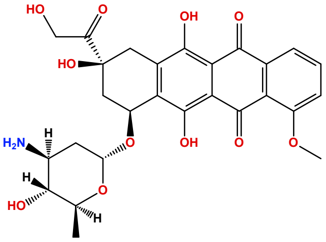

Docking Score: -6.4281

230

DrugBank ID: DB12483

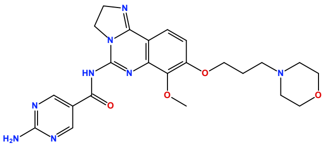

Docking Score: -6.4226

231

DrugBank ID: DB09319

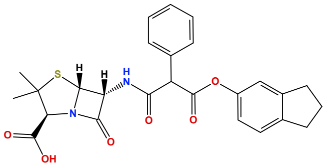

Docking Score: -6.4147

232

DrugBank ID: DB14725

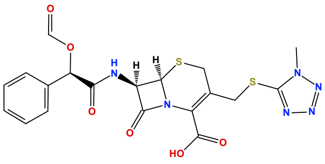

Docking Score: -6.4075

233

DrugBank ID: DB12710

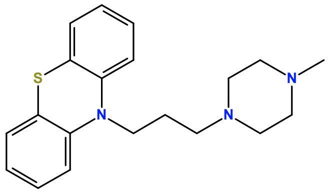

Docking Score: -6.4065

234

DrugBank ID: DB13166

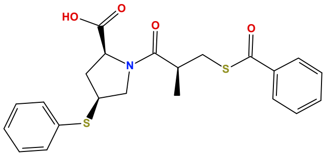

Docking Score: -6.4042

235

DrugBank ID: DB06827

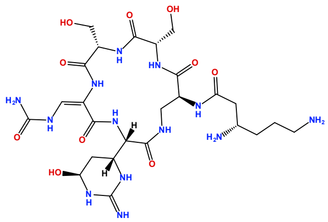

Docking Score: -6.4030

236

DrugBank ID: DB13074

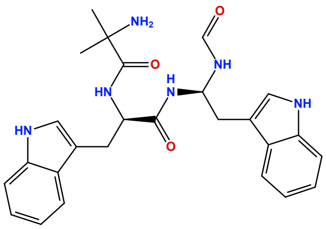

Docking Score: -6.4028

237

DrugBank ID: DB08864

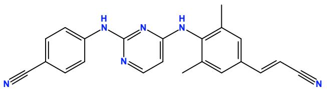

Docking Score: -6.4026

238

DrugBank ID: DB06448

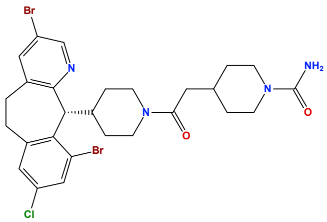

Docking Score: -6.4009

239

DrugBank ID: DB00267

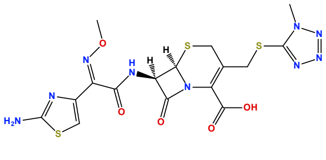

Docking Score: -6.3994

240

DrugBank ID: DB08827

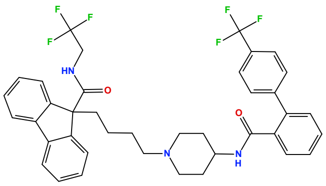

Docking Score: -6.3991

241

DrugBank ID: DB09135

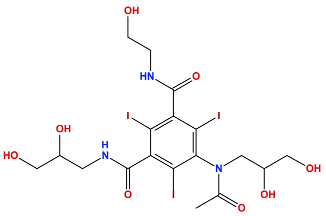

Docking Score: -6.3982

242

DrugBank ID: DB09148

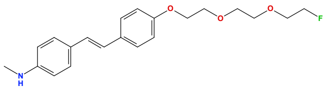

Docking Score: -6.3923

243

DrugBank ID: DB11730

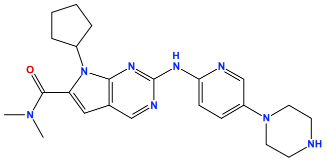

Docking Score: -6.3916

244

DrugBank ID: DB00585

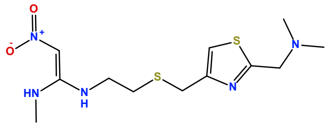

Docking Score: -6.3879

245

DrugBank ID: DB00661

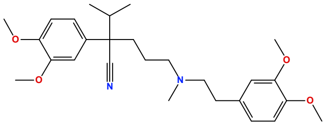

Docking Score: -6.3848

246

DrugBank ID: DB11630

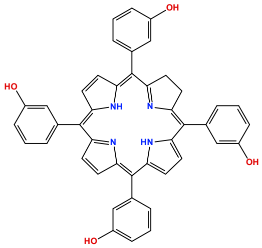

Docking Score: -6.3844

247

DrugBank ID: DB01338

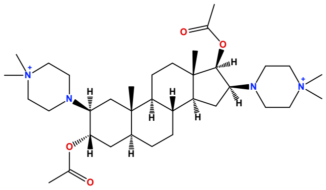

Docking Score: -6.3816

248

DrugBank ID: DB01327

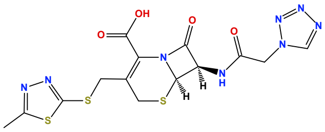

Docking Score: -6.3807

249

DrugBank ID: DB15444

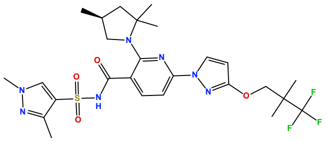

Docking Score: -6.3802

250

DrugBank ID: DB01026

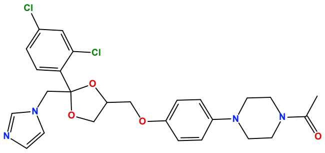

Docking Score: -6.3800

251

DrugBank ID: DB01251

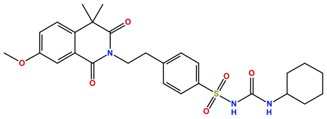

Docking Score: -6.3782

252

DrugBank ID: DB00251

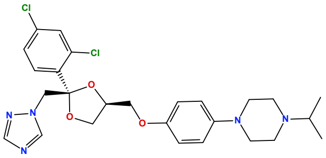

Docking Score: -6.3777

253

DrugBank ID: DB09280

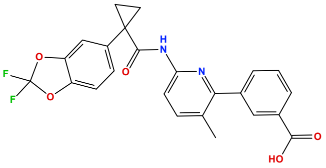

Docking Score: -6.3758

254

DrugBank ID: DB11672

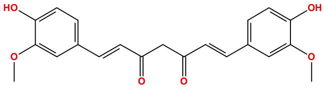

Docking Score: -6.3738

255

DrugBank ID: DB00656

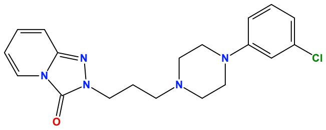

Docking Score: -6.3735

256

DrugBank ID: DB00686

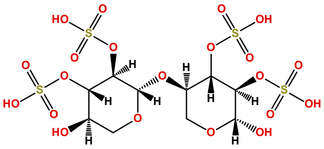

Docking Score: -6.3731

257

DrugBank ID: DB09534

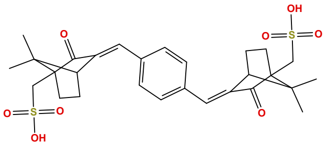

Docking Score: -6.3711

258

DrugBank ID: DB00938

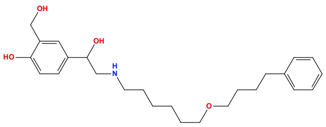

Docking Score: -6.3711

259

DrugBank ID: DB01204

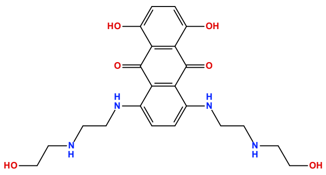

Docking Score: -6.3690

260

DrugBank ID: DB06016

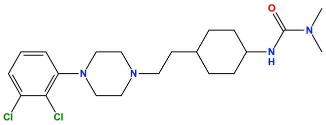

Docking Score: -6.3665

261

DrugBank ID: DB13874

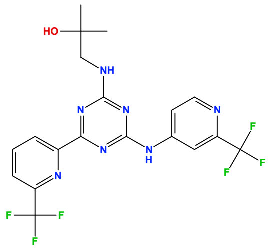

Docking Score: -6.3653

262

DrugBank ID: DB01139

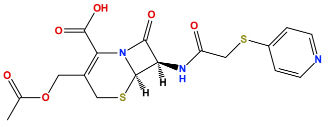

Docking Score: -6.3609

263

DrugBank ID: DB01072

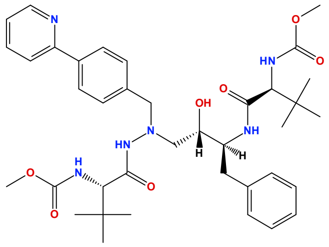

Docking Score: -6.3601

264

DrugBank ID: DB13943

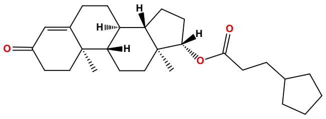

Docking Score: -6.3590

265

DrugBank ID: DB00372

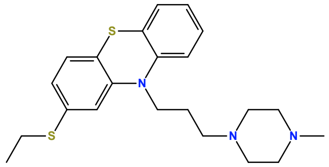

Docking Score: -6.3545

266

DrugBank ID: DB00612

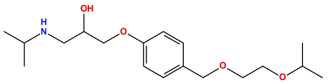

Docking Score: -6.3515

267

DrugBank ID: DB06202

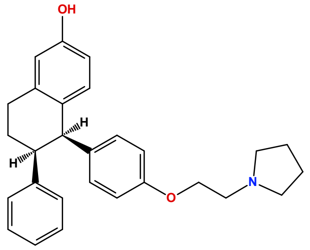

Docking Score: -6.3492

268

DrugBank ID: DB00530

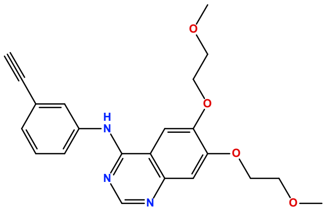

Docking Score: -6.3485

269

DrugBank ID: DB00482

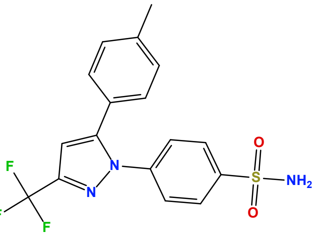

Docking Score: -6.3431

270

DrugBank ID: DB01228

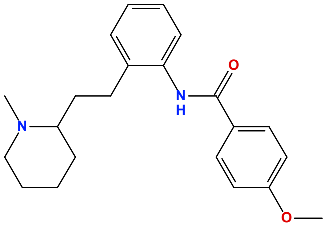

Docking Score: -6.3369

271

DrugBank ID: DB00177

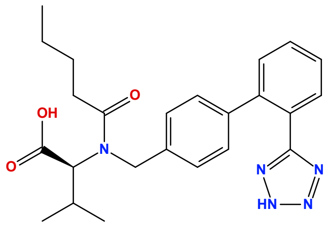

Docking Score: -6.3355

272

DrugBank ID: DB14476

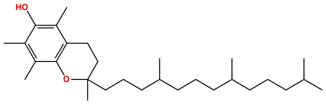

Docking Score: -6.3345

273

DrugBank ID: DB00354

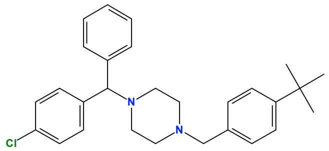

Docking Score: -6.3326

274

DrugBank ID: DB04865

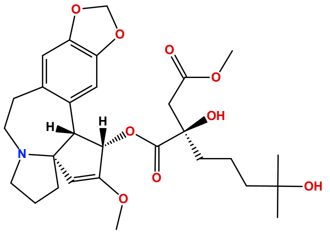

Docking Score: -6.3285

275

DrugBank ID: DB08887

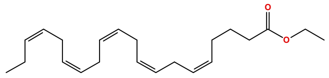

Docking Score: -6.3271

276

DrugBank ID: DB11184

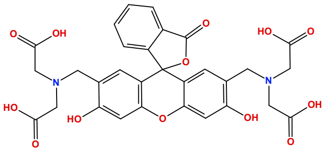

Docking Score: -6.3259

277

DrugBank ID: DB00471

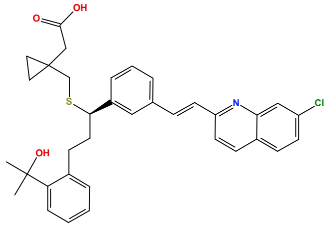

Docking Score: -6.3233

278

DrugBank ID: DB14541

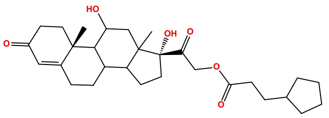

Docking Score: -6.3215

279

DrugBank ID: DB01268

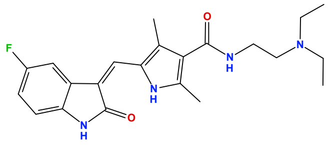

Docking Score: -6.3192

280

DrugBank ID: DB01605

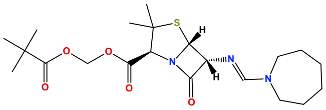

Docking Score: -6.3126

281

DrugBank ID: DB11269

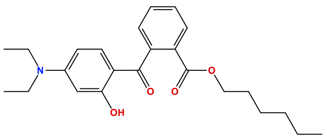

Docking Score: -6.3069

282

DrugBank ID: DB13142

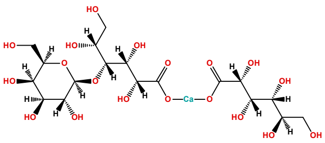

Docking Score: -6.3061

283

DrugBank ID: DB08875

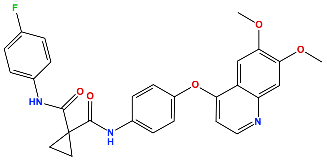

Docking Score: -6.3020

284

DrugBank ID: DB00675

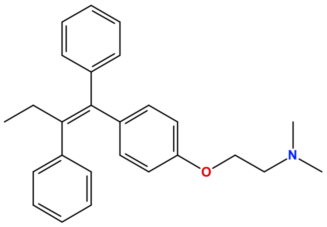

Docking Score: -6.2983

285

DrugBank ID: DB09074

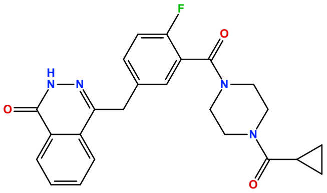

Docking Score: -6.2980

286

DrugBank ID: DB13258

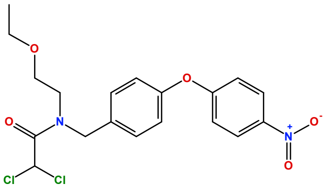

Docking Score: -6.2945

287

DrugBank ID: DB08906

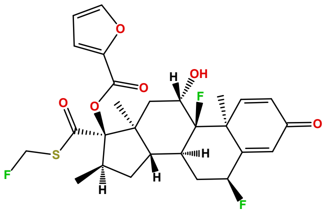

Docking Score: -6.2935

288

DrugBank ID: DB15305

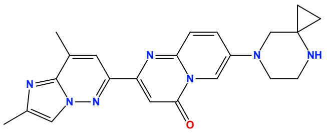

Docking Score: -6.2933

289

DrugBank ID: DB08867

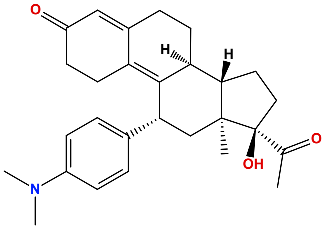

Docking Score: -6.2817

290

DrugBank ID: DB11689

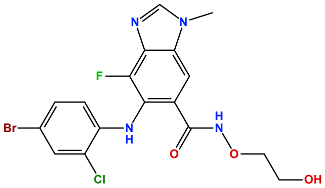

Docking Score: -6.2806

291

DrugBank ID: DB00519

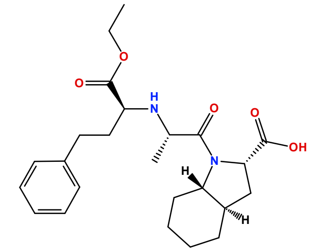

Docking Score: -6.2802

292

DrugBank ID: DB12147

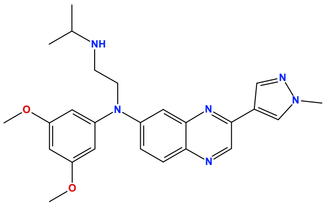

Docking Score: -6.2797

293

DrugBank ID: DB00798

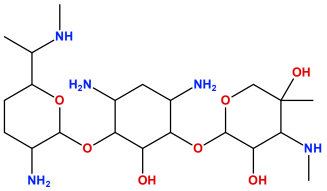

Docking Score: -6.2750

294

DrugBank ID: DB01101

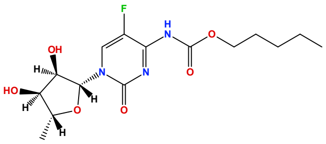

Docking Score: -6.2705

295

DrugBank ID: DB01621

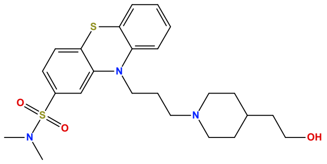

Docking Score: -6.2691

296

DrugBank ID: DB04918

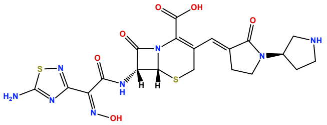

Docking Score: -6.2644

297

DrugBank ID: DB00929

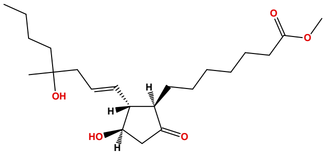

Docking Score: -6.2542

298

DrugBank ID: DB00220

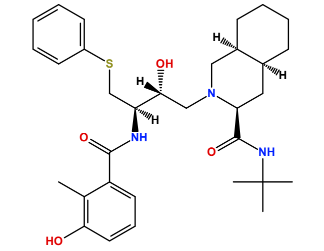

Docking Score: -6.2537

299

DrugBank ID: DB09136

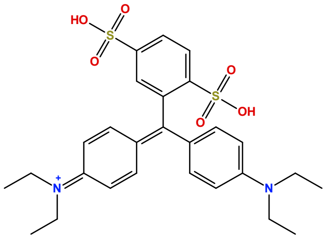

Docking Score: -6.2522

300

DrugBank ID: DB00670

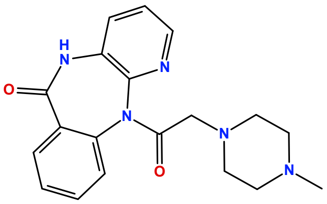

Docking Score: -6.2520

301

DrugBank ID: DB01003

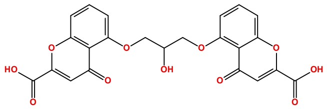

Docking Score: -6.2495

302

DrugBank ID: DB00557

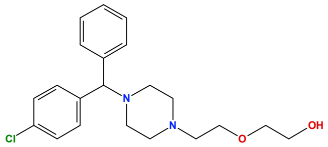

Docking Score: -6.2476

303

DrugBank ID: DB09350

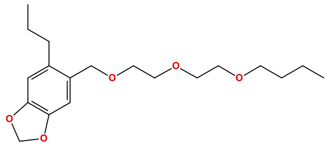

Docking Score: -6.2452

304

DrugBank ID: DB00623

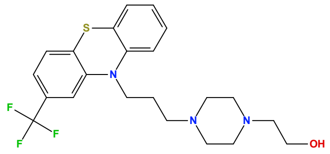

Docking Score: -6.2445

305

DrugBank ID: DB12015

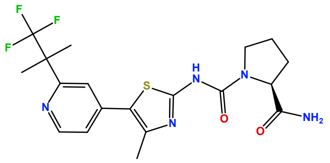

Docking Score: -6.2430

306

DrugBank ID: DB00984

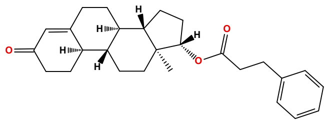

Docking Score: -6.2375

307

DrugBank ID: DB12364

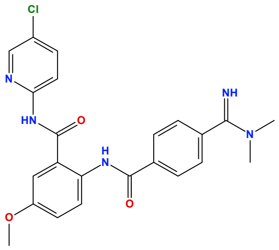

Docking Score: -6.2354

308

DrugBank ID: DB12371

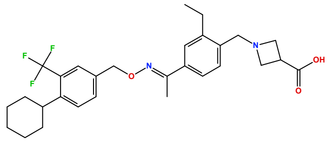

Docking Score: -6.2338

309

DrugBank ID: DB09020

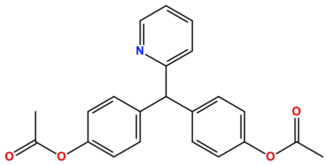

Docking Score: -6.2334

310

DrugBank ID: DB11791

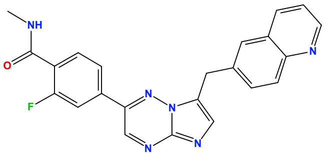

Docking Score: -6.2329

311

DrugBank ID: DB14007

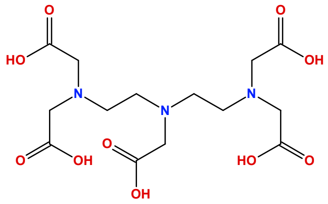

Docking Score: -6.2301

312

DrugBank ID: DB14840

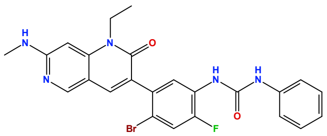

Docking Score: -6.2281

313

DrugBank ID: DB08439

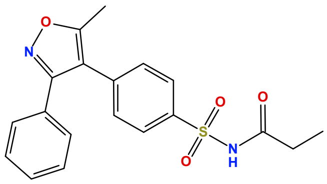

Docking Score: -6.2279

314

DrugBank ID: DB00562

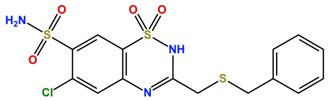

Docking Score: -6.2275

315

DrugBank ID: DB00831

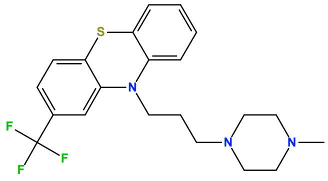

Docking Score: -6.2244

316

DrugBank ID: DB06274

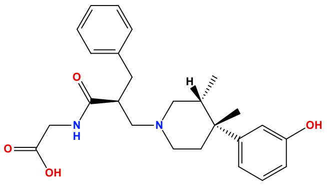

Docking Score: -6.2212

317

DrugBank ID: DB00691

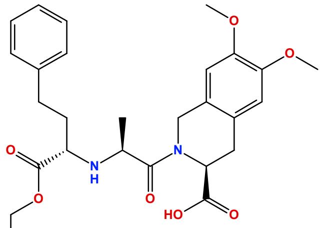

Docking Score: -6.2209

318

DrugBank ID: DB00722

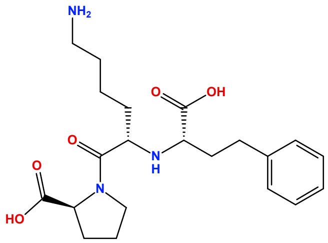

Docking Score: -6.2195

319

DrugBank ID: DB14082

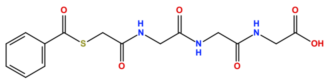

Docking Score: -6.2178

320

DrugBank ID: DB00769

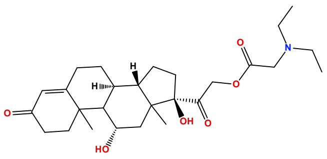

Docking Score: -6.2167

321

DrugBank ID: DB00175

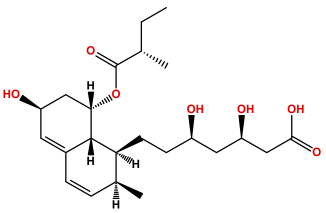

Docking Score: -6.2163

322

DrugBank ID: DB04930

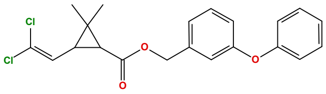

Docking Score: -6.2153

323

DrugBank ID: DB00374

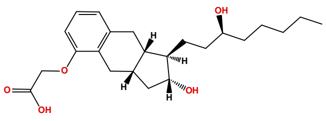

Docking Score: -6.2137

324

DrugBank ID: DB08882

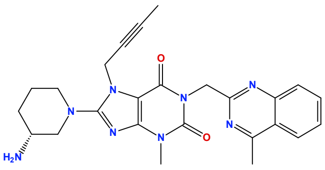

Docking Score: -6.2135

325

DrugBank ID: DB11226

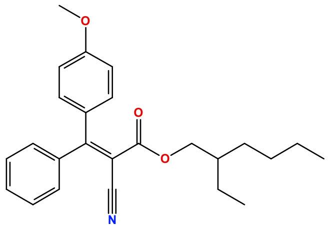

Docking Score: -6.2114

326

DrugBank ID: DB00843

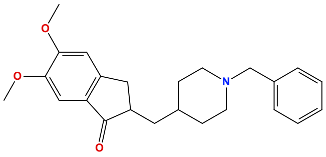

Docking Score: -6.2104

327

DrugBank ID: DB05676

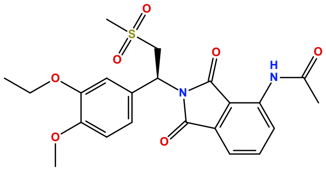

Docking Score: -6.2074

328

DrugBank ID: DB15617

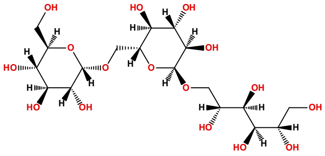

Docking Score: -6.2022

329

DrugBank ID: DB12941

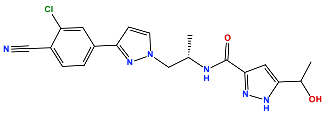

Docking Score: -6.1988

330

DrugBank ID: DB09183

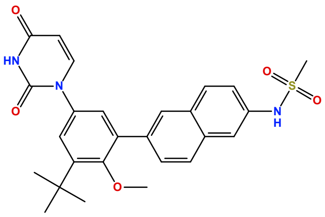

Docking Score: -6.1925

331

DrugBank ID: DB13766

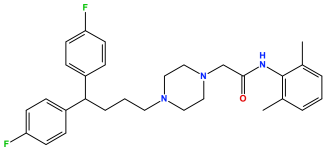

Docking Score: -6.1908

332

DrugBank ID: DB00159

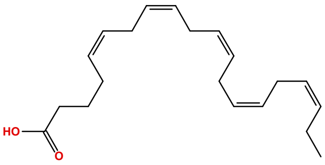

Docking Score: -6.1868

333

DrugBank ID: DB00303

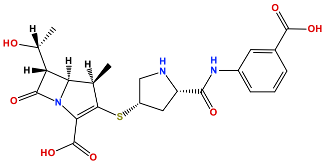

Docking Score: -6.1861

334

DrugBank ID: DB13520

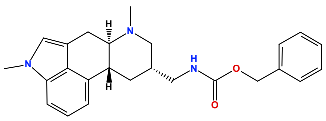

Docking Score: -6.1799

335

DrugBank ID: DB01331

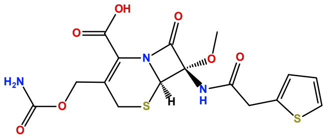

Docking Score: -6.1776

336

DrugBank ID: DB06605

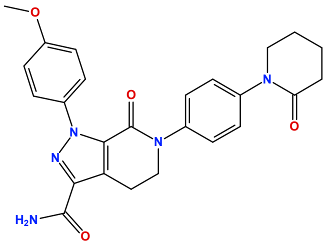

Docking Score: -6.1746

337

DrugBank ID: DB01193

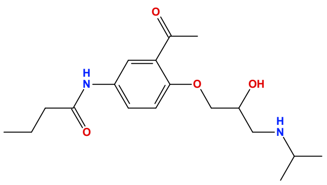

Docking Score: -6.1735

338

DrugBank ID: DB00590

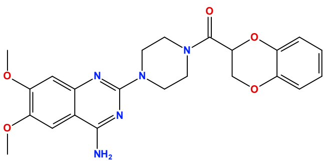

Docking Score: -6.1721

339

DrugBank ID: DB00604

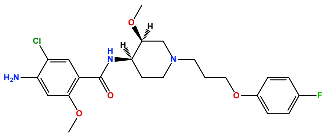

Docking Score: -6.1693

340

DrugBank ID: DB09120

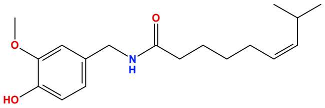

Docking Score: -6.1679

341

DrugBank ID: DB11753

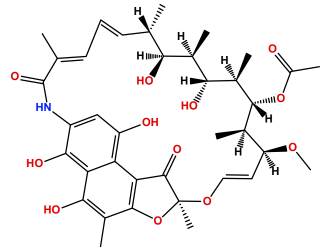

Docking Score: -6.1677

342

DrugBank ID: DB06288

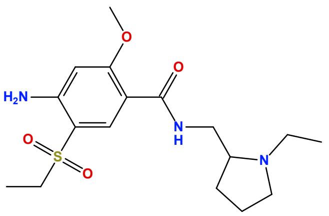

Docking Score: -6.1664

343

DrugBank ID: DB01220

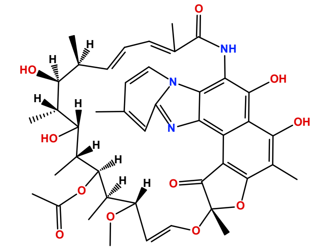

Docking Score: -6.1659

344

DrugBank ID: DB00706

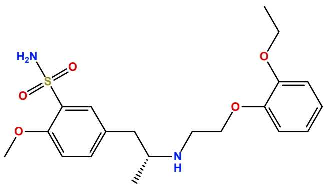

Docking Score: -6.1563

345

DrugBank ID: DB01166

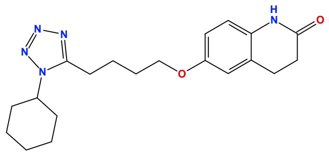

Docking Score: -6.1471

346

DrugBank ID: DB00248

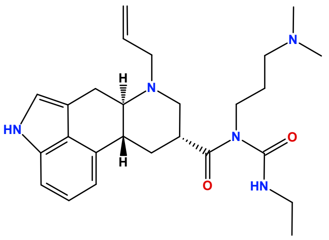

Docking Score: -6.1417

347

DrugBank ID: DB00412

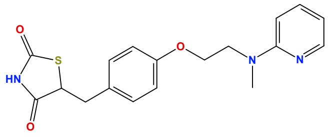

Docking Score: -6.1408

348

DrugBank ID: DB01187

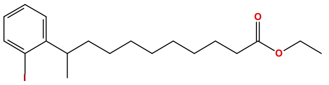

Docking Score: -6.1406

349

DrugBank ID: DB11951

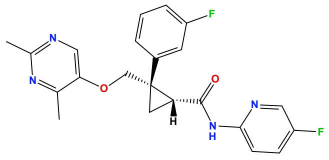

Docking Score: -6.1401

350

DrugBank ID: DB08868

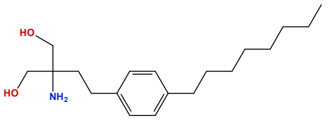

Docking Score: -6.1392

351

DrugBank ID: DB09048

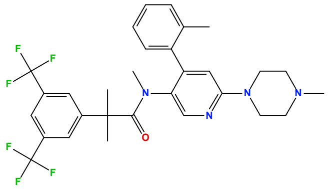

Docking Score: -6.1380

352

DrugBank ID: DB14674

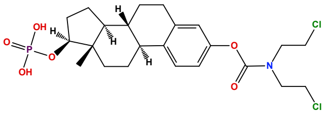

Docking Score: -6.1346

353

DrugBank ID: DB00845

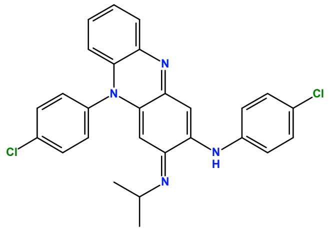

Docking Score: -6.1309

354

DrugBank ID: DB13337

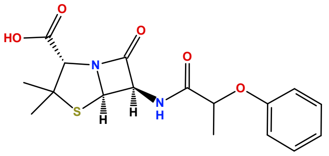

Docking Score: -6.1268

355

DrugBank ID: DB11614

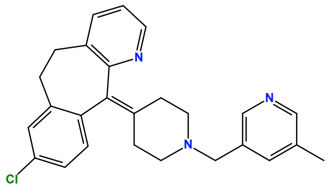

Docking Score: -6.1262

356

DrugBank ID: DB11963

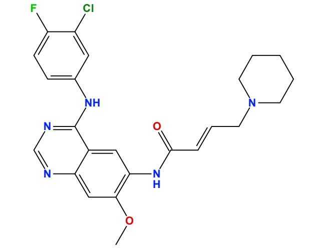

Docking Score: -6.1241

357

DrugBank ID: DB09292

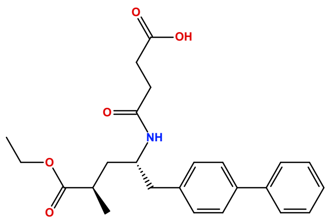

Docking Score: -6.1199

358

DrugBank ID: DB01127

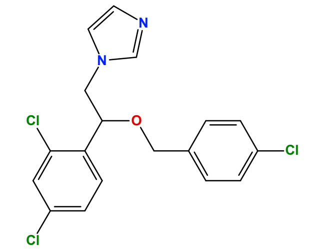

Docking Score: -6.1185

359

DrugBank ID: DB00216

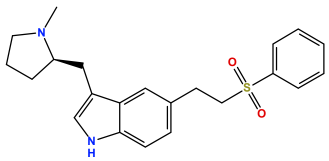

Docking Score: -6.1164

360

DrugBank ID: DB03808

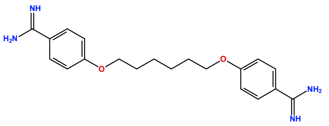

Docking Score: -6.1126

361

DrugBank ID: DB12457

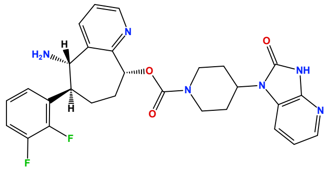

Docking Score: -6.1103

362

DrugBank ID: DB13858

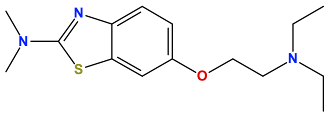

Docking Score: -6.1006

363

DrugBank ID: DB01088

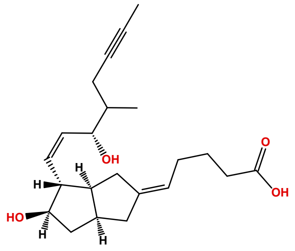

Docking Score: -6.0957

364

DrugBank ID: DB06414

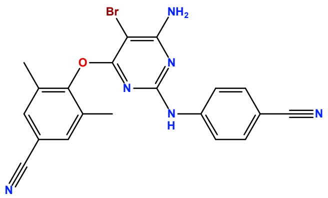

Docking Score: -6.0941

365

DrugBank ID: DB08815

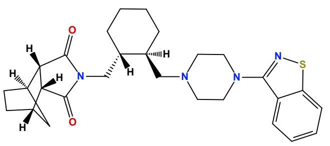

Docking Score: -6.0922

366

DrugBank ID: DB09084

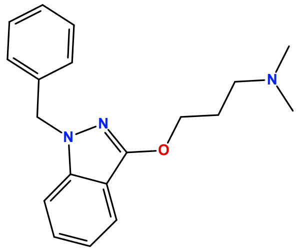

Docking Score: -6.0892

367

DrugBank ID: DB12612

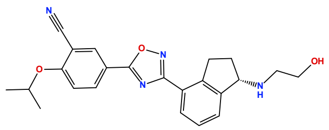

Docking Score: -6.0890

368

DrugBank ID: DB11827

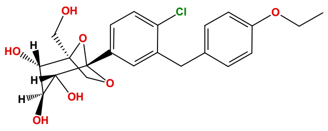

Docking Score: -6.0875

369

DrugBank ID: DB01184

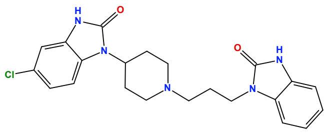

Docking Score: -6.0810

370

DrugBank ID: DB11921

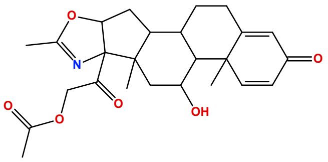

Docking Score: -6.0805

371

DrugBank ID: DB00187

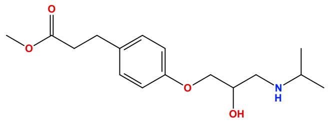

Docking Score: -6.0780

372

DrugBank ID: DB01254

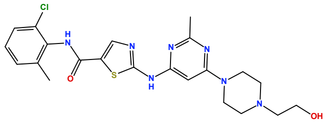

Docking Score: -6.0769

373

DrugBank ID: DB12278

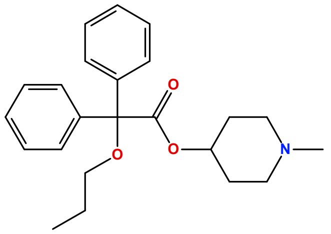

Docking Score: -6.0759

374

DrugBank ID: DB13157

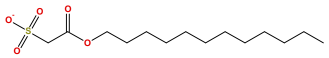

Docking Score: -6.0741

375

DrugBank ID: DB01199

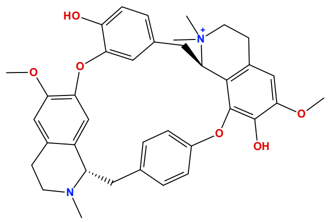

Docking Score: -6.0731

376

DrugBank ID: DB00342

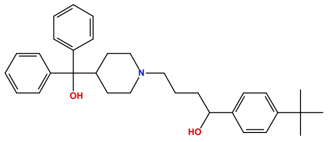

Docking Score: -6.0712

377

DrugBank ID: DB00737

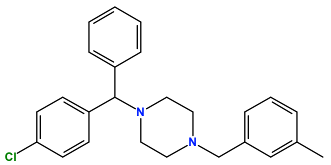

Docking Score: -6.0692

378

DrugBank ID: DB14631

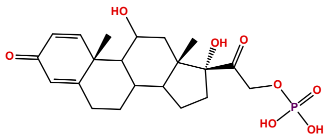

Docking Score: -6.0630

379

DrugBank ID: DB00197

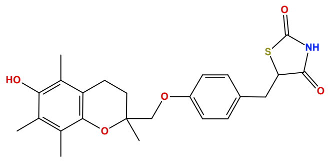

Docking Score: -6.0608

380

DrugBank ID: DB01157

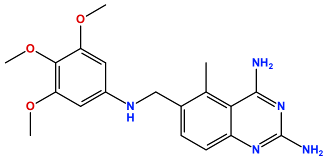

Docking Score: -6.0605

381

DrugBank ID: DB12377

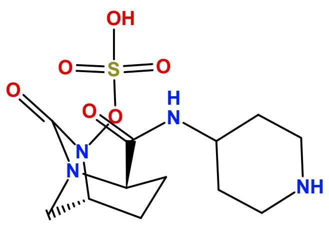

Docking Score: -6.0566

382

DrugBank ID: DB13783

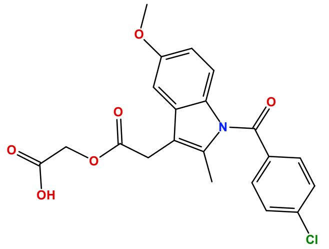

Docking Score: -6.0526

383

DrugBank ID: DB00743

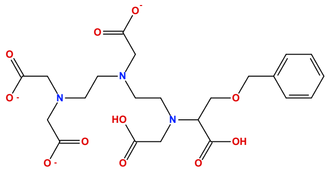

Docking Score: -6.0490

384

DrugBank ID: DB01599

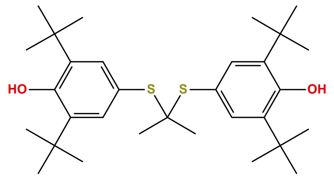

Docking Score: -6.0473

385

DrugBank ID: DB15102

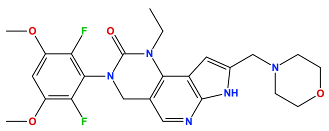

Docking Score: -6.0423

386

DrugBank ID: DB00401

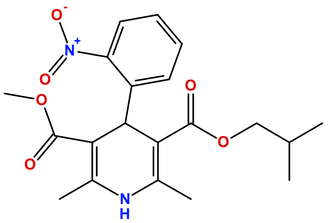

Docking Score: -6.0421

387

DrugBank ID: DB01348

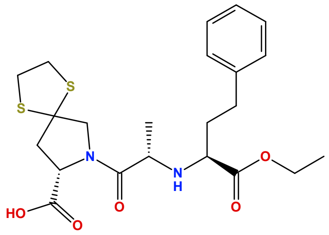

Docking Score: -6.0407

388

DrugBank ID: DB01256

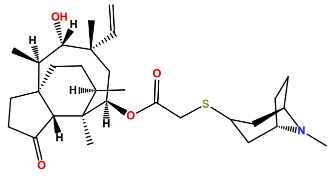

Docking Score: -6.0402

389

DrugBank ID: DB05239

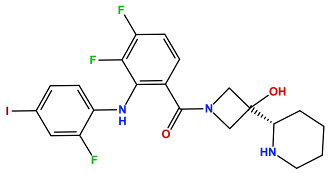

Docking Score: -6.0392

390

DrugBank ID: DB13781

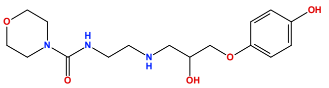

Docking Score: -6.0389

391

DrugBank ID: DB11748

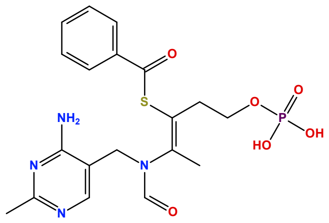

Docking Score: -6.0317

392

DrugBank ID: DB14540

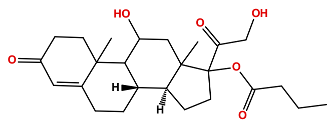

Docking Score: -6.0276

393

DrugBank ID: DB05273

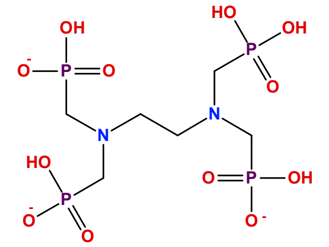

Docking Score: -6.0273

394

DrugBank ID: DB14568

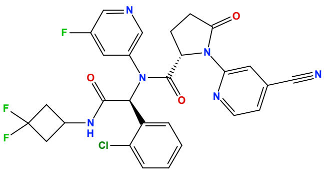

Docking Score: -6.0267

395

DrugBank ID: DB01195

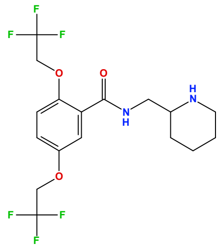

Docking Score: -6.0241

396

DrugBank ID: DB00209

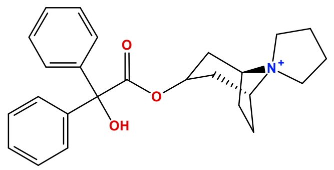

Docking Score: -6.0192

397

DrugBank ID: DB01610

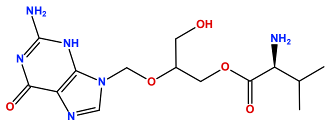

Docking Score: -6.0188

398

DrugBank ID: DB01609

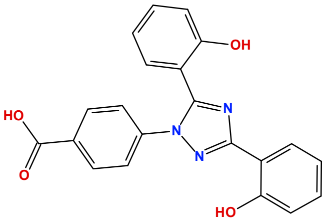

Docking Score: -6.0186

399

DrugBank ID: DB01627

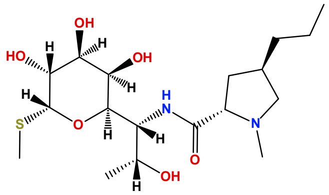

Docking Score: -6.0171

400

DrugBank ID: DB01198

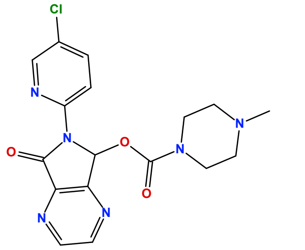

Docking Score: -6.0144

401

DrugBank ID: DB00118

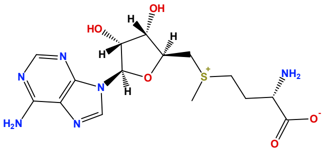

Docking Score: -6.0139

402

DrugBank ID: DB00863

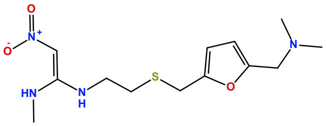

Docking Score: -6.0135

403

DrugBank ID: DB01136

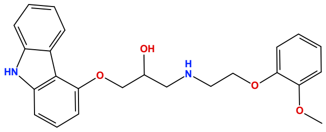

Docking Score: -6.0107

404

DrugBank ID: DB01182

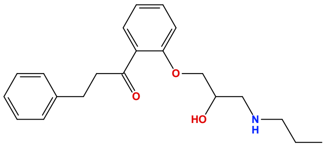

Docking Score: -6.0080

405

DrugBank ID: DB05316

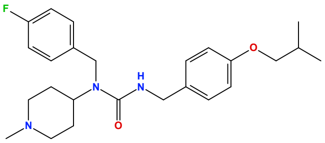

Docking Score: -6.0077

406

DrugBank ID: DB09195

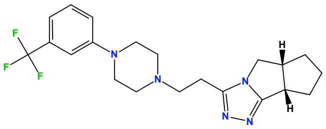

Docking Score: -6.0072

407

DrugBank ID: DB06210

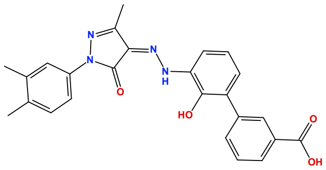

Docking Score: -6.0072

408

DrugBank ID: DB08931

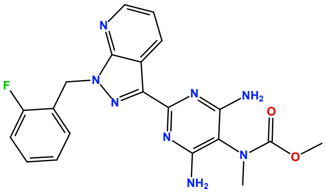

Docking Score: -6.0061

409

DrugBank ID: DB01393

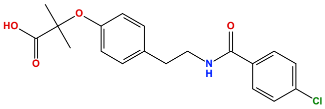

Docking Score: -6.0060

410

DrugBank ID: DB08911

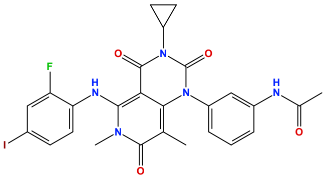

Docking Score: -6.0049

411

DrugBank ID: DB00645

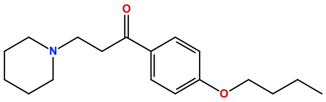

Docking Score: -6.0047

412

DrugBank ID: DB00425

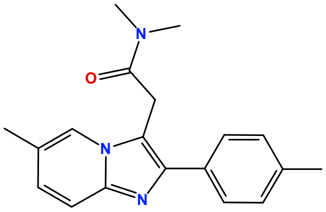

Docking Score: -6.0007

413

DrugBank ID: DB09144

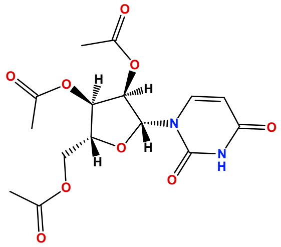

Docking Score: -5.9969

414

DrugBank ID: DB06268

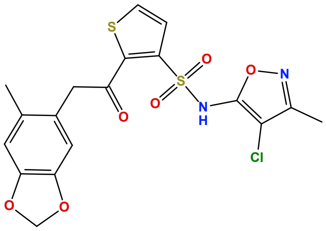

Docking Score: -5.9960

415

DrugBank ID: DB01162

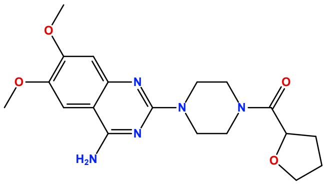

Docking Score: -5.9940

416

DrugBank ID: DB01987

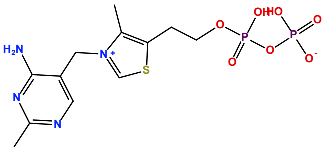

Docking Score: -5.9914

417

DrugBank ID: DB01046

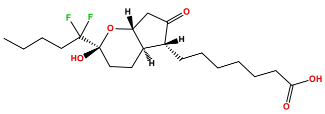

Docking Score: -5.9866

418

DrugBank ID: DB06786

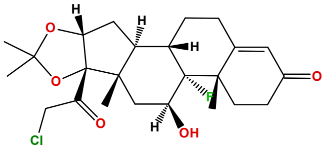

Docking Score: -5.9857

419

DrugBank ID: DB14596

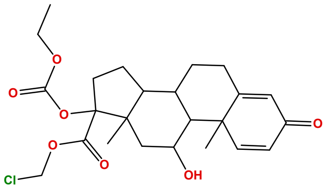

Docking Score: -5.9840

420

DrugBank ID: DB07565

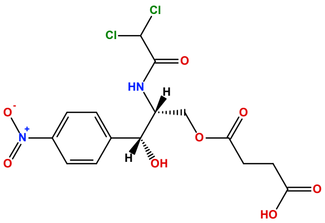

Docking Score: -5.9790

421

DrugBank ID: DB01014

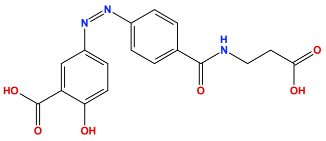

Docking Score: -5.9766

422

DrugBank ID: DB11363

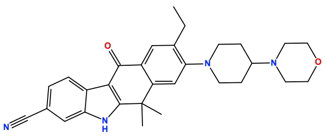

Docking Score: -5.9761

423

DrugBank ID: DB06708

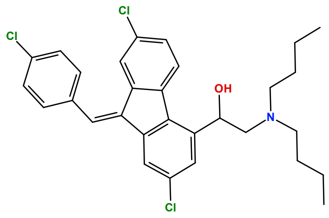

Docking Score: -5.9745

424

DrugBank ID: DB02300

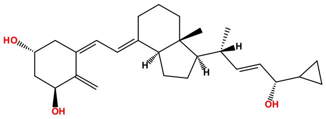

Docking Score: -5.9708

425

DrugBank ID: DB06209

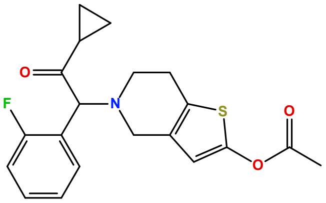

Docking Score: -5.9701

426

DrugBank ID: DB12978

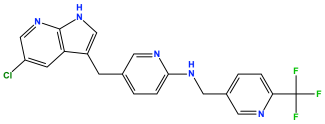

Docking Score: -5.9701

427

DrugBank ID: DB01274

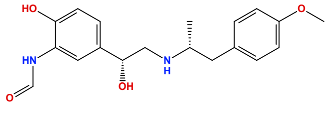

Docking Score: -5.9673

428

DrugBank ID: DB01092

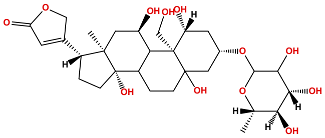

Docking Score: -5.9654

429

DrugBank ID: DB00450

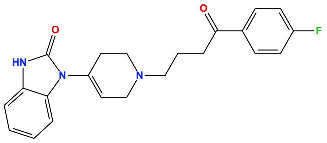

Docking Score: -5.9633

430

DrugBank ID: DB00820

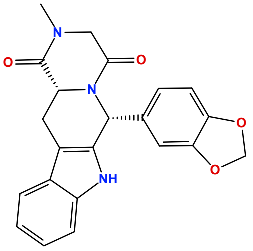

Docking Score: -5.9614

431

DrugBank ID: DB08912

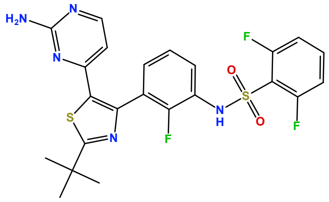

Docking Score: -5.9583

432

DrugBank ID: DB00705

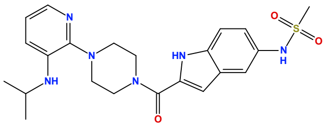

Docking Score: -5.9583

433

DrugBank ID: DB09073

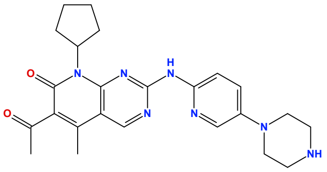

Docking Score: -5.9538

434

DrugBank ID: DB09053

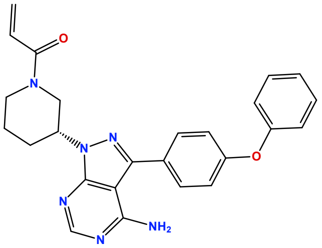

Docking Score: -5.9538

435

DrugBank ID: DB04575

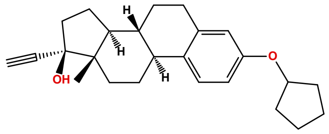

Docking Score: -5.9536

436

DrugBank ID: DB00799

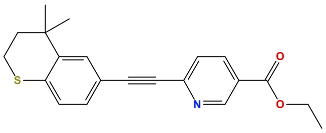

Docking Score: -5.9530

437

DrugBank ID: DB00254

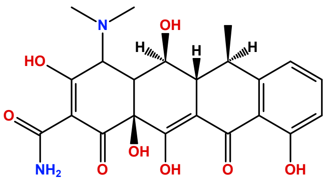

Docking Score: -5.9518

438

DrugBank ID: DB12867

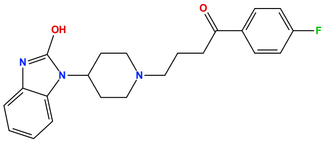

Docking Score: -5.9517

439

DrugBank ID: DB01147

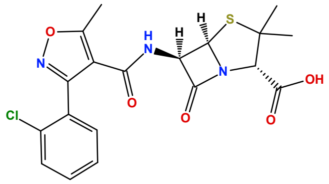

Docking Score: -5.9502

440

DrugBank ID: DB01214

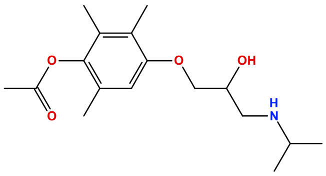

Docking Score: -5.9486

441

DrugBank ID: DB09291

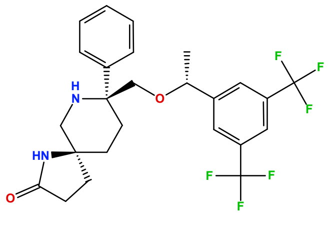

Docking Score: -5.9475

442

DrugBank ID: DB11979

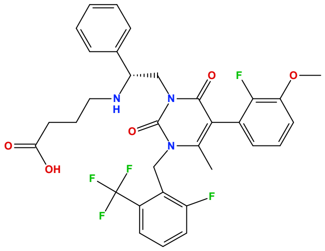

Docking Score: -5.9440

443

DrugBank ID: DB06237

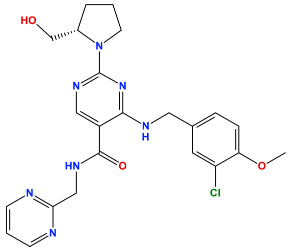

Docking Score: -5.9434

444

DrugBank ID: DB09047

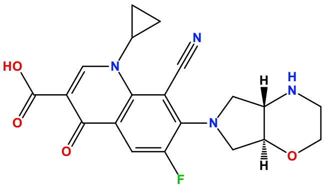

Docking Score: -5.9433

445

DrugBank ID: DB09351

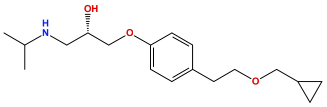

Docking Score: -5.9423

446

DrugBank ID: DB05351

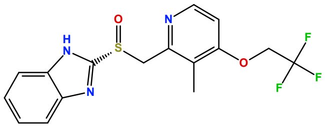

Docking Score: -5.9421

447

DrugBank ID: DB01597

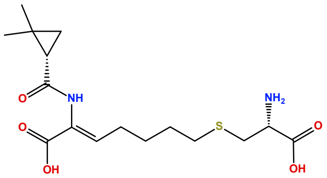

Docking Score: -5.9389

448

DrugBank ID: DB11799

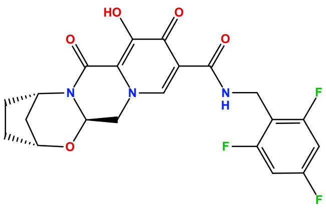

Docking Score: -5.9372

449

DrugBank ID: DB00246

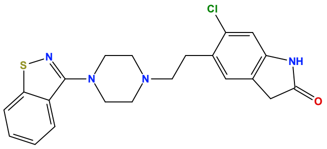

Docking Score: -5.9365

450

DrugBank ID: DB00542

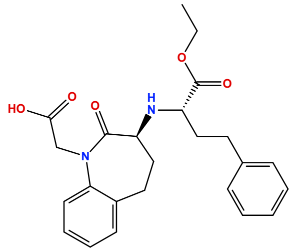

Docking Score: -5.9363

451

DrugBank ID: DB08820

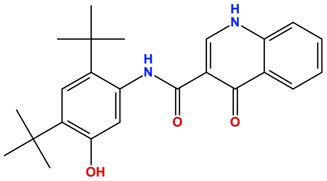

Docking Score: -5.9307

452

DrugBank ID: DB01129

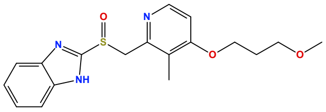

Docking Score: -5.9278

453

DrugBank ID: DB03247

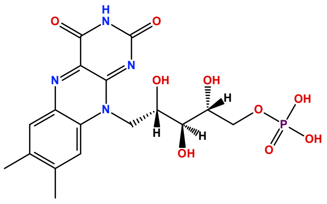

Docking Score: -5.9237

454

DrugBank ID: DB11677

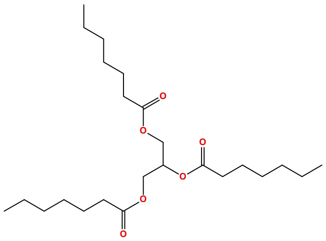

Docking Score: -5.9184

455

DrugBank ID: DB06608

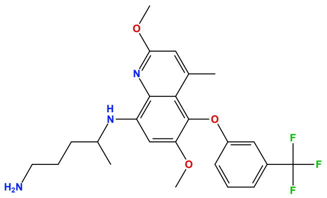

Docking Score: -5.9128

456

DrugBank ID: DB01410

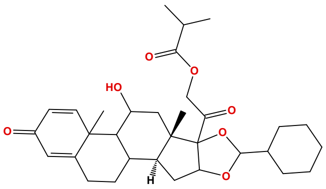

Docking Score: -5.9100

457

DrugBank ID: DB01063

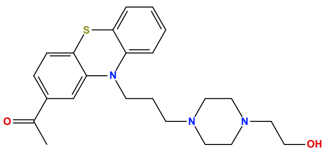

Docking Score: -5.9091

458

DrugBank ID: DB00699

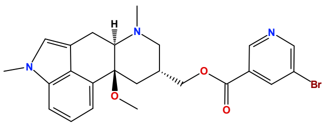

Docking Score: -5.9065

459

DrugBank ID: DB00188

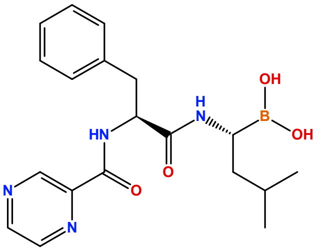

Docking Score: -5.9045

460

DrugBank ID: DB08865

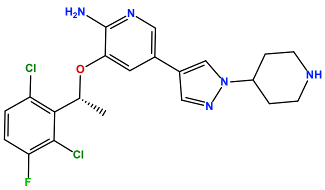

Docking Score: -5.9043

461

DrugBank ID: DB00373

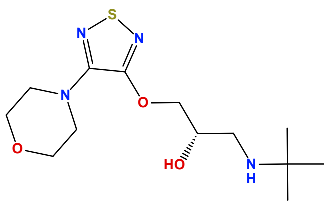

Docking Score: -5.9001

462

DrugBank ID: DB11978

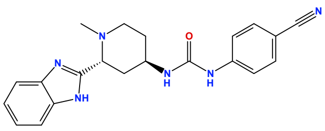

Docking Score: -5.8978

463

DrugBank ID: DB08916

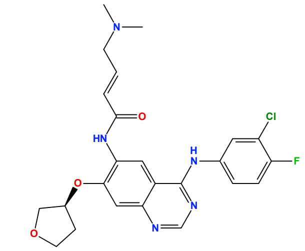

Docking Score: -5.8975

464

DrugBank ID: DB01061

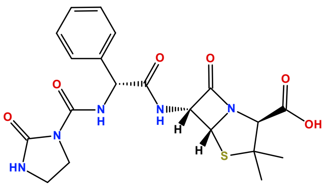

Docking Score: -5.8974

465

DrugBank ID: DB00679

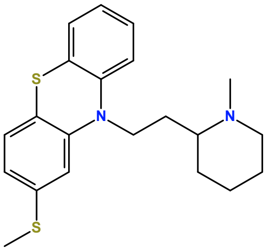

Docking Score: -5.8963

466

DrugBank ID: DB11817

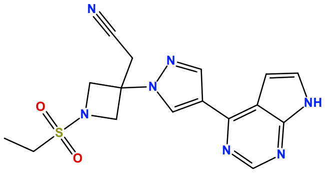

Docking Score: -5.8949

467

DrugBank ID: DB01224

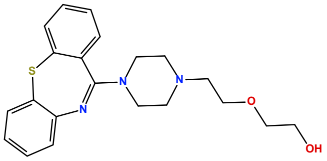

Docking Score: -5.8920

468

DrugBank ID: DB01066

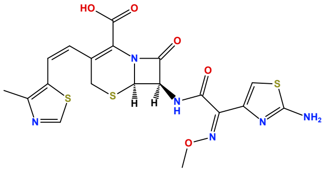

Docking Score: -5.8899

469

DrugBank ID: DB06780

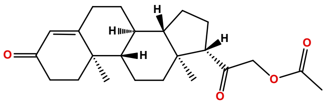

Docking Score: -5.8873

470

DrugBank ID: DB11591

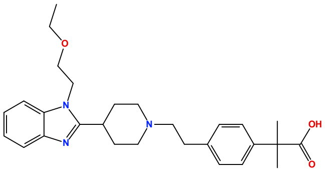

Docking Score: -5.8872

471

DrugBank ID: DB06144

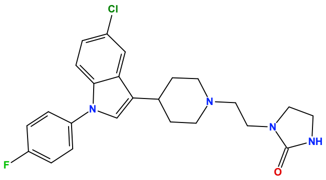

Docking Score: -5.8848

472

DrugBank ID: DB00950

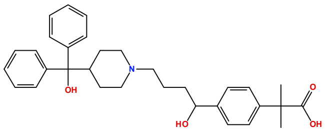

Docking Score: -5.8841

473

DrugBank ID: DB01632

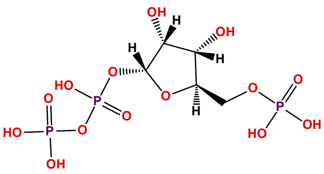

Docking Score: -5.8833

474

DrugBank ID: DB09101

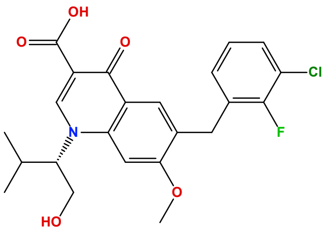

Docking Score: -5.8824

475

DrugBank ID: DB01413

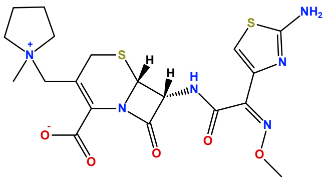

Docking Score: -5.8814

476

DrugBank ID: DB08941

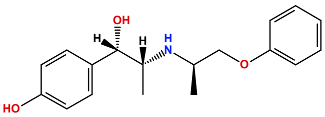

Docking Score: -5.8814

477

DrugBank ID: DB05294

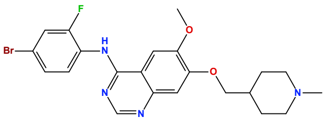

Docking Score: -5.8810

478

DrugBank ID: DB08903

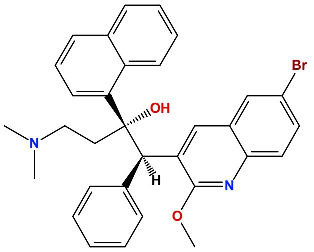

Docking Score: -5.8805

479

DrugBank ID: DB00535

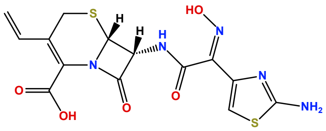

Docking Score: -5.8802

480

DrugBank ID: DB00689

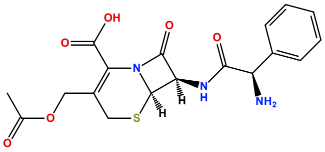

Docking Score: -5.8763

481

DrugBank ID: DB00275

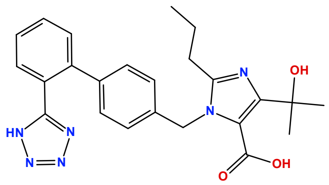

Docking Score: -5.8743

482

DrugBank ID: DB08893

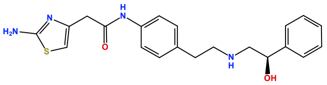

Docking Score: -5.8683

483

DrugBank ID: DB00927

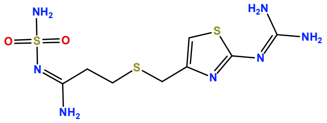

Docking Score: -5.8674

484

DrugBank ID: DB04834

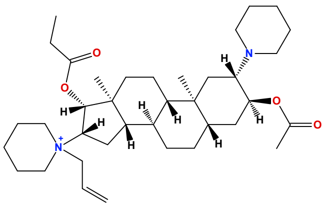

Docking Score: -5.8670

485

DrugBank ID: DB12130

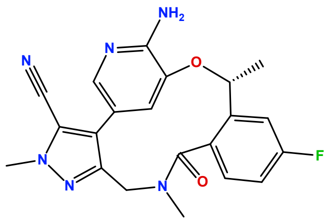

Docking Score: -5.8623

486

DrugBank ID: DB01591

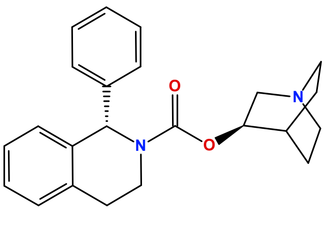

Docking Score: -5.8604

487

DrugBank ID: DB01076

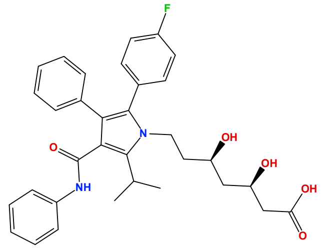

Docking Score: -5.8604

488

DrugBank ID: DB00637

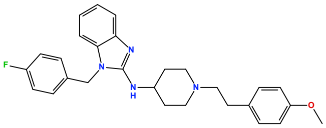

Docking Score: -5.8589

489

DrugBank ID: DB12954

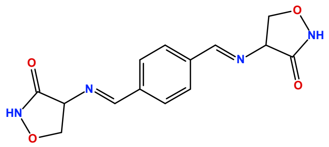

Docking Score: -5.8567

490

DrugBank ID: DB12401

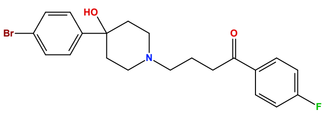

Docking Score: -5.8557

491

DrugBank ID: DB11642

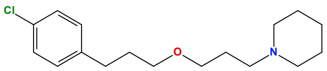

Docking Score: -5.8512

492

DrugBank ID: DB06155

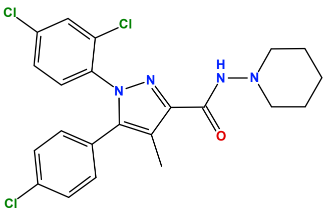

Docking Score: -5.8470

493

DrugBank ID: DB09081

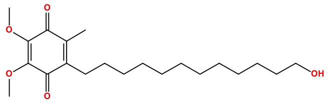

Docking Score: -5.8466

494

DrugBank ID: DB00846

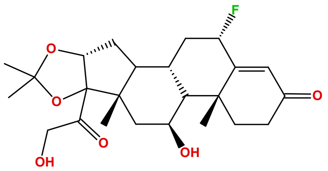

Docking Score: -5.8453

495

DrugBank ID: DB01196

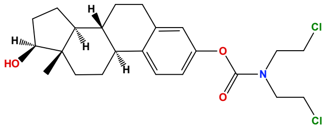

Docking Score: -5.8441

496

DrugBank ID: DB00850

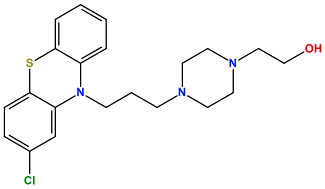

Docking Score: -5.8422

497

DrugBank ID: DB00335

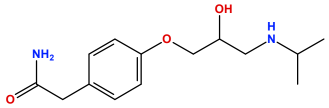

Docking Score: -5.8421

498

DrugBank ID: DB14723

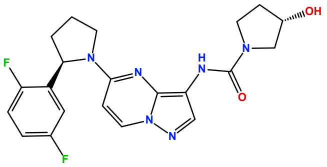

Docking Score: -5.8408

499

DrugBank ID: DB04898

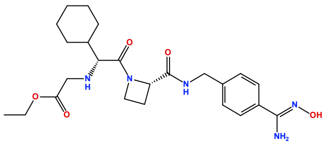

Docking Score: -5.8393

500

DrugBank ID: DB00355

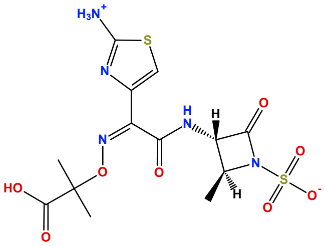

Docking Score: -5.8381

501

DrugBank ID: DB04540

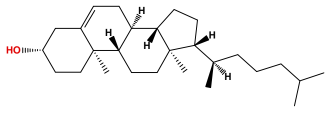

Docking Score: -5.8365

502

DrugBank ID: DB13274

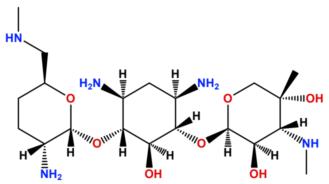

Docking Score: -5.8316

503

DrugBank ID: DB00394

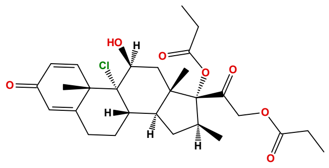

Docking Score: -5.8302

504

DrugBank ID: DB06817

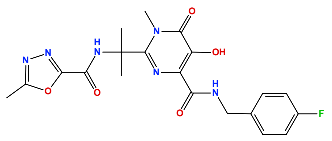

Docking Score: -5.8249

505

DrugBank ID: DB11633

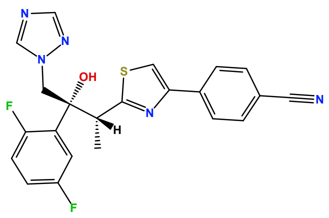

Docking Score: -5.8221

506

DrugBank ID: DB00836

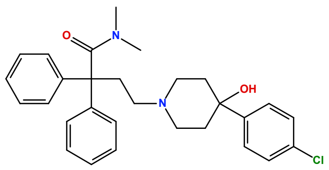

Docking Score: -5.8205

507

DrugBank ID: DB14075

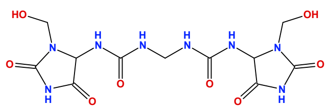

Docking Score: -5.8201

508

DrugBank ID: DB01240

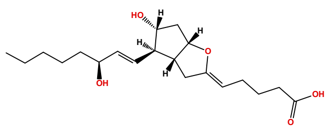

Docking Score: -5.8179

509

DrugBank ID: DB09376

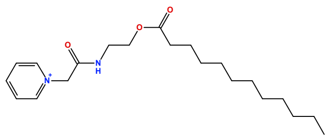

Docking Score: -5.8157

510

DrugBank ID: DB01260

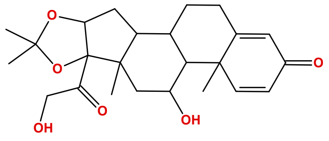

Docking Score: -5.8151

511

DrugBank ID: DB14598

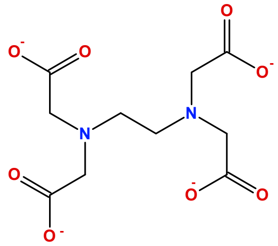

Docking Score: -5.8068

512

DrugBank ID: DB00882

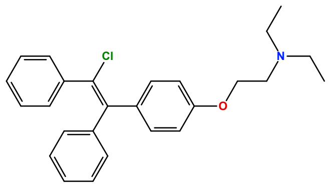

Docking Score: -5.8014

513

DrugBank ID: DB03756

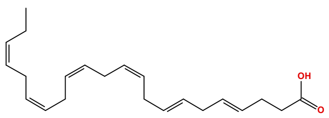

Docking Score: -5.7972

514

DrugBank ID: DB09488

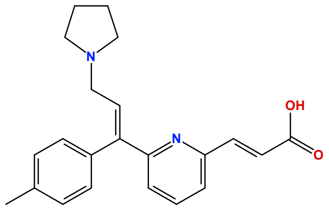

Docking Score: -5.7958

515

DrugBank ID: DB04224

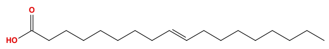

Docking Score: -5.7943

516

DrugBank ID: DB04835

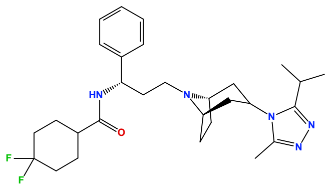

Docking Score: -5.7905

517

DrugBank ID: DB00577

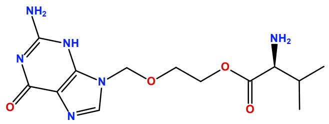

Docking Score: -5.7870

518

DrugBank ID: DB01054

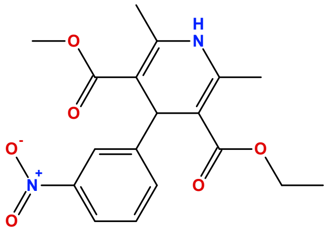

Docking Score: -5.7870

519

DrugBank ID: DB00215

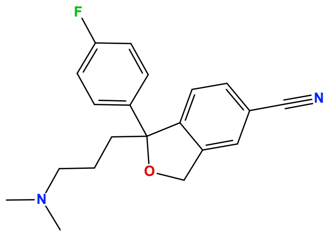

Docking Score: -5.7848

520

DrugBank ID: DB11943

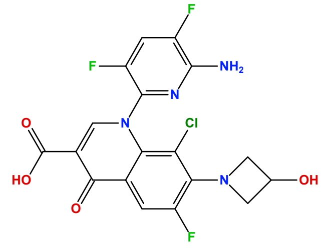

Docking Score: -5.7842

521

DrugBank ID: DB09267

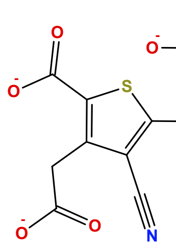

Docking Score: -5.7839

522

DrugBank ID: DB09149

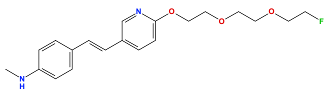

Docking Score: -5.7835

523

DrugBank ID: DB11800

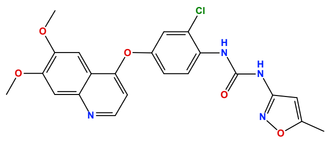

Docking Score: -5.7832

524

DrugBank ID: DB14627

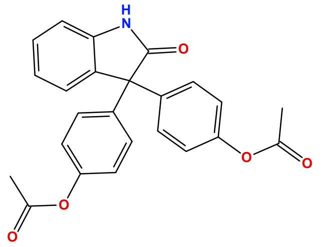

Docking Score: -5.7831

525

DrugBank ID: DB09015

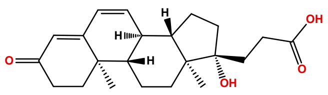

Docking Score: -5.7820

526

DrugBank ID: DB04826

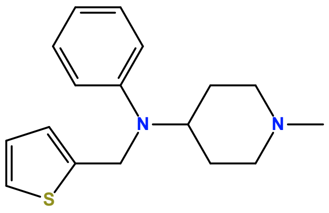

Docking Score: -5.7806

527

DrugBank ID: DB08828

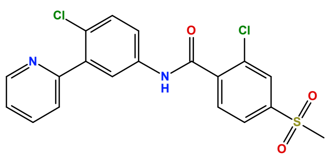

Docking Score: -5.7781

528

DrugBank ID: DB13966

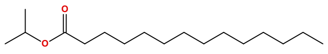

Docking Score: -5.7766

529

DrugBank ID: DB11622

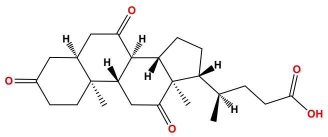

Docking Score: -5.7751

530

DrugBank ID: DB00680

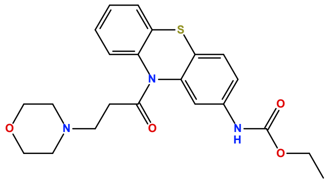

Docking Score: -5.7716

531

DrugBank ID: DB00343

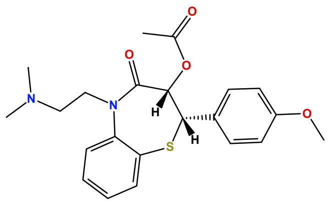

Docking Score: -5.7713

532

DrugBank ID: DB00972

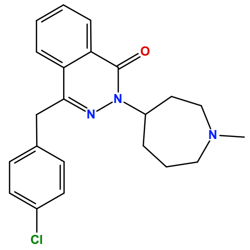

Docking Score: -5.7687

533

DrugBank ID: DB00872

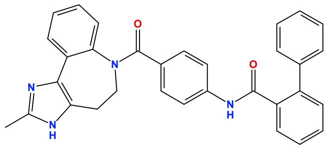

Docking Score: -5.7686

534

DrugBank ID: DB09570

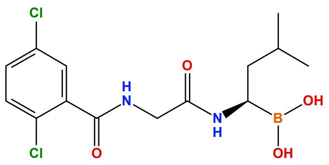

Docking Score: -5.7675

535

DrugBank ID: DB12301

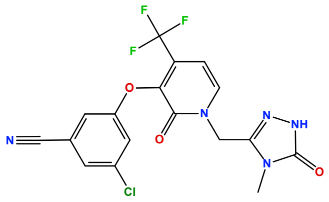

Docking Score: -5.7647

536

DrugBank ID: DB12161

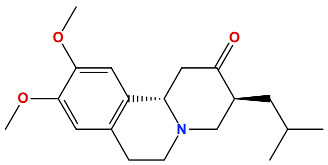

Docking Score: -5.7644

537

DrugBank ID: DB01416

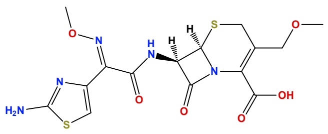

Docking Score: -5.7587

538

DrugBank ID: DB00768

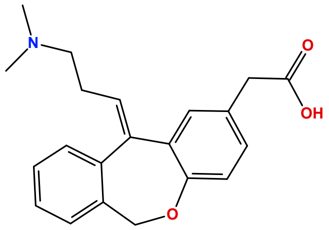

Docking Score: -5.7587

539

DrugBank ID: DB04842

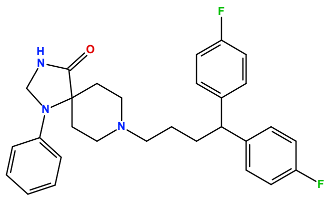

Docking Score: -5.7581

540

DrugBank ID: DB00921

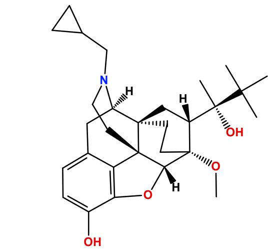

Docking Score: -5.7572

541

DrugBank ID: DB01091

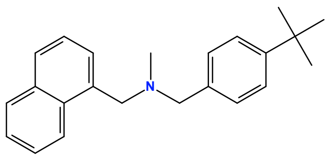

Docking Score: -5.7535

542

DrugBank ID: DB00176

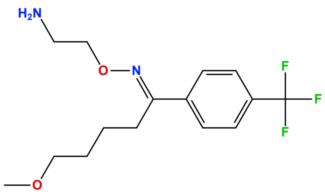

Docking Score: -5.7515

543

DrugBank ID: DB00873

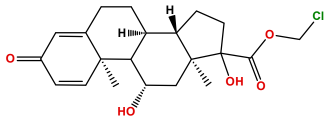

Docking Score: -5.7483

544

DrugBank ID: DB00178

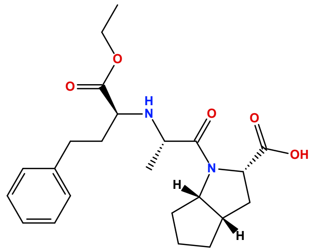

Docking Score: -5.7476

545

DrugBank ID: DB00802

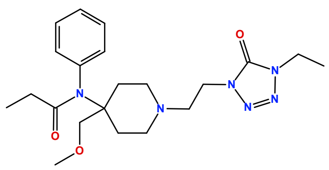

Docking Score: -5.7457

546

DrugBank ID: DB01120

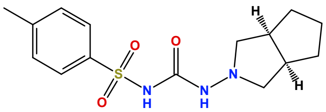

Docking Score: -5.7448

547

DrugBank ID: DB01137

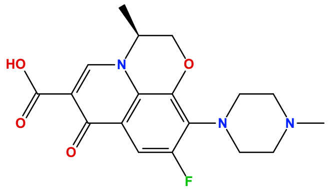

Docking Score: -5.7430

548

DrugBank ID: DB01006

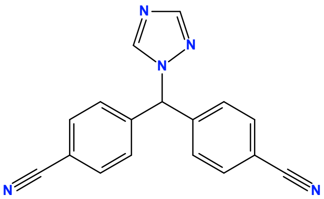

Docking Score: -5.7416

549

DrugBank ID: DB00243

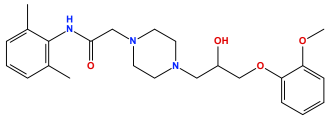

Docking Score: -5.7407

550

DrugBank ID: DB12035

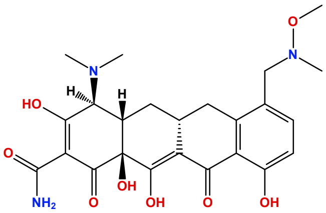

Docking Score: -5.7401

551

DrugBank ID: DB00941

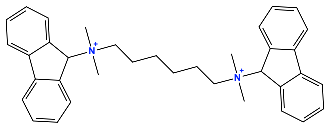

Docking Score: -5.7392

552

DrugBank ID: DB09061

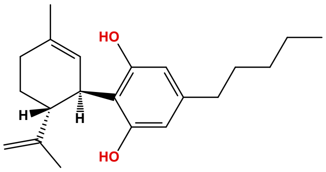

Docking Score: -5.7378

553

DrugBank ID: DB00239

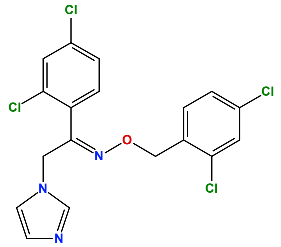

Docking Score: -5.7365

554

DrugBank ID: DB04908

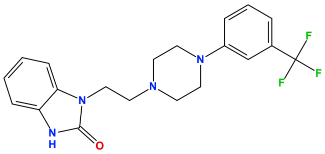

Docking Score: -5.7357

555

DrugBank ID: DB04845

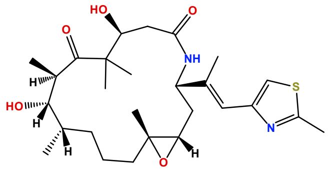

Docking Score: -5.7354

556

DrugBank ID: DB00433

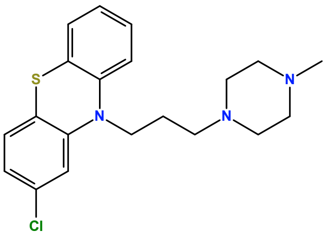

Docking Score: -5.7343

557

DrugBank ID: DB01608

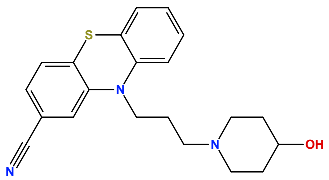

Docking Score: -5.7338

558

DrugBank ID: DB01128

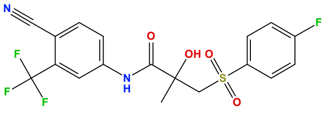

Docking Score: -5.7257

559

DrugBank ID: DB00708

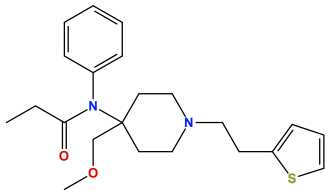

Docking Score: -5.7245

560

DrugBank ID: DB11632

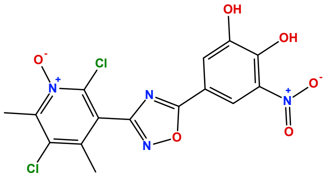

Docking Score: -5.7238

561

DrugBank ID: DB09123

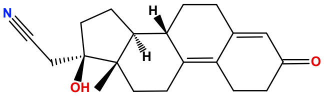

Docking Score: -5.7215

562

DrugBank ID: DB04861

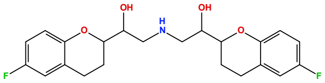

Docking Score: -5.7203

563

DrugBank ID: DB01062

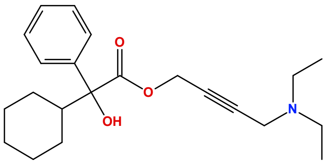

Docking Score: -5.7198

564

DrugBank ID: DB00346

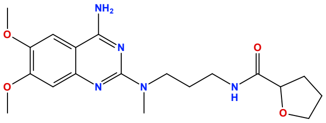

Docking Score: -5.7194

565

DrugBank ID: DB00690

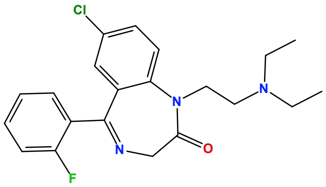

Docking Score: -5.7186

566

DrugBank ID: DB00899

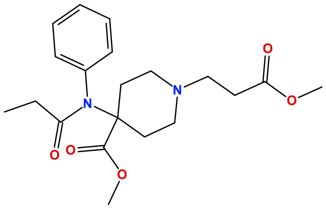

Docking Score: -5.7165

567

DrugBank ID: DB04841

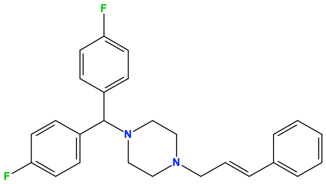

Docking Score: -5.7151

568

DrugBank ID: DB01098

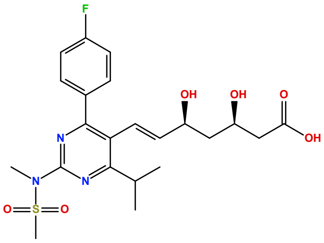

Docking Score: -5.7133

569

DrugBank ID: DB00580

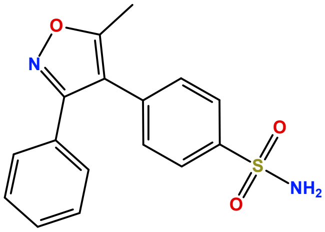

Docking Score: -5.7120

570

DrugBank ID: DB00912

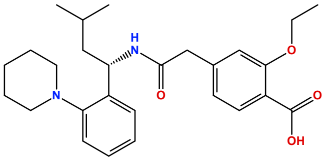

Docking Score: -5.7114

571

DrugBank ID: DB09063

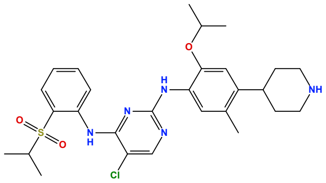

Docking Score: -5.7109

572

DrugBank ID: DB09236

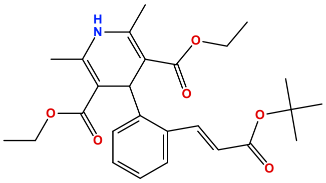

Docking Score: -5.7104

573

DrugBank ID: DB06702

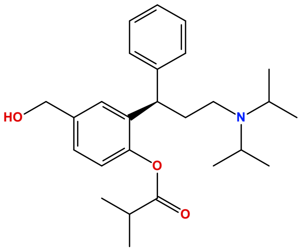

Docking Score: -5.7059

574

DrugBank ID: DB00641

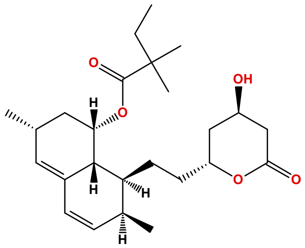

Docking Score: -5.7040

575

DrugBank ID: DB06403

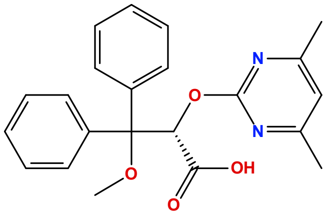

Docking Score: -5.7031

576

DrugBank ID: DB12877

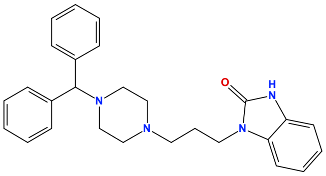

Docking Score: -5.6987

577

DrugBank ID: DB06814

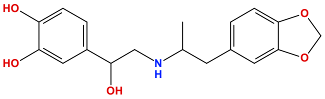

Docking Score: -5.6970

578

DrugBank ID: DB00692

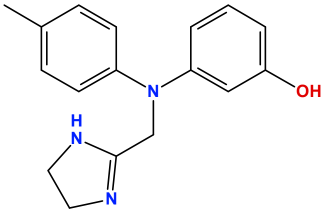

Docking Score: -5.6954

579

DrugBank ID: DB00777

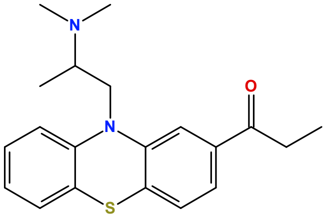

Docking Score: -5.6951

580

DrugBank ID: DB14881

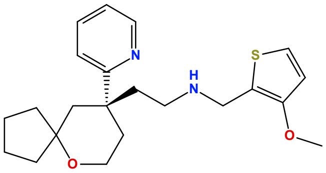

Docking Score: -5.6922

581

DrugBank ID: DB01102

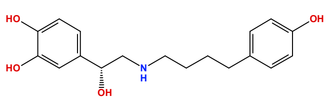

Docking Score: -5.6880

582

DrugBank ID: DB00726

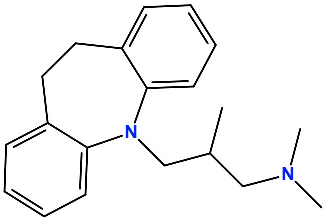

Docking Score: -5.6877

583

DrugBank ID: DB13444

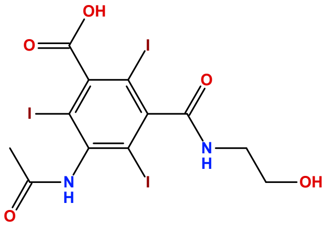

Docking Score: -5.6852

584

DrugBank ID: DB01332

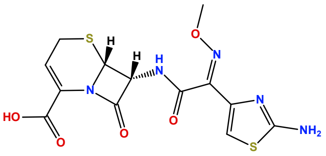

Docking Score: -5.6850

585

DrugBank ID: DB09300

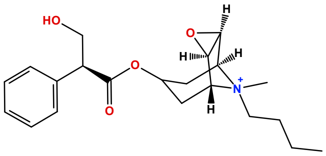

Docking Score: -5.6827

586

DrugBank ID: DB00383

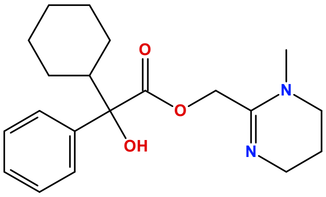

Docking Score: -5.6821

587

DrugBank ID: DB00276

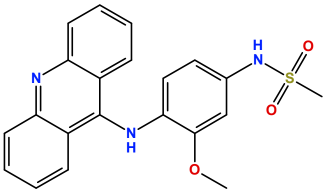

Docking Score: -5.6800

588

DrugBank ID: DB09069

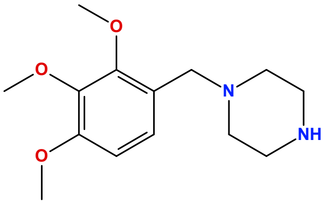

Docking Score: -5.6797

589

DrugBank ID: DB01616

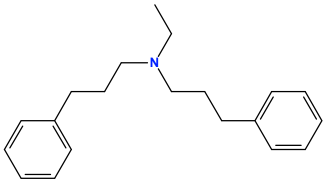

Docking Score: -5.6789

590

DrugBank ID: DB11936

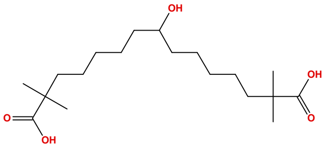

Docking Score: -5.6750

591

DrugBank ID: DB11186

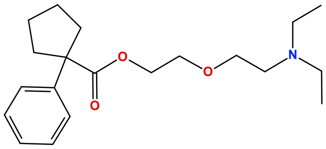

Docking Score: -5.6747

592

DrugBank ID: DB04817

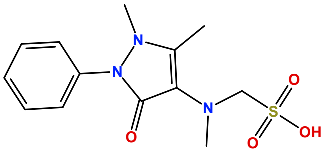

Docking Score: -5.6746

593

DrugBank ID: DB06603

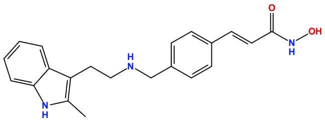

Docking Score: -5.6737

594

DrugBank ID: DB00421

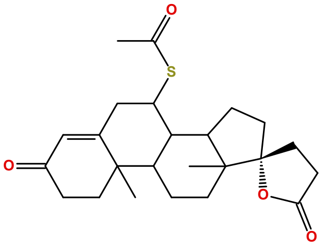

Docking Score: -5.6732

595

DrugBank ID: DB00338

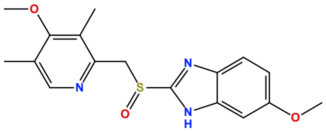

Docking Score: -5.6712

596

DrugBank ID: DB00962

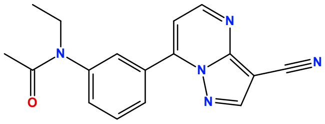

Docking Score: -5.6705

597

DrugBank ID: DB11901

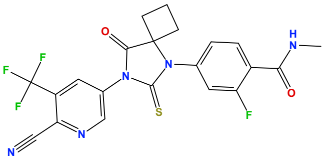

Docking Score: -5.6695

598

DrugBank ID: DB01130

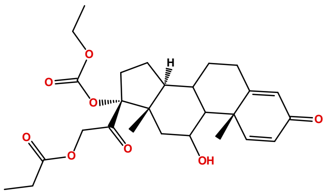

Docking Score: -5.6632

599

DrugBank ID: DB06820

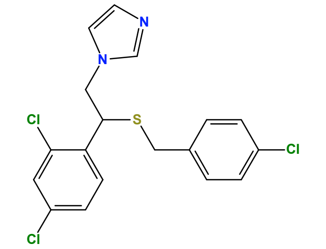

Docking Score: -5.6598

600

DrugBank ID: DB01409

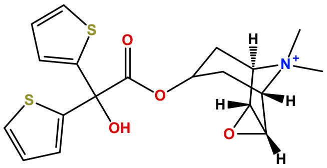

Docking Score: -5.6575

601

DrugBank ID: DB06712

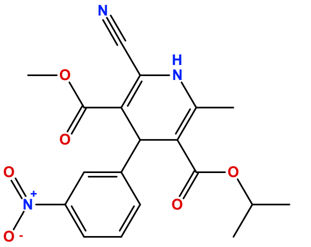

Docking Score: -5.6562

602

DrugBank ID: DB01452

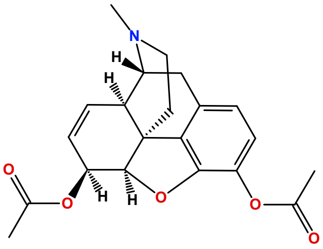

Docking Score: -5.6558

603

DrugBank ID: DB09209

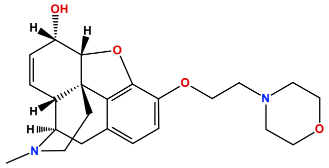

Docking Score: -5.6550

604

DrugBank ID: DB01146

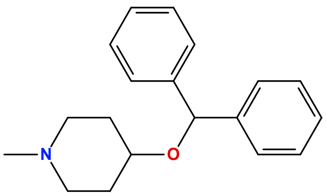

Docking Score: -5.6539

605

DrugBank ID: DB08965

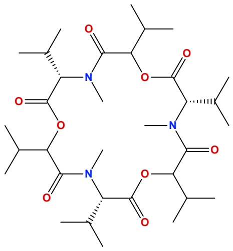

Docking Score: -5.6533

606

DrugBank ID: DB13954

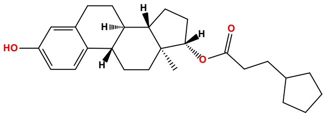

Docking Score: -5.6511

607

DrugBank ID: DB08881

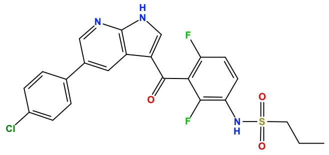

Docking Score: -5.6502

608

DrugBank ID: DB00490

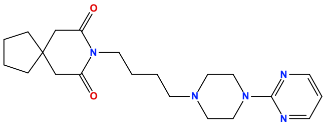

Docking Score: -5.6484

609

DrugBank ID: DB11712

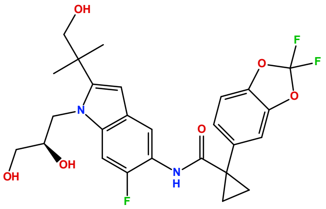

Docking Score: -5.6467

610

DrugBank ID: DB00308

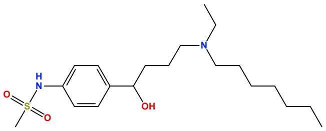

Docking Score: -5.6429

611

DrugBank ID: DB00179

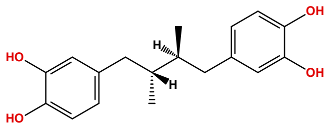

Docking Score: -5.6422

612

DrugBank ID: DB14196

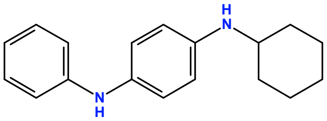

Docking Score: -5.6420

613

DrugBank ID: DB09078

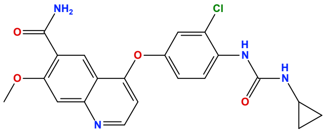

Docking Score: -5.6415

614

DrugBank ID: DB01145

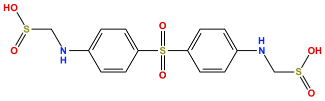

Docking Score: -5.6384

615

DrugBank ID: DB00678

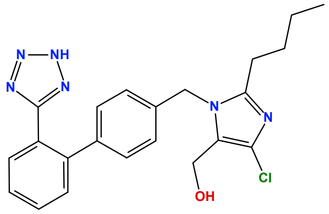

Docking Score: -5.6381

616

DrugBank ID: DB04953

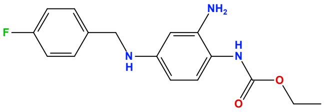

Docking Score: -5.6368

617

DrugBank ID: DB04832

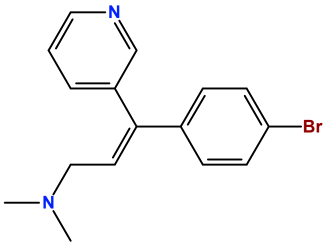

Docking Score: -5.6297

618

DrugBank ID: DB01359

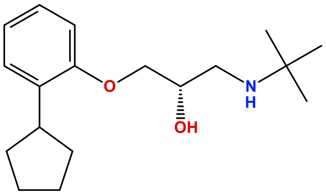

Docking Score: -5.6248

619

DrugBank ID: DB11629

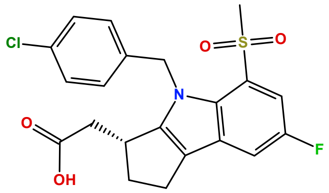

Docking Score: -5.6239

620

DrugBank ID: DB00218

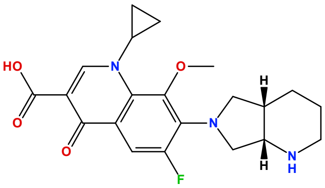

Docking Score: -5.6218

621

DrugBank ID: DB00146

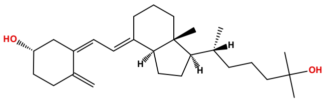

Docking Score: -5.6212

622

DrugBank ID: DB06213

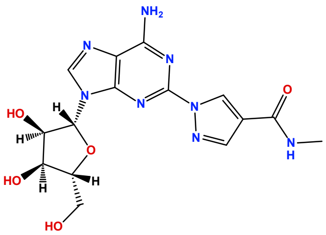

Docking Score: -5.6211

623

DrugBank ID: DB06211

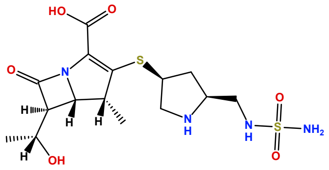

Docking Score: -5.6196

624

DrugBank ID: DB00735

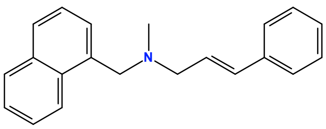

Docking Score: -5.6191

625

DrugBank ID: DB01219

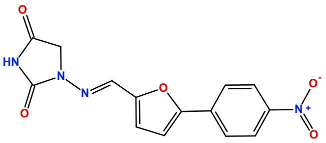

Docking Score: -5.6185

626

DrugBank ID: DB00309

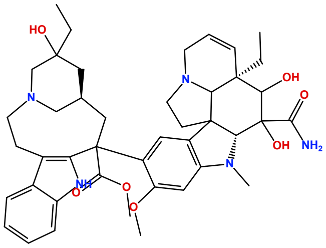

Docking Score: -5.6184

627

DrugBank ID: DB11757

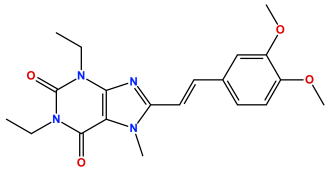

Docking Score: -5.6184

628

DrugBank ID: DB06212

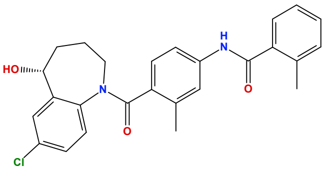

Docking Score: -5.6180

629

DrugBank ID: DB01179

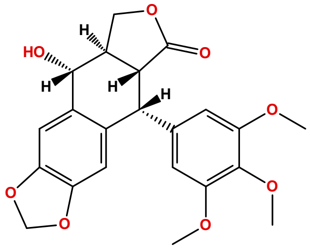

Docking Score: -5.6173

630

DrugBank ID: DB06176

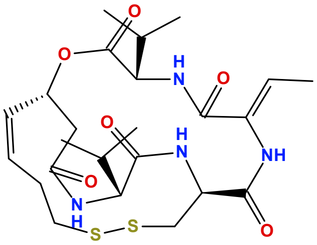

Docking Score: -5.6168

631

DrugBank ID: DB06614

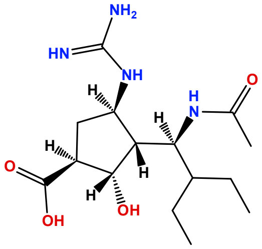

Docking Score: -5.6152

632

DrugBank ID: DB00795

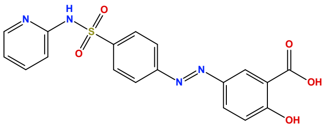

Docking Score: -5.6149

633

DrugBank ID: DB09034

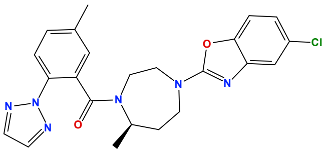

Docking Score: -5.6148

634

DrugBank ID: DB00578

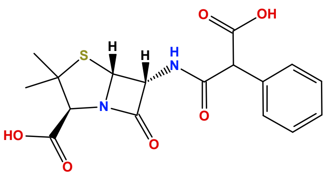

Docking Score: -5.6148

635

DrugBank ID: DB11760

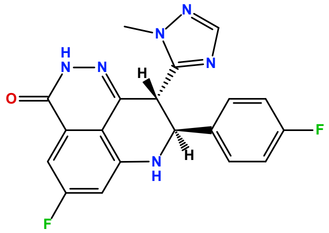

Docking Score: -5.6122

636

DrugBank ID: DB11125

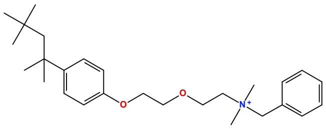

Docking Score: -5.6111

637

DrugBank ID: DB01394

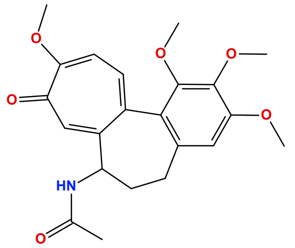

Docking Score: -5.6109

638

DrugBank ID: DB01203

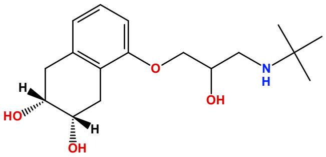

Docking Score: -5.6105

639

DrugBank ID: DB01060

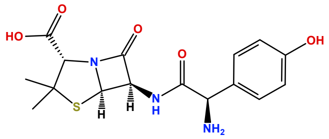

Docking Score: -5.6102

640

DrugBank ID: DB01624

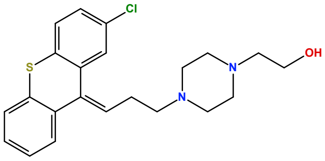

Docking Score: -5.6087

641

DrugBank ID: DB00892

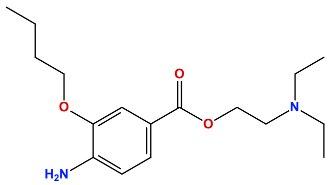

Docking Score: -5.6078

642

DrugBank ID: DB00227

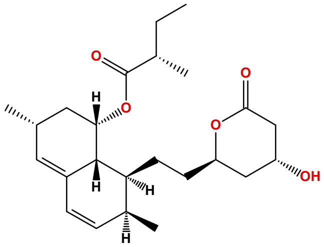

Docking Score: -5.6078

643

DrugBank ID: DB00957

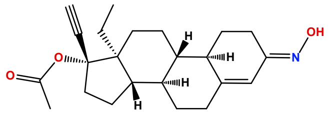

Docking Score: -5.6051

644

DrugBank ID: DB01501

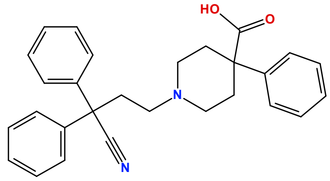

Docking Score: -5.6050

645

DrugBank ID: DB14512

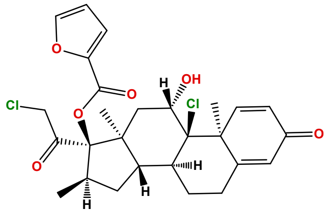

Docking Score: -5.6034

646

DrugBank ID: DB00487

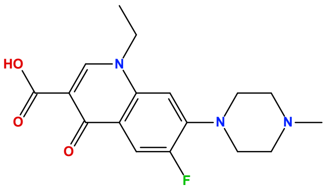

Docking Score: -5.6028

647

DrugBank ID: DB09080

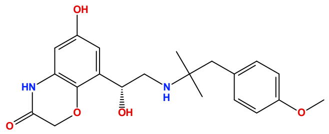

Docking Score: -5.6010

648

DrugBank ID: DB09097

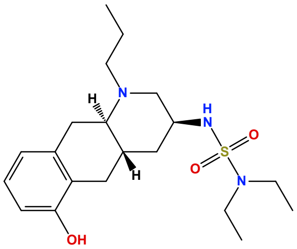

Docking Score: -5.6008

649

DrugBank ID: DB06769

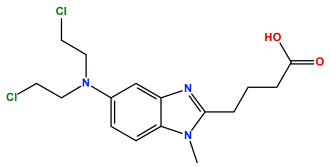

Docking Score: -5.5998

650

DrugBank ID: DB11073

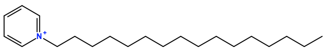

Docking Score: -5.5997

651

DrugBank ID: DB01048

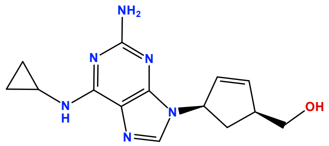

Docking Score: -5.5980

652

DrugBank ID: DB01246

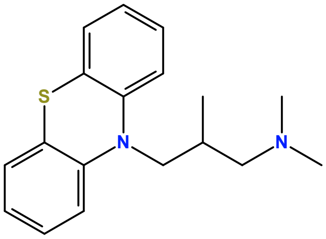

Docking Score: -5.5958

653

DrugBank ID: DB00995

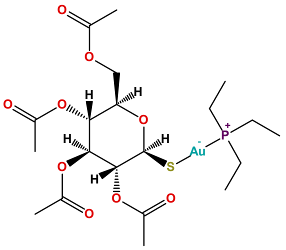

Docking Score: -5.5945

654

DrugBank ID: DB00271

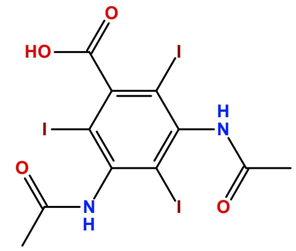

Docking Score: -5.5937

655

DrugBank ID: DB00214

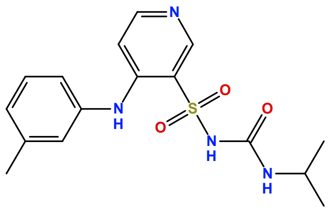

Docking Score: -5.5933

656

DrugBank ID: DB11656

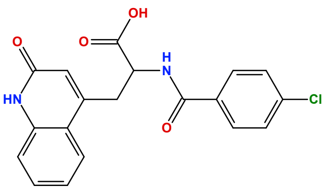

Docking Score: -5.5926

657

DrugBank ID: DB00731

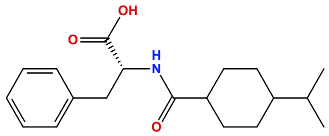

Docking Score: -5.5911

658

DrugBank ID: DB04794

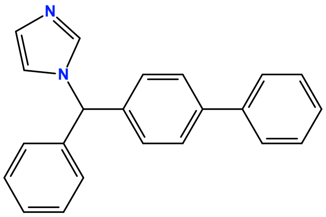

Docking Score: -5.5899

659

DrugBank ID: DB06626

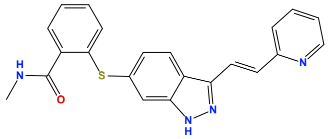

Docking Score: -5.5891

660

DrugBank ID: DB13279

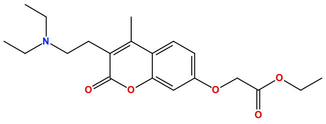

Docking Score: -5.5861

661

DrugBank ID: DB12404

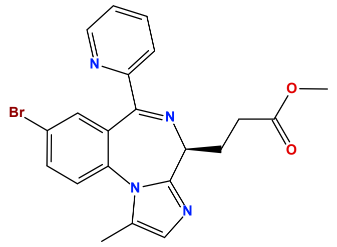

Docking Score: -5.5857

662

DrugBank ID: DB04938

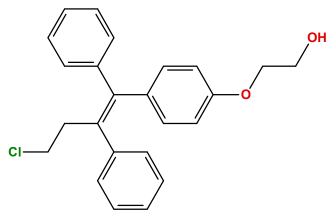

Docking Score: -5.5835

663

DrugBank ID: DB06751

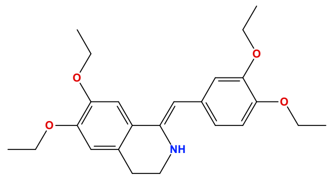

Docking Score: -5.5828

664

DrugBank ID: DB00438

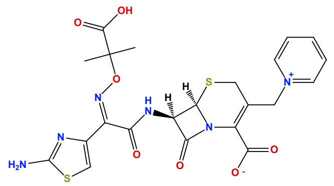

Docking Score: -5.5817

665

DrugBank ID: DB00477

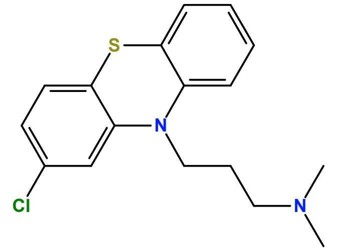

Docking Score: -5.5808

666

DrugBank ID: DB00918

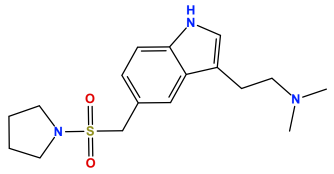

Docking Score: -5.5784

667

DrugBank ID: DB14845

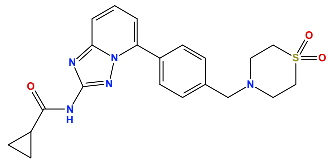

Docking Score: -5.5758

668

DrugBank ID: DB00264

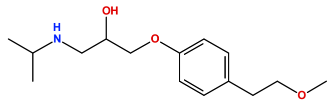

Docking Score: -5.5750

669

DrugBank ID: DB11594

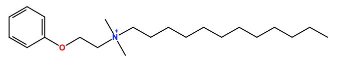

Docking Score: -5.5736

670

DrugBank ID: DB08907

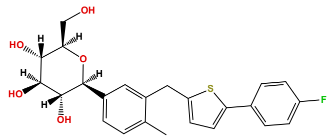

Docking Score: -5.5735

671

DrugBank ID: DB09089

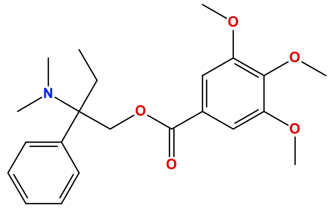

Docking Score: -5.5726

672

DrugBank ID: DB09054

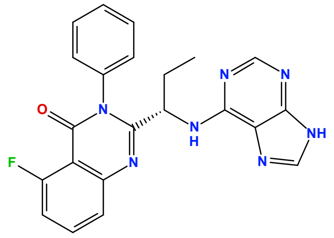

Docking Score: -5.5712

673

DrugBank ID: DB01150

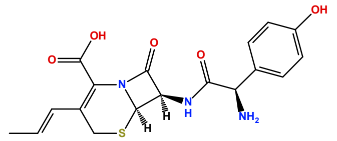

Docking Score: -5.5701

674

DrugBank ID: DB08958

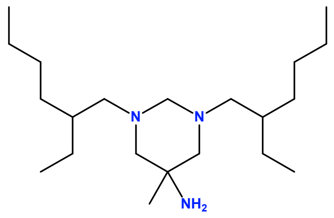

Docking Score: -5.5675

675

DrugBank ID: DB01013

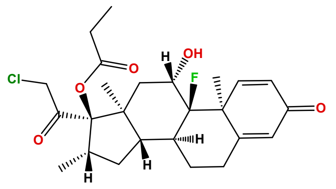

Docking Score: -5.5660

676

DrugBank ID: DB09496

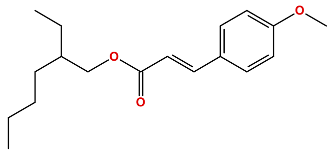

Docking Score: -5.5652

677

DrugBank ID: DB00906

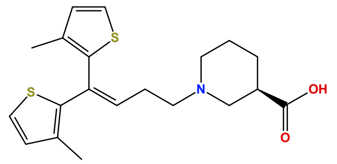

Docking Score: -5.5609

678

DrugBank ID: DB00143

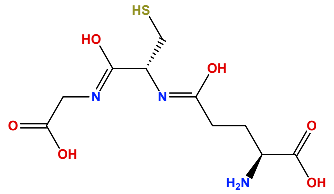

Docking Score: -5.5608

679

DrugBank ID: DB06217

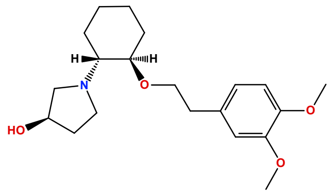

Docking Score: -5.5568

680

DrugBank ID: DB13532

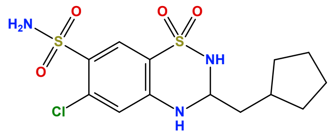

Docking Score: -5.5555

681

DrugBank ID: DB00470

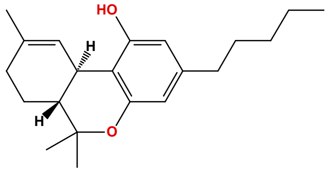

Docking Score: -5.5535

682

DrugBank ID: DB00902

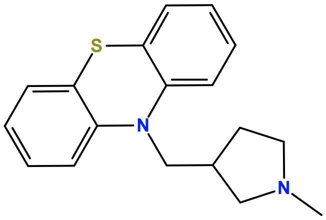

Docking Score: -5.5517

683

DrugBank ID: DB00833

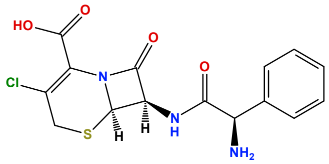

Docking Score: -5.5505

684

DrugBank ID: DB00247

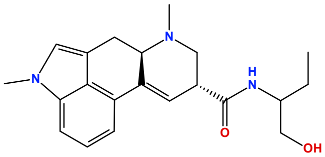

Docking Score: -5.5498

685

DrugBank ID: DB11577

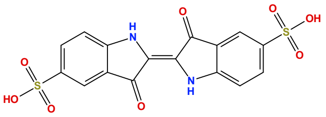

Docking Score: -5.5465

686

DrugBank ID: DB09076

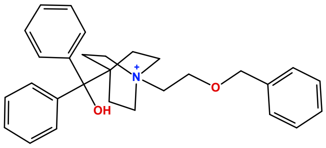

Docking Score: -5.5458

687

DrugBank ID: DB13657

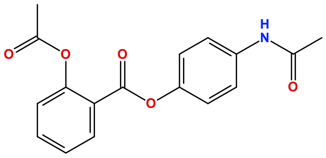

Docking Score: -5.5446

688

DrugBank ID: DB14753

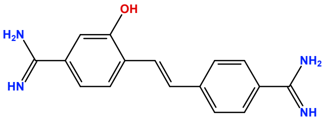

Docking Score: -5.5440

689

DrugBank ID: DB01586

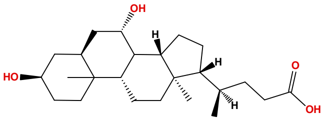

Docking Score: -5.5425

690

DrugBank ID: DB14643

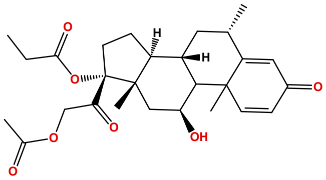

Docking Score: -5.5417

691

DrugBank ID: DB13953

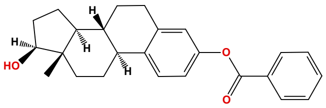

Docking Score: -5.5395

692

DrugBank ID: DB05039

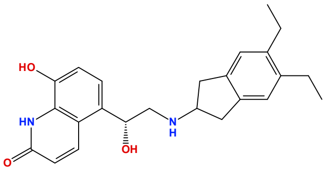

Docking Score: -5.5389

693

DrugBank ID: DB00192

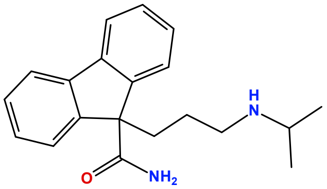

Docking Score: -5.5385

694

DrugBank ID: DB00887

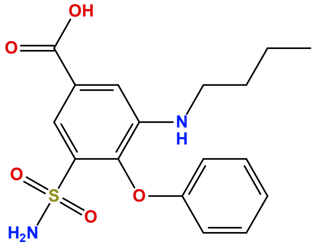

Docking Score: -5.5375

695

DrugBank ID: DB00940

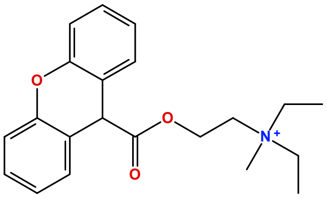

Docking Score: -5.5365

696

DrugBank ID: DB06654

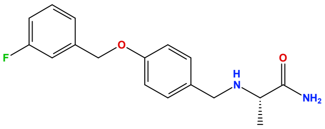

Docking Score: -5.5365

697

DrugBank ID: DB00805

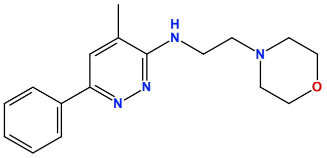

Docking Score: -5.5364

698

DrugBank ID: DB01118

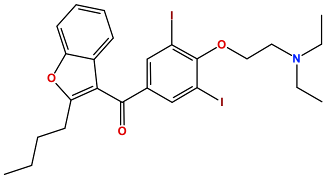

Docking Score: -5.5327

699

DrugBank ID: DB01603

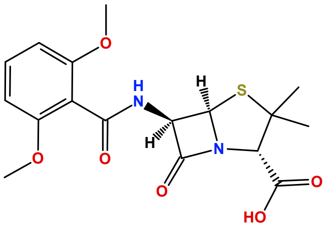

Docking Score: -5.5322

700

DrugBank ID: DB04822

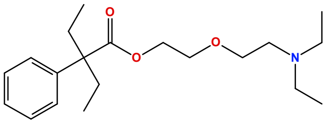

Docking Score: -5.5319

701

DrugBank ID: DB01340

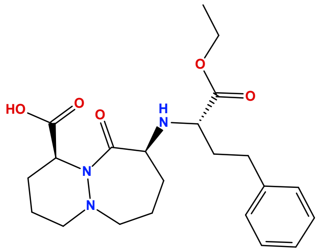

Docking Score: -5.5314

702

DrugBank ID: DB00876

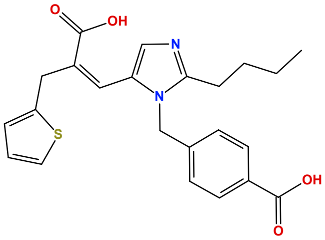

Docking Score: -5.5291

703

DrugBank ID: DB00283

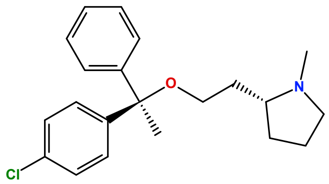

Docking Score: -5.5289

704

DrugBank ID: DB01418

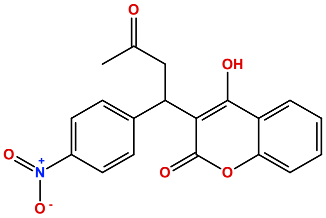

Docking Score: -5.5262

705

DrugBank ID: DB01588

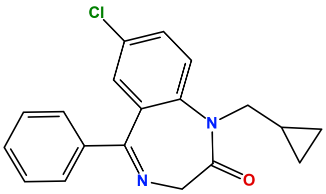

Docking Score: -5.5256

706

DrugBank ID: DB01023

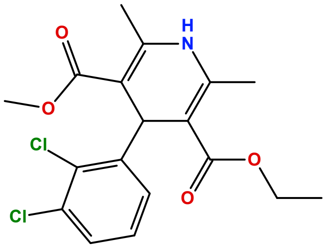

Docking Score: -5.5224

707

DrugBank ID: DB09477

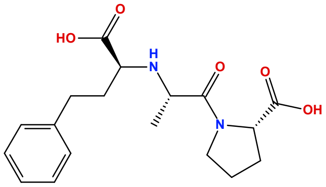

Docking Score: -5.5193

708

DrugBank ID: DB06816

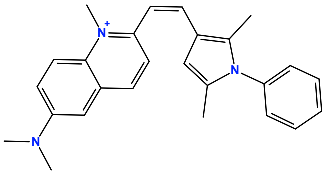

Docking Score: -5.5182

709

DrugBank ID: DB01194

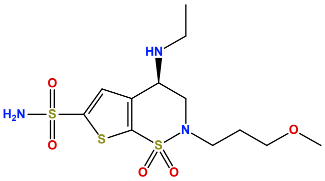

Docking Score: -5.5159

710

DrugBank ID: DB09268

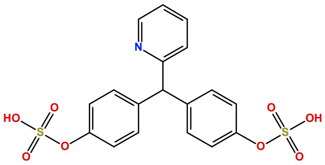

Docking Score: -5.5151

711

DrugBank ID: DB04839

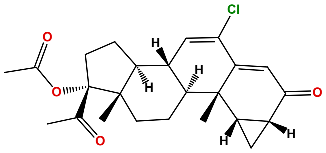

Docking Score: -5.5123

712

DrugBank ID: DB01161

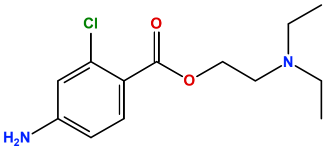

Docking Score: -5.5121

713

DrugBank ID: DB00571

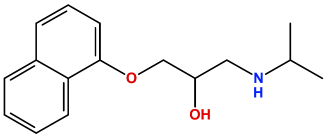

Docking Score: -5.5118

714

DrugBank ID: DB01430

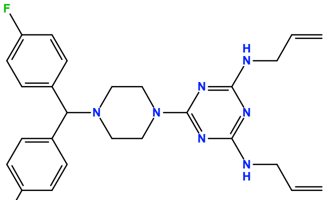

Docking Score: -5.5102

715

DrugBank ID: DB11732

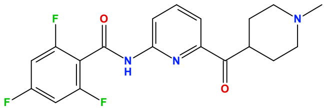

Docking Score: -5.5079

716

DrugBank ID: DB04209

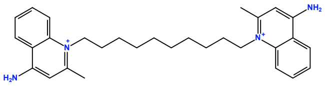

Docking Score: -5.5071

717

DrugBank ID: DB15477

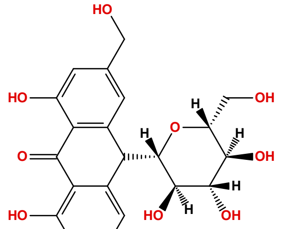

Docking Score: -5.5057

718

DrugBank ID: DB00213

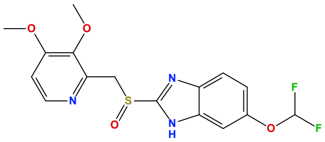

Docking Score: -5.5055

719

DrugBank ID: DB12523

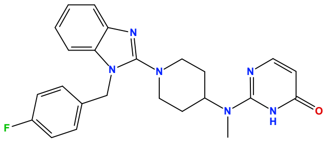

Docking Score: -5.5042

720

DrugBank ID: DB01237

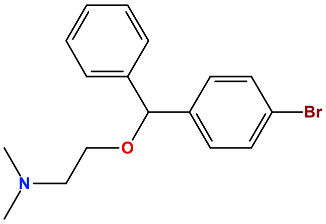

Docking Score: -5.5015

721

DrugBank ID: DB01598

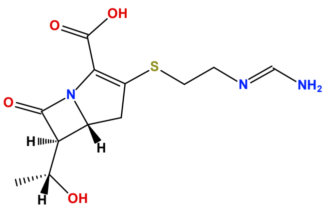

Docking Score: -5.4982

722

DrugBank ID: DB01117

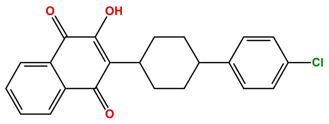

Docking Score: -5.4966

723

DrugBank ID: DB00747

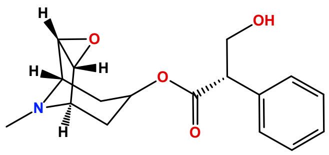

Docking Score: -5.4965

724

DrugBank ID: DB01415

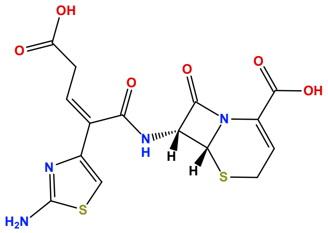

Docking Score: -5.4962

725

DrugBank ID: DB00285

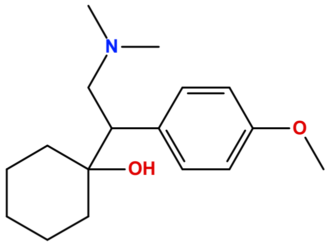

Docking Score: -5.4944

726

DrugBank ID: DB14195

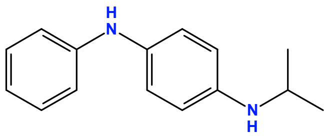

Docking Score: -5.4922

727

DrugBank ID: DB11994

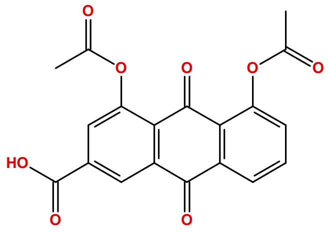

Docking Score: -5.4913

728

DrugBank ID: DB11089

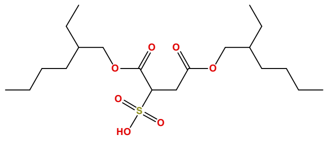

Docking Score: -5.4904

729

DrugBank ID: DB00715

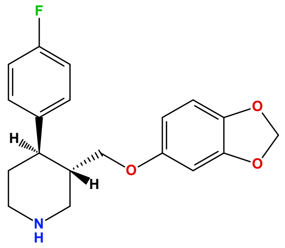

Docking Score: -5.4894

730

DrugBank ID: DB00865

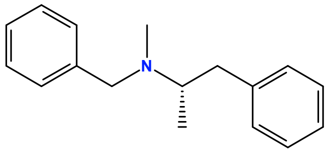

Docking Score: -5.4878

731

DrugBank ID: DB09421

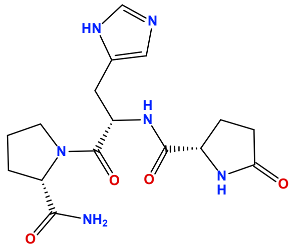

Docking Score: -5.4863

732

DrugBank ID: DB14083

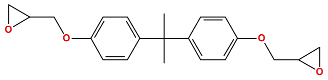

Docking Score: -5.4858

733

DrugBank ID: DB00152

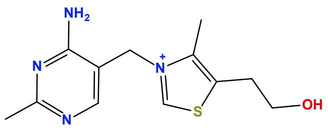

Docking Score: -5.4807

734

DrugBank ID: DB01155

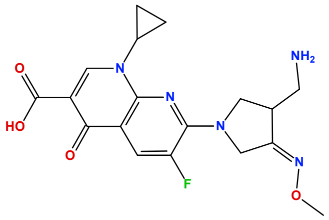

Docking Score: -5.4806

735

DrugBank ID: DB00429

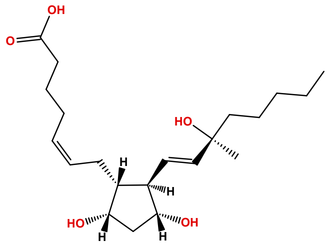

Docking Score: -5.4803

736

DrugBank ID: DB12107

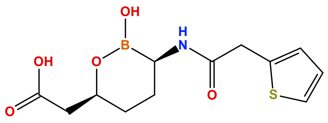

Docking Score: -5.4773

737

DrugBank ID: DB00813

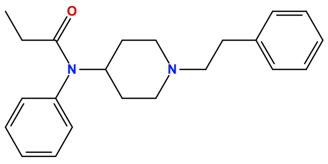

Docking Score: -5.4767

738

DrugBank ID: DB00647

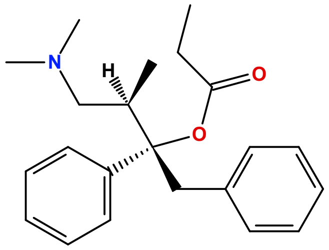

Docking Score: -5.4766

739

DrugBank ID: DB01029

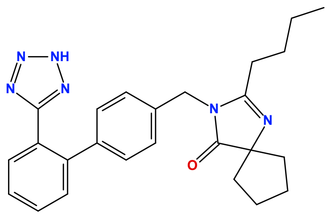

Docking Score: -5.4739

740

DrugBank ID: DB13288

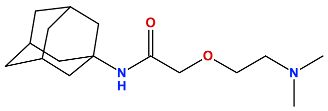

Docking Score: -5.4728

741

DrugBank ID: DB12728

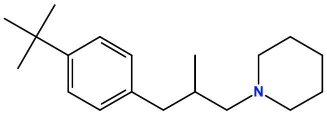

Docking Score: -5.4723

742

DrugBank ID: DB00543

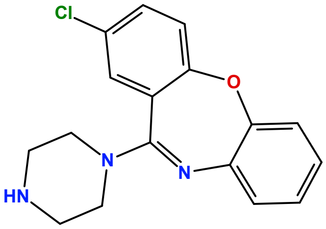

Docking Score: -5.4713

743

DrugBank ID: DB01148

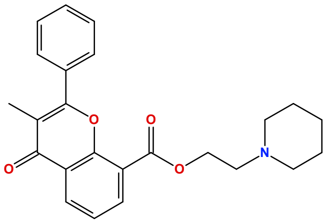

Docking Score: -5.4699

744

DrugBank ID: DB00270

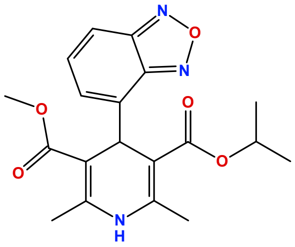

Docking Score: -5.4680

745

DrugBank ID: DB01039

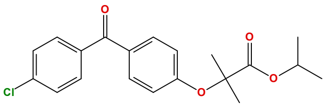

Docking Score: -5.4665

746

DrugBank ID: DB00896

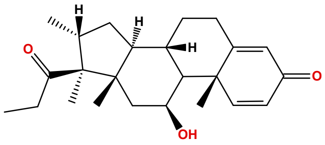

Docking Score: -5.4649

747

DrugBank ID: DB00741

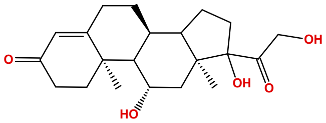

Docking Score: -5.4642

748

DrugBank ID: DB00942

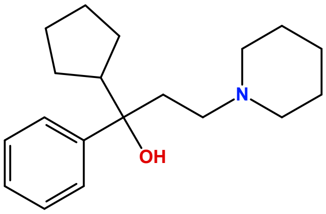

Docking Score: -5.4634

749

DrugBank ID: DB01132

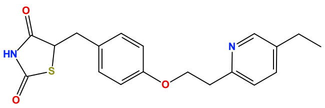

Docking Score: -5.4628

750

DrugBank ID: DB01095

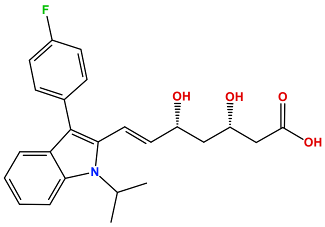

Docking Score: -5.4604

751

DrugBank ID: DB11793

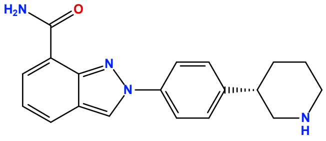

Docking Score: -5.4569

752

DrugBank ID: DB00162

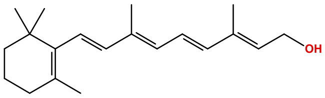

Docking Score: -5.4567

753

DrugBank ID: DB14543

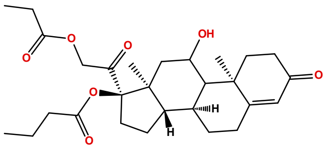

Docking Score: -5.4561

754

DrugBank ID: DB02703

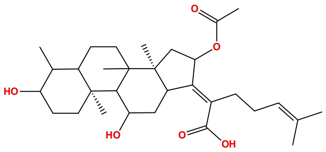

Docking Score: -5.4555

755

DrugBank ID: DB01611

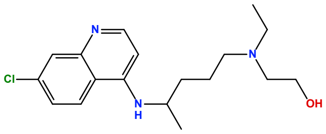

Docking Score: -5.4540

756

DrugBank ID: DB00507

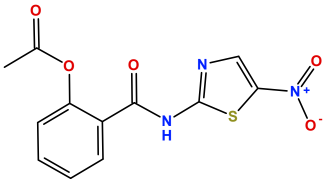

Docking Score: -5.4476

757

DrugBank ID: DB11260

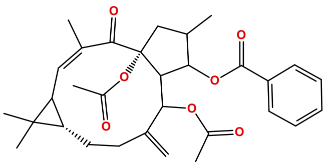

Docking Score: -5.4469

758

DrugBank ID: DB13246

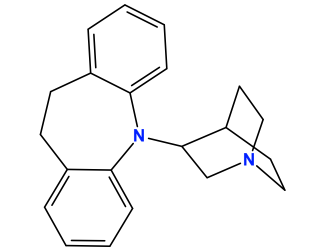

Docking Score: -5.4465

759

DrugBank ID: DB00728

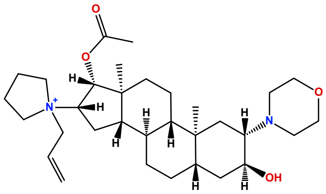

Docking Score: -5.4450

760

DrugBank ID: DB13153

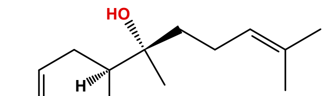

Docking Score: -5.4389

761

DrugBank ID: DB01138

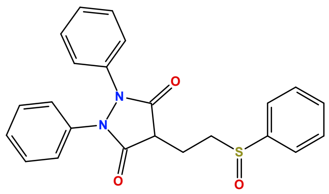

Docking Score: -5.4364

762

DrugBank ID: DB06230

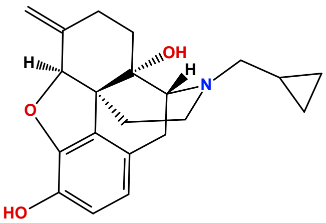

Docking Score: -5.4360

763

DrugBank ID: DB01320

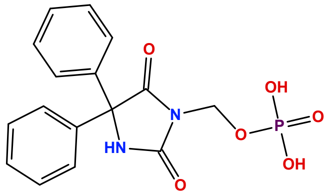

Docking Score: -5.4359

764

DrugBank ID: DB00588

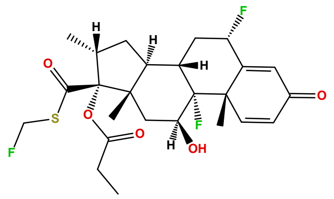

Docking Score: -5.4352

765

DrugBank ID: DB14657

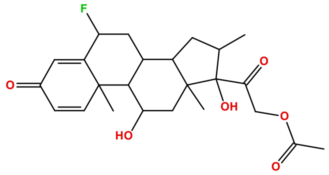

Docking Score: -5.4313

766

DrugBank ID: DB00521

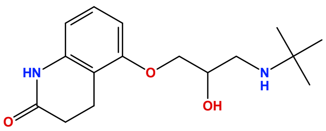

Docking Score: -5.4306

767

DrugBank ID: DB09495

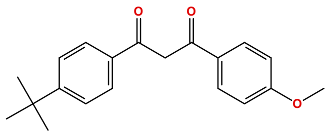

Docking Score: -5.4302

768

DrugBank ID: DB04820

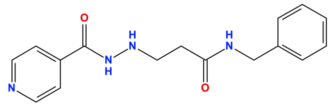

Docking Score: -5.4295

769

DrugBank ID: DB11155

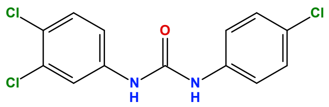

Docking Score: -5.4293

770

DrugBank ID: DB11952

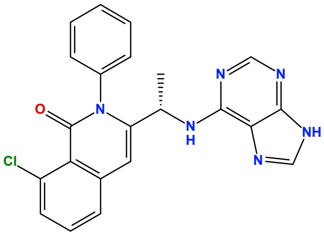

Docking Score: -5.4283

771

DrugBank ID: DB11207

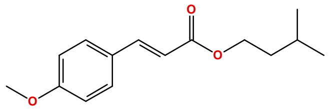

Docking Score: -5.4282

772

DrugBank ID: DB01217

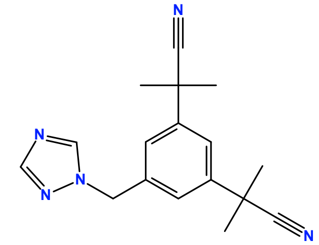

Docking Score: -5.4276

773

DrugBank ID: DB09056

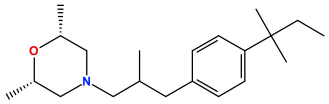

Docking Score: -5.4276

774

DrugBank ID: DB00291

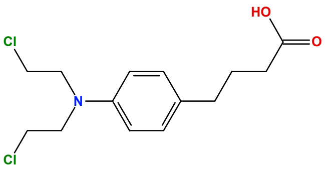

Docking Score: -5.4262

775

DrugBank ID: DB00340

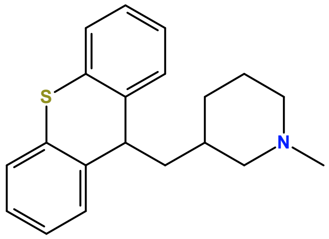

Docking Score: -5.4261

776

DrugBank ID: DB14174

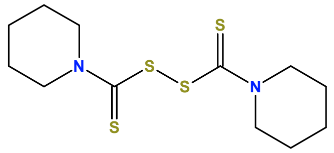

Docking Score: -5.4260

777

DrugBank ID: DB11678

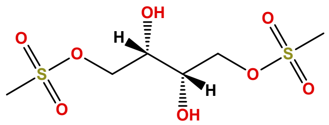

Docking Score: -5.4252

778

DrugBank ID: DB12825

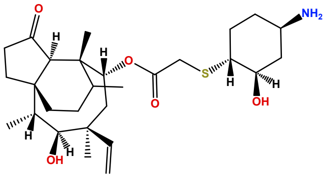

Docking Score: -5.4247

779

DrugBank ID: DB01100

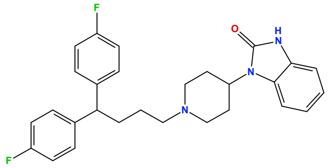

Docking Score: -5.4244

780

DrugBank ID: DB00439

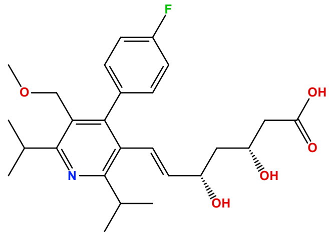

Docking Score: -5.4238

781

DrugBank ID: DB08936

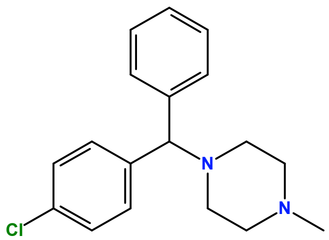

Docking Score: -5.4196

782

DrugBank ID: DB12473

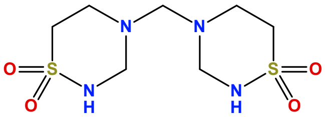

Docking Score: -5.4156

783

DrugBank ID: DB14033

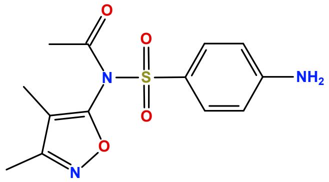

Docking Score: -5.4147

784

DrugBank ID: DB01058

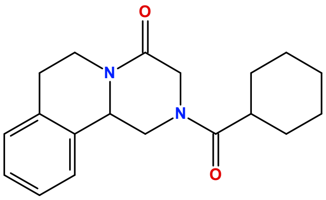

Docking Score: -5.4137

785

DrugBank ID: DB13956

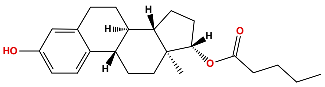

Docking Score: -5.4134

786

DrugBank ID: DB00589

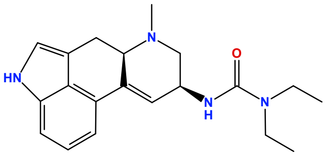

Docking Score: -5.4127

787

DrugBank ID: DB01168

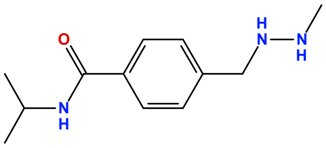

Docking Score: -5.4126

788

DrugBank ID: DB00393

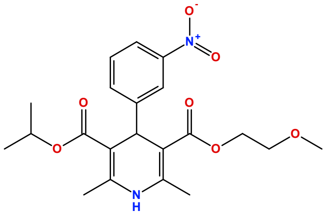

Docking Score: -5.4115

789

DrugBank ID: DB00567

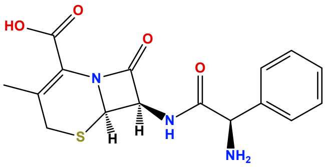

Docking Score: -5.4113

790

DrugBank ID: DB13247

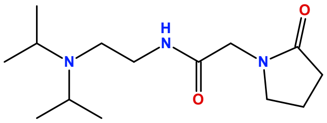

Docking Score: -5.4112

791

DrugBank ID: DB01106

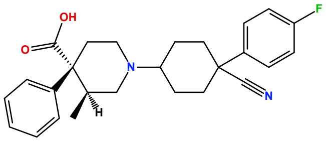

Docking Score: -5.4111

792

DrugBank ID: DB01024

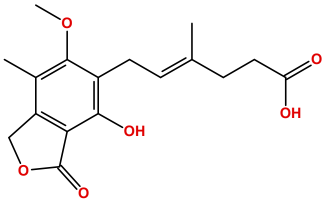

Docking Score: -5.4107

793

DrugBank ID: DB00484

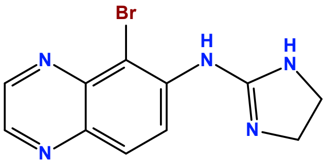

Docking Score: -5.4088

794

DrugBank ID: DB12070

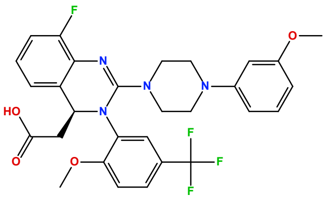

Docking Score: -5.4035

795

DrugBank ID: DB00527

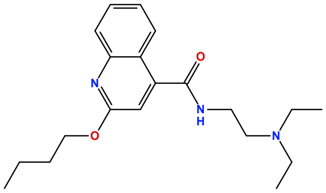

Docking Score: -5.4001

796

DrugBank ID: DB12020

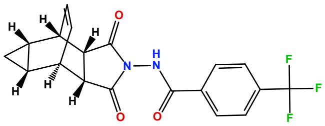

Docking Score: -5.3996

797

DrugBank ID: DB13997

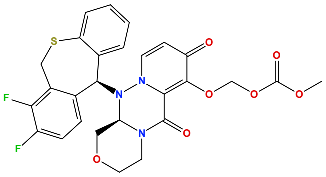

Docking Score: -5.3989

798

DrugBank ID: DB08899

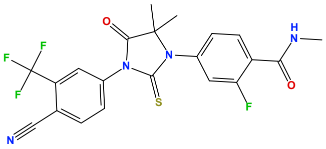

Docking Score: -5.3978

799

DrugBank ID: DB00841

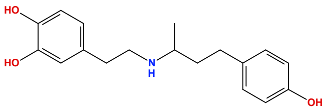

Docking Score: -5.3948

800

DrugBank ID: DB00298

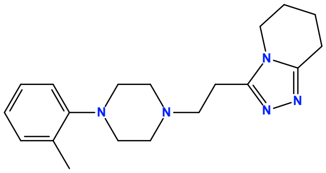

Docking Score: -5.3911

801

DrugBank ID: DB08801

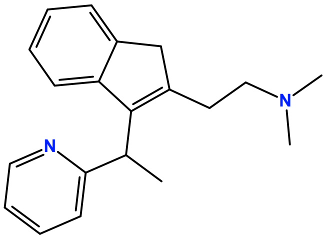

Docking Score: -5.3906

802

DrugBank ID: DB06771

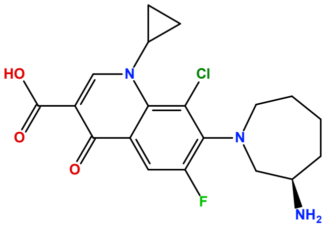

Docking Score: -5.3897

803

DrugBank ID: DB00601

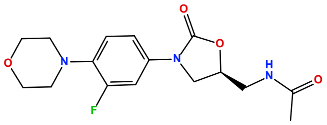

Docking Score: -5.3891

804

DrugBank ID: DB05271

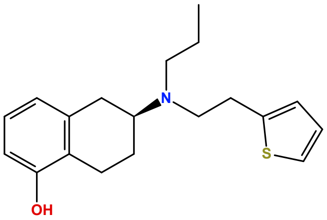

Docking Score: -5.3870

805

DrugBank ID: DB00501

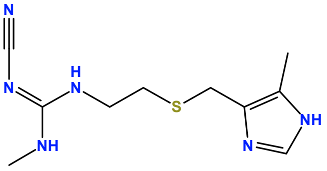

Docking Score: -5.3841

806

DrugBank ID: DB11942

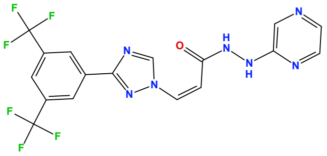

Docking Score: -5.3808

807

DrugBank ID: DB00436

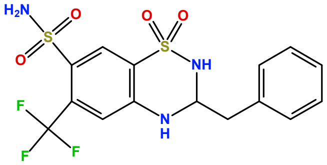

Docking Score: -5.3807

808

DrugBank ID: DB06781

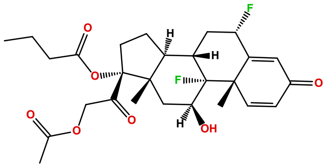

Docking Score: -5.3804

809

DrugBank ID: DB00975

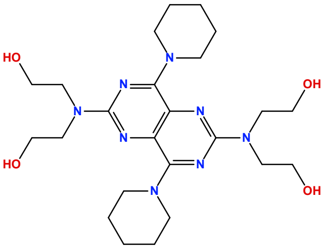

Docking Score: -5.3767

810

DrugBank ID: DB00472

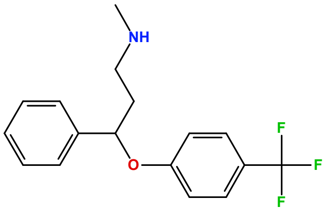

Docking Score: -5.3755

811

DrugBank ID: DB00636

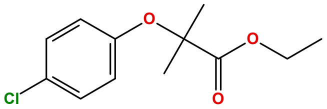

Docking Score: -5.3744

812

DrugBank ID: DB00249

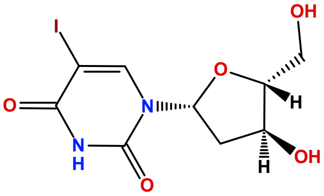

Docking Score: -5.3729

813

DrugBank ID: DB05013

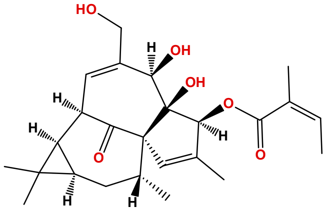

Docking Score: -5.3712

814

DrugBank ID: DB02546

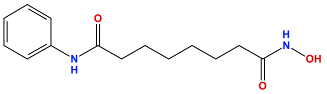

Docking Score: -5.3702

815

DrugBank ID: DB00332

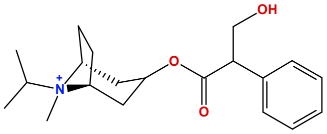

Docking Score: -5.3666

816

DrugBank ID: DB13952

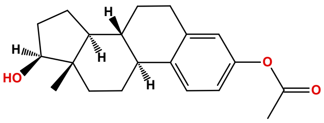

Docking Score: -5.3651

817

DrugBank ID: DB00973

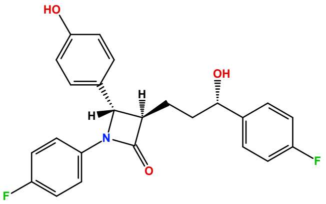

Docking Score: -5.3648

818

DrugBank ID: DB01231

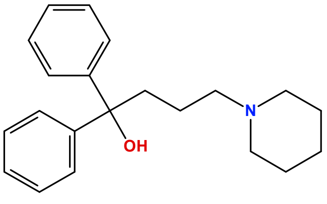

Docking Score: -5.3638

819

DrugBank ID: DB00771

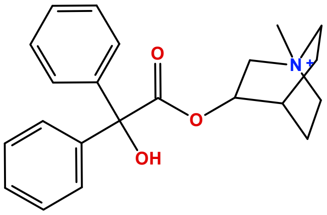

Docking Score: -5.3638

820

DrugBank ID: DB09237

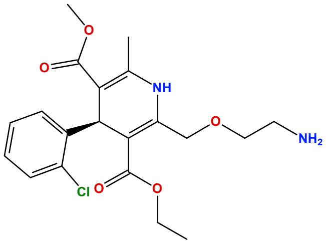

Docking Score: -5.3621

821

DrugBank ID: DB11820

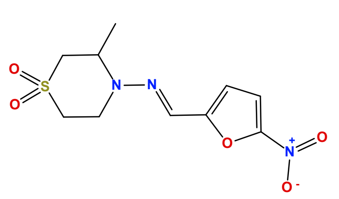

Docking Score: -5.3620

822

DrugBank ID: DB09092

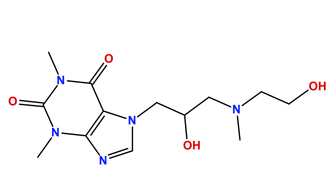

Docking Score: -5.3607

823

DrugBank ID: DB09274

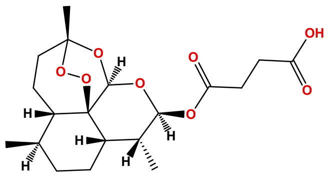

Docking Score: -5.3591

824

DrugBank ID: DB01656

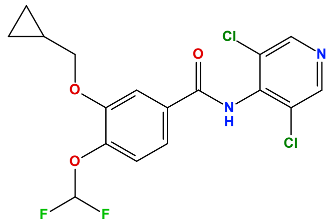

Docking Score: -5.3563

825

DrugBank ID: DB01580

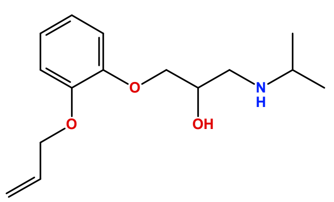

Docking Score: -5.3554

826

DrugBank ID: DB00806

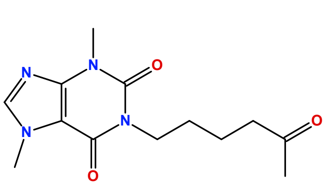

Docking Score: -5.3536

827

DrugBank ID: DB01244

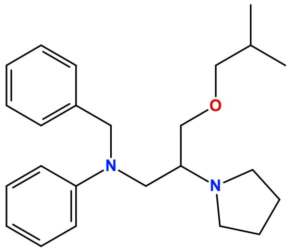

Docking Score: -5.3437

828

DrugBank ID: DB00426

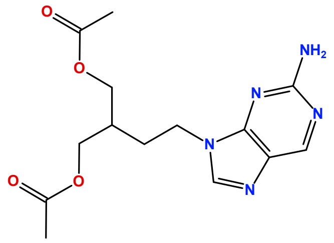

Docking Score: -5.3419

829

DrugBank ID: DB00814

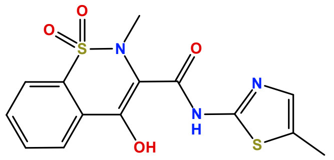

Docking Score: -5.3418

830

DrugBank ID: DB00925

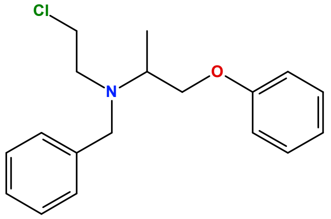

Docking Score: -5.3391

831

DrugBank ID: DB11181

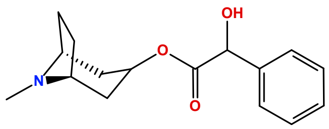

Docking Score: -5.3380

832

DrugBank ID: DB01261

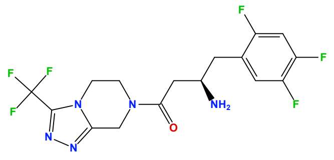

Docking Score: -5.3371

833

DrugBank ID: DB05219

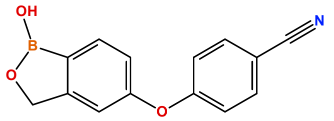

Docking Score: -5.3334

834

DrugBank ID: DB14600

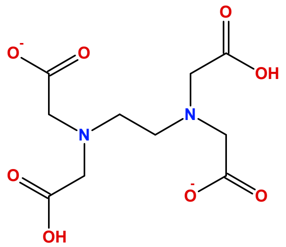

Docking Score: -5.3324

835

DrugBank ID: DB00409

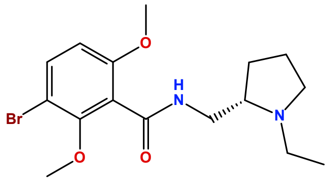

Docking Score: -5.3307

836

DrugBank ID: DB11164

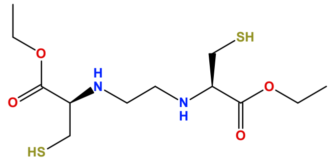

Docking Score: -5.3280

837

DrugBank ID: DB04574

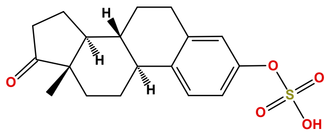

Docking Score: -5.3278

838

DrugBank ID: DB01131

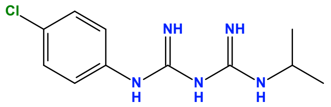

Docking Score: -5.3240

839

DrugBank ID: DB14203

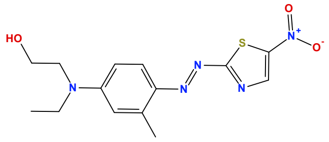

Docking Score: -5.3238

840

DrugBank ID: DB01280

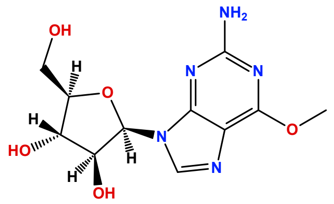

Docking Score: -5.3229

841

DrugBank ID: DB00140

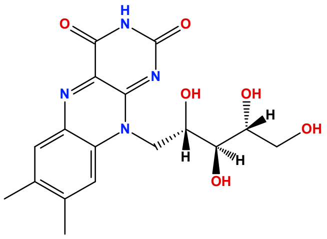

Docking Score: -5.3226

842

DrugBank ID: DB01081

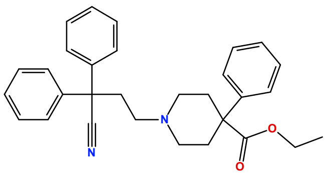

Docking Score: -5.3212

843

DrugBank ID: DB01222

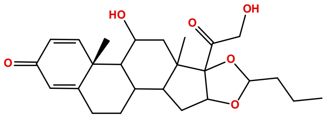

Docking Score: -5.3200

844

DrugBank ID: DB00524

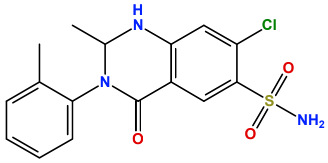

Docking Score: -5.3192

845

DrugBank ID: DB00369

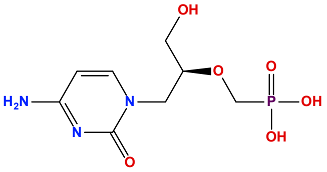

Docking Score: -5.3168

846

DrugBank ID: DB00242

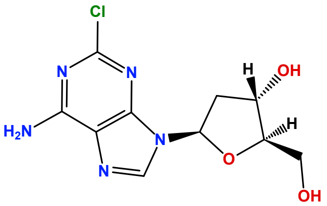

Docking Score: -5.3161

847

DrugBank ID: DB01025

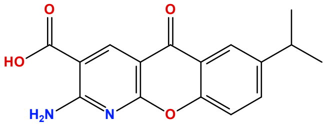

Docking Score: -5.3159

848

DrugBank ID: DB00273

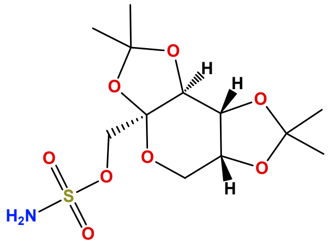

Docking Score: -5.3158

849

DrugBank ID: DB08880

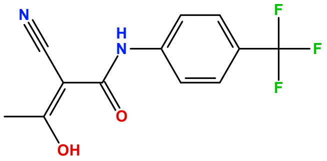

Docking Score: -5.3146

850

DrugBank ID: DB00674

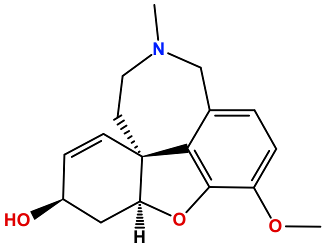

Docking Score: -5.3143

851

DrugBank ID: DB01030

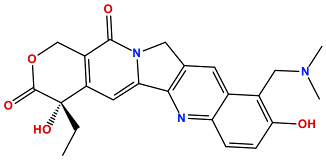

Docking Score: -5.3142

852

DrugBank ID: DB00500

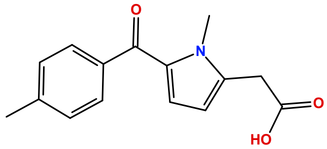

Docking Score: -5.3099

853

DrugBank ID: DB00508

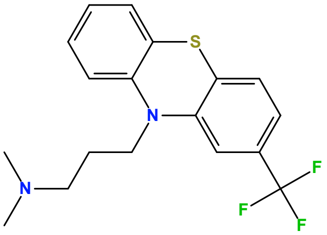

Docking Score: -5.3066

854

DrugBank ID: DB00748

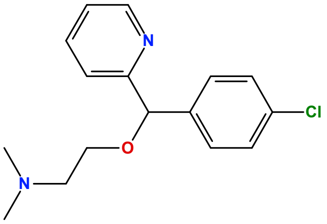

Docking Score: -5.3064

855

DrugBank ID: DB13248

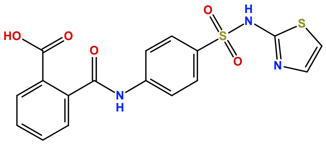

Docking Score: -5.3060

856

DrugBank ID: DB01216

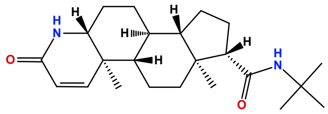

Docking Score: -5.3058

857

DrugBank ID: DB09225

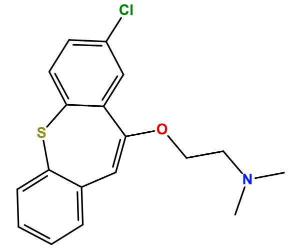

Docking Score: -5.3054

858

DrugBank ID: DB08877

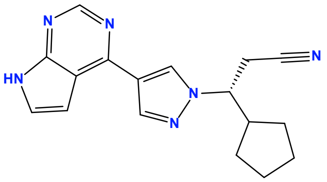

Docking Score: -5.3053

859

DrugBank ID: DB00572

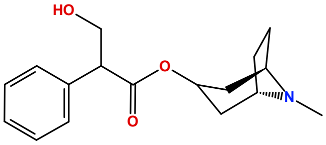

Docking Score: -5.3044

860

DrugBank ID: DB01035

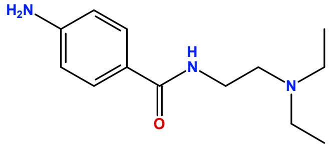

Docking Score: -5.3032

861

DrugBank ID: DB00459

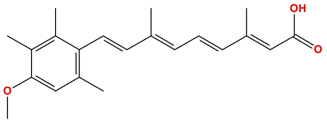

Docking Score: -5.3032

862

DrugBank ID: DB13286

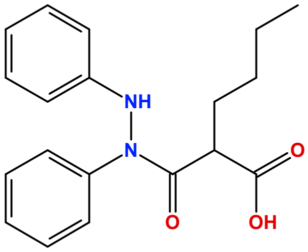

Docking Score: -5.3026

863

DrugBank ID: DB00288

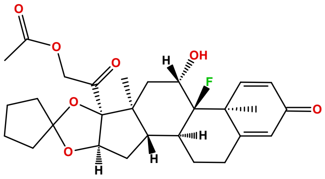

Docking Score: -5.3015

864

DrugBank ID: DB06154

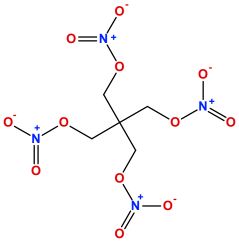

Docking Score: -5.3013

865

DrugBank ID: DB00652

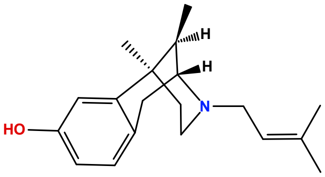

Docking Score: -5.2996

866

DrugBank ID: DB01382

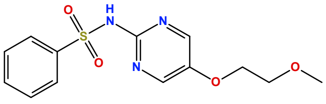

Docking Score: -5.2995

867

DrugBank ID: DB00417

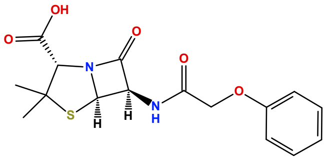

Docking Score: -5.2989

868

DrugBank ID: DB09124

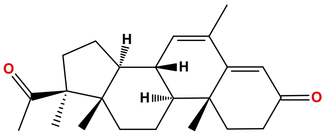

Docking Score: -5.2944

869

DrugBank ID: DB01298

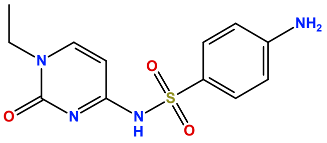

Docking Score: -5.2942

870

DrugBank ID: DB13867

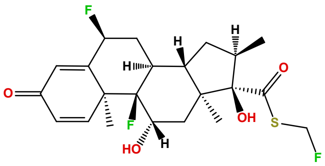

Docking Score: -5.2927

871

DrugBank ID: DB12332

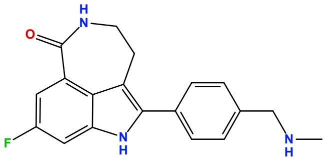

Docking Score: -5.2913

872

DrugBank ID: DB13501

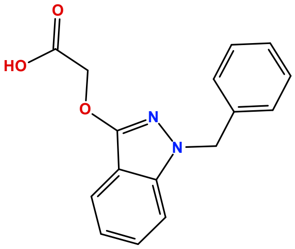

Docking Score: -5.2877

873

DrugBank ID: DB08824

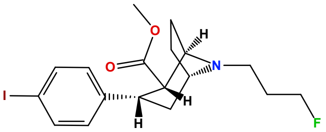

Docking Score: -5.2874

874

DrugBank ID: DB00367

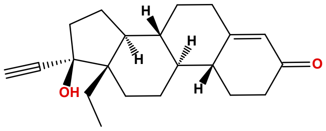

Docking Score: -5.2858

875

DrugBank ID: DB09364

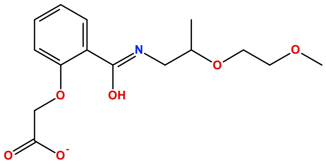

Docking Score: -5.2804

876

DrugBank ID: DB00558

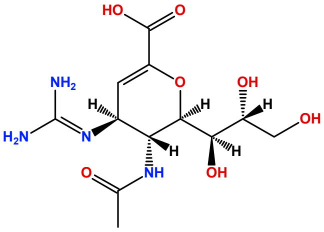

Docking Score: -5.2797

877

DrugBank ID: DB01126

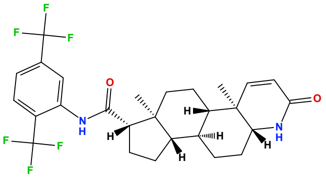

Docking Score: -5.2794

878

DrugBank ID: DB09320

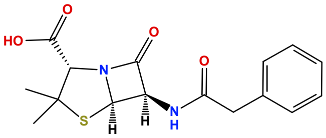

Docking Score: -5.2786

879

DrugBank ID: DB00991

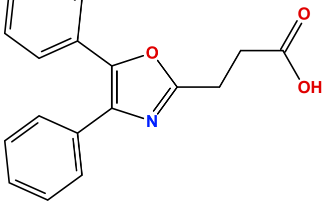

Docking Score: -5.2759

880

DrugBank ID: DB00307

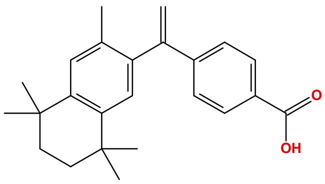

Docking Score: -5.2749

881

DrugBank ID: DB06736

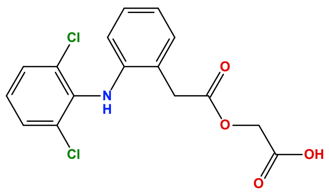

Docking Score: -5.2748

882

DrugBank ID: DB01559

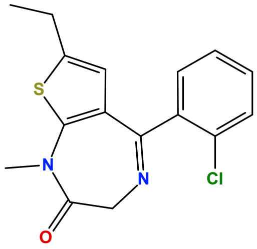

Docking Score: -5.2694

883

DrugBank ID: DB00598

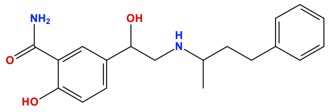

Docking Score: -5.2693

884

DrugBank ID: DB00415

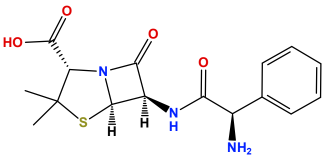

Docking Score: -5.2652

885

DrugBank ID: DB00219

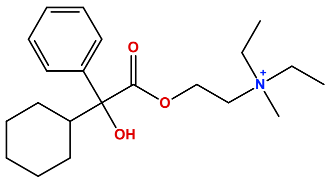

Docking Score: -5.2636

886

DrugBank ID: DB14638

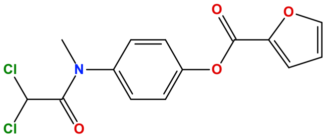

Docking Score: -5.2633

887

DrugBank ID: DB00605

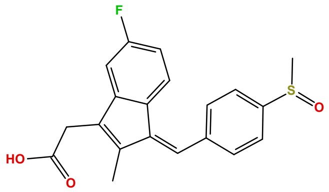

Docking Score: -5.2626

888

DrugBank ID: DB08973

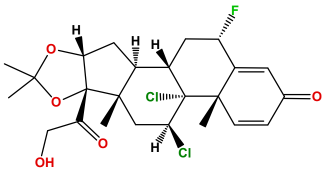

Docking Score: -5.2623

889

DrugBank ID: DB00323

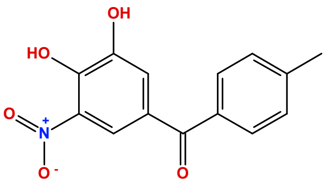

Docking Score: -5.2612

890

DrugBank ID: DB01044

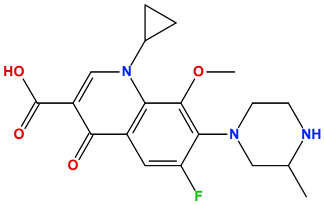

Docking Score: -5.2598

891

DrugBank ID: DB01255

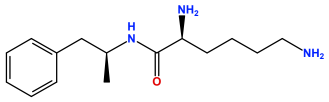

Docking Score: -5.2574

892

DrugBank ID: DB13278

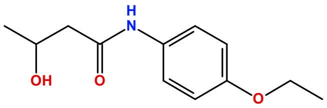

Docking Score: -5.2558

893

DrugBank ID: DB13216

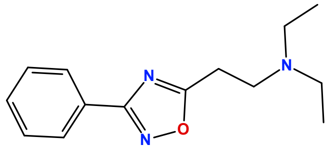

Docking Score: -5.2553

894

DrugBank ID: DB06193

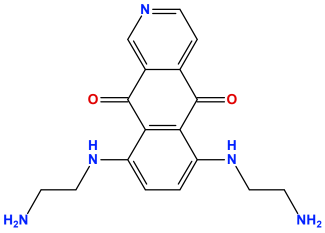

Docking Score: -5.2552

895

DrugBank ID: DB11263

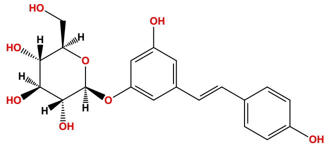

Docking Score: -5.2550

896

DrugBank ID: DB01218

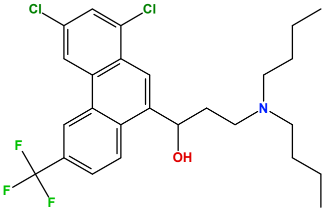

Docking Score: -5.2541

897

DrugBank ID: DB01142

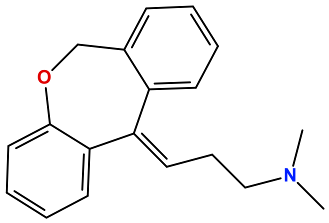

Docking Score: -5.2526

898

DrugBank ID: DB01097

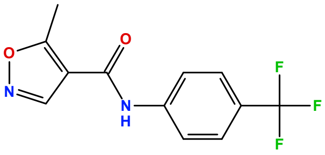

Docking Score: -5.2521

899

DrugBank ID: DB00613

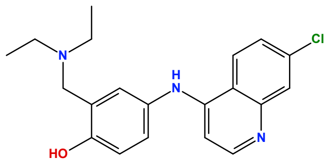

Docking Score: -5.2491

900

DrugBank ID: DB01324

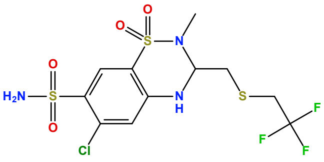

Docking Score: -5.2447

901

DrugBank ID: DB11989

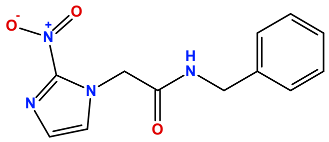

Docking Score: -5.2434

902

DrugBank ID: DB00822

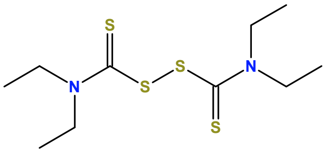

Docking Score: -5.2429

903

DrugBank ID: DB01171

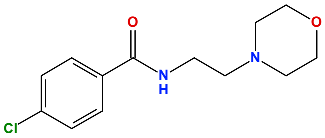

Docking Score: -5.2397

904

DrugBank ID: DB00581

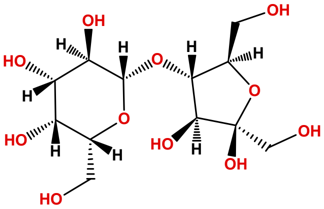

Docking Score: -5.2395

905

DrugBank ID: DB01000

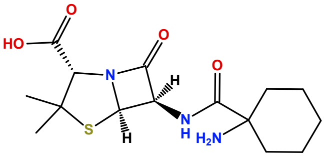

Docking Score: -5.2390

906

DrugBank ID: DB00963

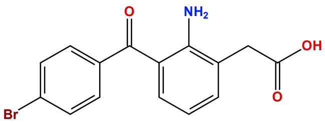

Docking Score: -5.2384

907

DrugBank ID: DB09167

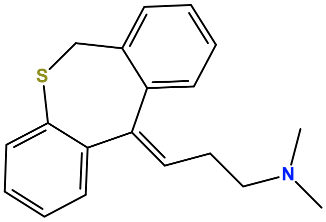

Docking Score: -5.2369

908

DrugBank ID: DB08950

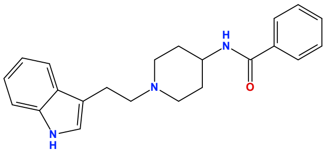

Docking Score: -5.2367

909

DrugBank ID: DB01116

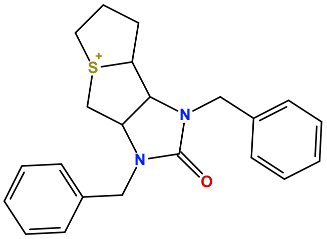

Docking Score: -5.2345

910

DrugBank ID: DB14650

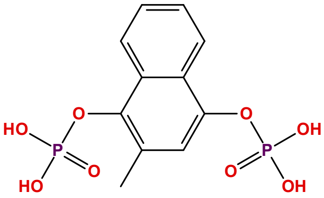

Docking Score: -5.2333

911

DrugBank ID: DB01205

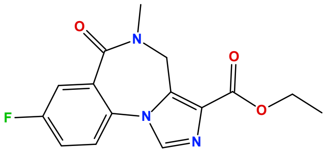

Docking Score: -5.2328

912

DrugBank ID: DB00253

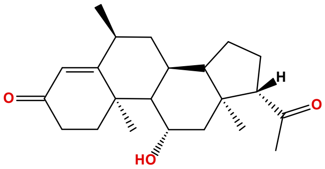

Docking Score: -5.2320

913

DrugBank ID: DB00710

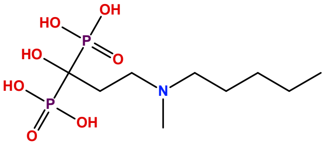

Docking Score: -5.2317

914

DrugBank ID: DB09219

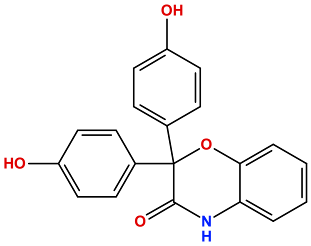

Docking Score: -5.2297

915

DrugBank ID: DB13711

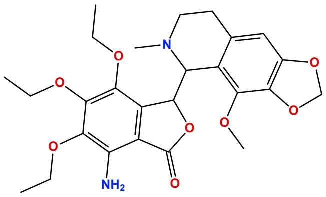

Docking Score: -5.2278

916

DrugBank ID: DB00682

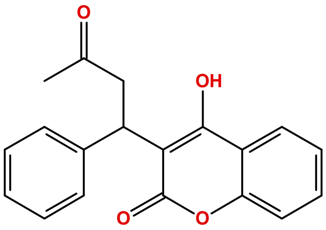

Docking Score: -5.2267

917

DrugBank ID: DB11160

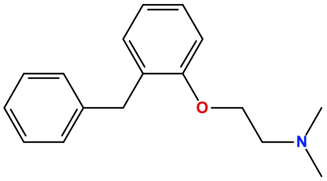

Docking Score: -5.2257

918

DrugBank ID: DB06261

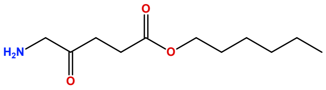

Docking Score: -5.2242

919

DrugBank ID: DB00447

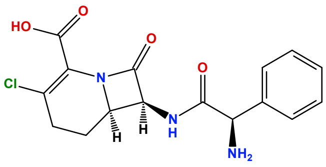

Docking Score: -5.2226

920

DrugBank ID: DB11336

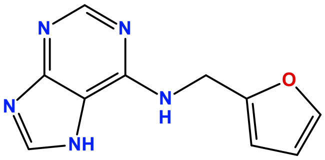

Docking Score: -5.2193

921

DrugBank ID: DB09133

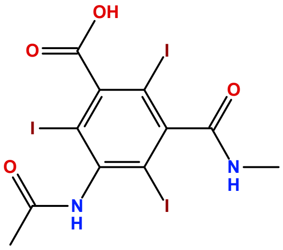

Docking Score: -5.2173

922

DrugBank ID: DB06762

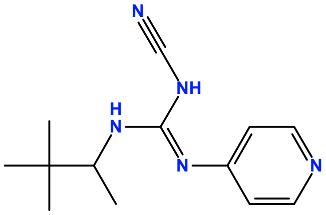

Docking Score: -5.2165

923

DrugBank ID: DB01395

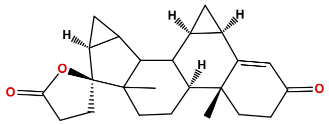

Docking Score: -5.2164

924

DrugBank ID: DB01012

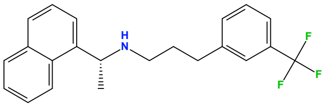

Docking Score: -5.2112

925

DrugBank ID: DB01628

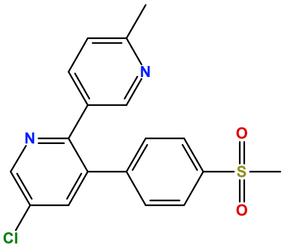

Docking Score: -5.2111

926

DrugBank ID: DB00405

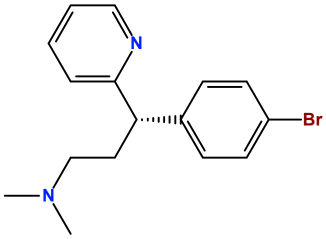

Docking Score: -5.2097

927

DrugBank ID: DB00935

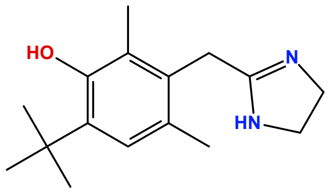

Docking Score: -5.2078

928

DrugBank ID: DB00462

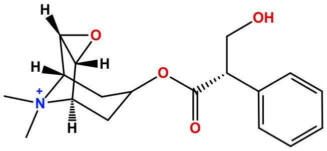

Docking Score: -5.2067

929

DrugBank ID: DB00804

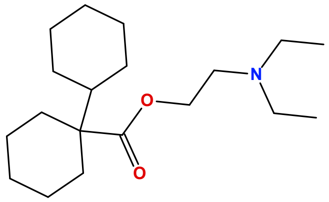

Docking Score: -5.1969

930

DrugBank ID: DB11560

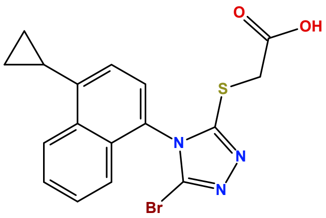

Docking Score: -5.1962

931

DrugBank ID: DB01186

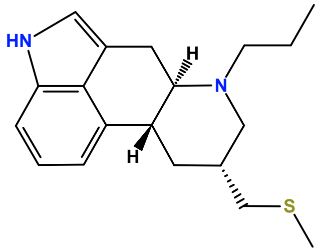

Docking Score: -5.1953

932

DrugBank ID: DB01247

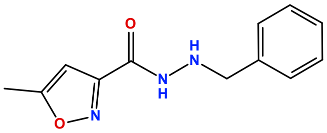

Docking Score: -5.1944

933

DrugBank ID: DB14975

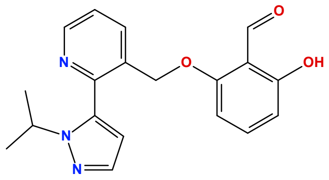

Docking Score: -5.1932

934

DrugBank ID: DB00760

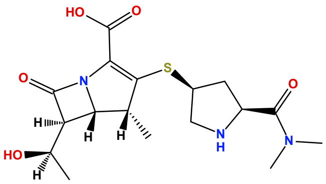

Docking Score: -5.1924

935

DrugBank ID: DB00611

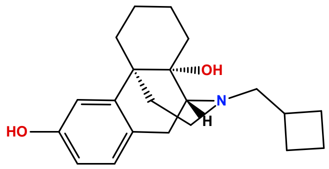

Docking Score: -5.1891

936

DrugBank ID: DB01004

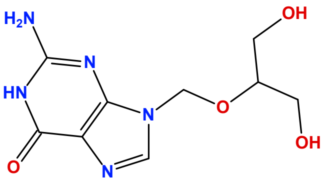

Docking Score: -5.1875

937

DrugBank ID: DB08872

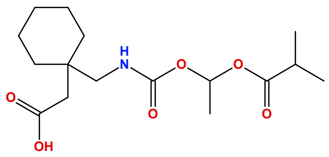

Docking Score: -5.1832

938

DrugBank ID: DB00365

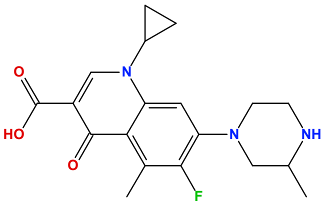

Docking Score: -5.1813

939

DrugBank ID: DB01623

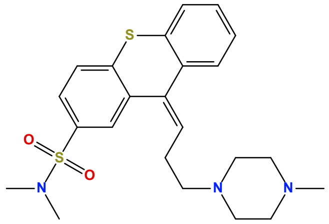

Docking Score: -5.1807

940

DrugBank ID: DB01233

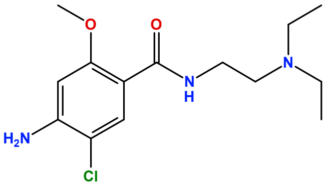

Docking Score: -5.1799

941

DrugBank ID: DB00480

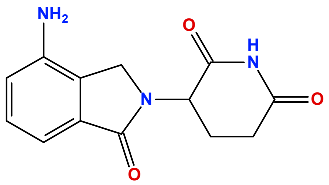

Docking Score: -5.1791

942

DrugBank ID: DB13155

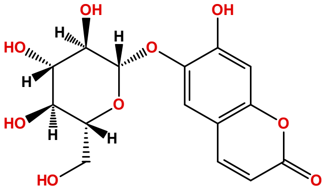

Docking Score: -5.1779

943

DrugBank ID: DB00606

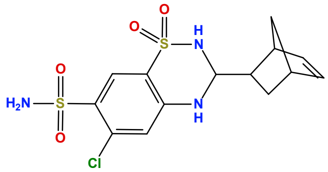

Docking Score: -5.1754

944

DrugBank ID: DB01113

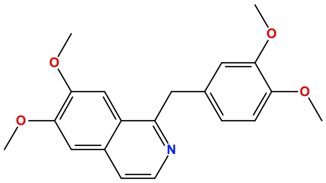

Docking Score: -5.1746

945

DrugBank ID: DB12783

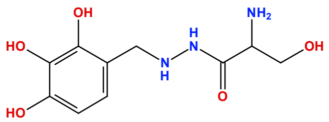

Docking Score: -5.1734

946

DrugBank ID: DB09282

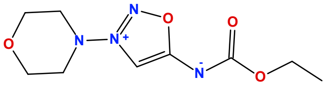

Docking Score: -5.1720

947

DrugBank ID: DB00808

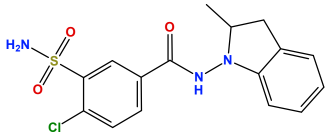

Docking Score: -5.1720

948

DrugBank ID: DB09333

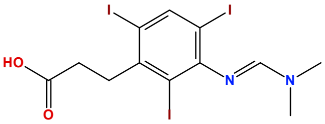

Docking Score: -5.1716

949

DrugBank ID: DB00473

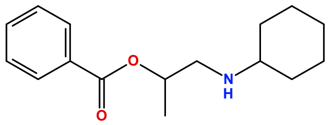

Docking Score: -5.1703

950

DrugBank ID: DB09009

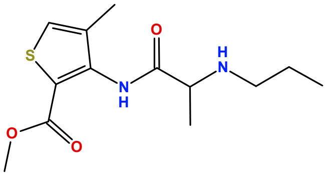

Docking Score: -5.1694

951

DrugBank ID: DB00596

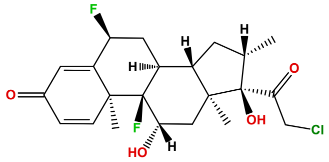

Docking Score: -5.1623

952

DrugBank ID: DB11201

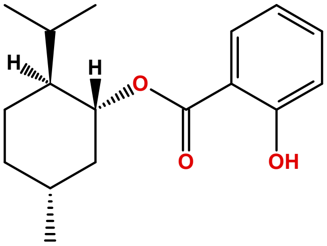

Docking Score: -5.1601

953

DrugBank ID: DB00552

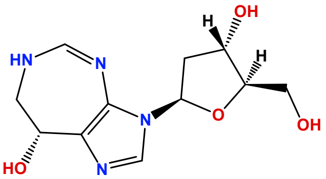

Docking Score: -5.1585

954

DrugBank ID: DB01607

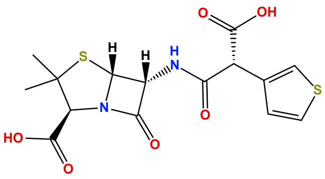

Docking Score: -5.1565

955

DrugBank ID: DB13267

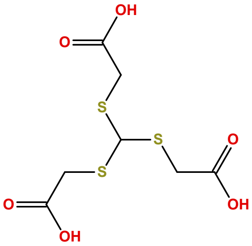

Docking Score: -5.1563

956

DrugBank ID: DB00486

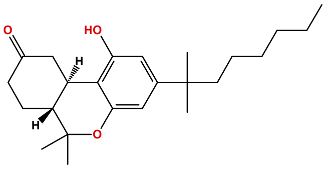

Docking Score: -5.1555

957

DrugBank ID: DB01079

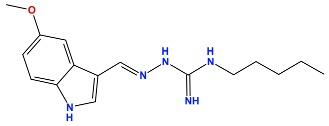

Docking Score: -5.1554

958

DrugBank ID: DB00363

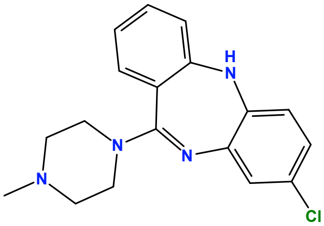

Docking Score: -5.1519

959

DrugBank ID: DB01176

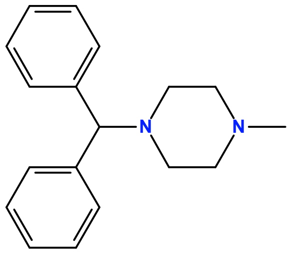

Docking Score: -5.1510

960

DrugBank ID: DB00695

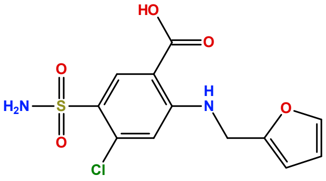

Docking Score: -5.1492

961

DrugBank ID: DB01346

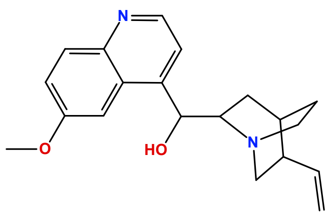

Docking Score: -5.1462

962

DrugBank ID: DB00240

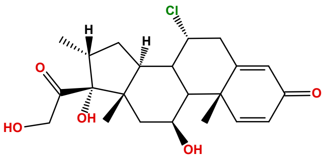

Docking Score: -5.1451

963

DrugBank ID: DB01115

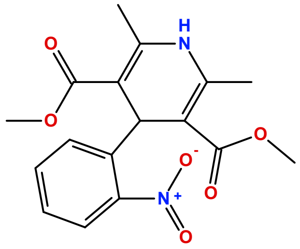

Docking Score: -5.1447

964

DrugBank ID: DB00643

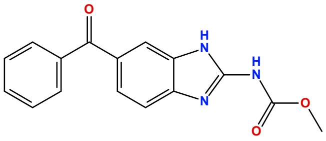

Docking Score: -5.1424

965

DrugBank ID: DB00292

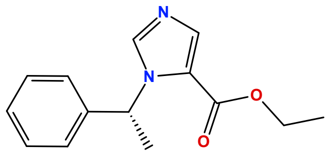

Docking Score: -5.1410

966

DrugBank ID: DB01036

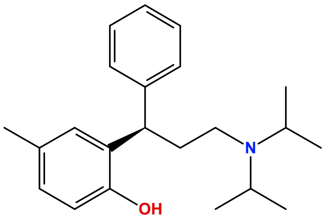

Docking Score: -5.1409

967

DrugBank ID: DB04038

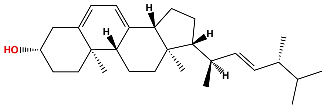

Docking Score: -5.1402

968

DrugBank ID: DB00210

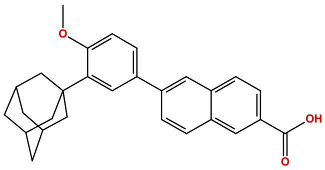

Docking Score: -5.1378

969

DrugBank ID: DB01002

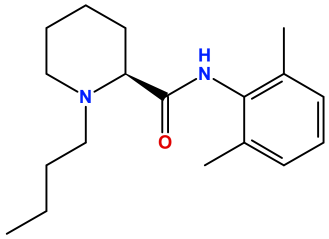

Docking Score: -5.1364

970

DrugBank ID: DB06766

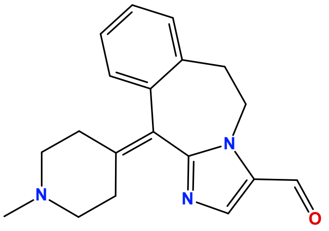

Docking Score: -5.1354

971

DrugBank ID: DB04825

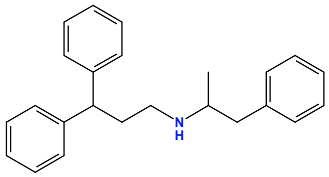

Docking Score: -5.1348

972

DrugBank ID: DB00451

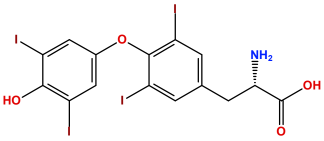

Docking Score: -5.1329

973

DrugBank ID: DB00461

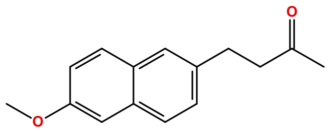

Docking Score: -5.1325

974

DrugBank ID: DB08981

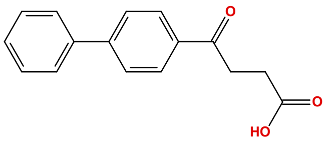

Docking Score: -5.1315

975

DrugBank ID: DB01625

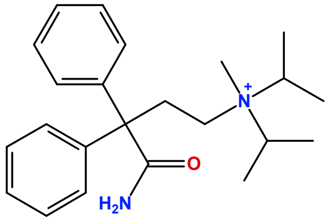

Docking Score: -5.1279

976

DrugBank ID: DB06821

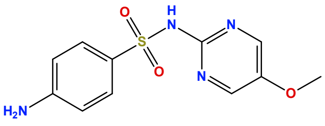

Docking Score: -5.1269

977

DrugBank ID: DB01064

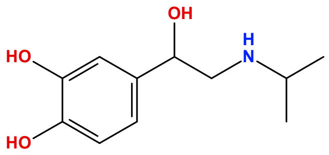

Docking Score: -5.1259

978

DrugBank ID: DB00755

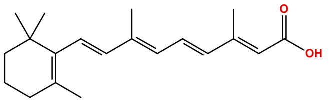

Docking Score: -5.1256

979

DrugBank ID: DB00330

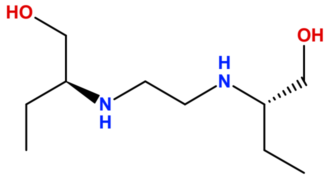

Docking Score: -5.1238

980

DrugBank ID: DB01208

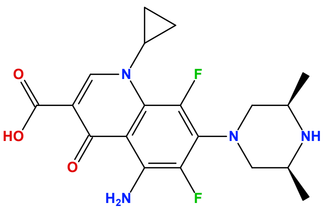

Docking Score: -5.1235

981

DrugBank ID: DB01613

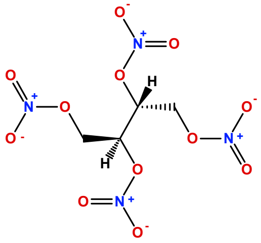

Docking Score: -5.1234

982

DrugBank ID: DB14655

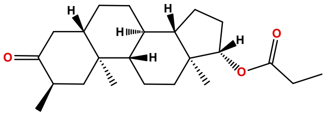

Docking Score: -5.1228

983

DrugBank ID: DB12343

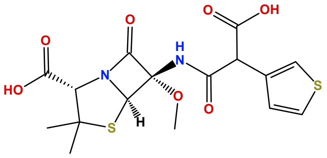

Docking Score: -5.1220

984

DrugBank ID: DB01090

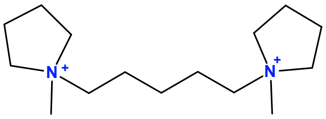

Docking Score: -5.1200

985

DrugBank ID: DB00449

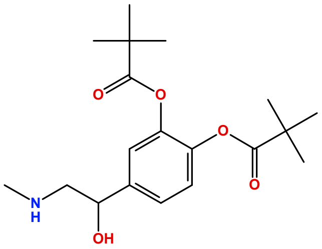

Docking Score: -5.1189

986

DrugBank ID: DB00986

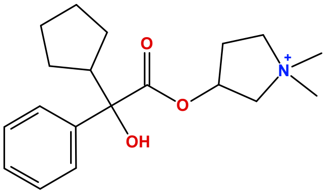

Docking Score: -5.1188

987

DrugBank ID: DB09085

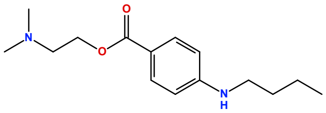

Docking Score: -5.1186

988

DrugBank ID: DB00784

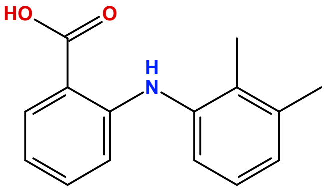

Docking Score: -5.1167

989

DrugBank ID: DB00414

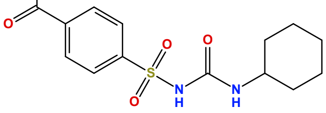

Docking Score: -5.1146

990

DrugBank ID: DB06694

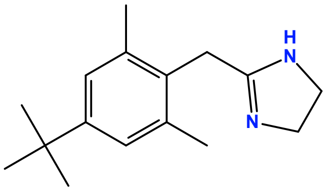

Docking Score: -5.1143

991

DrugBank ID: DB00608

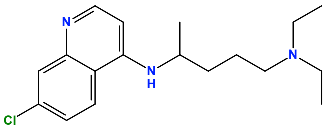

Docking Score: -5.1116

992

DrugBank ID: DB11062

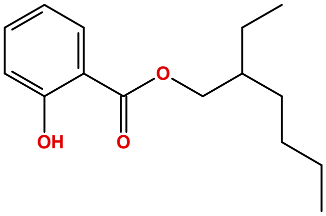

Docking Score: -5.1105

993

DrugBank ID: DB12362

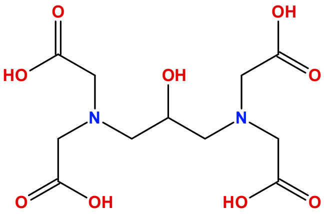

Docking Score: -5.1100

994

DrugBank ID: DB06718

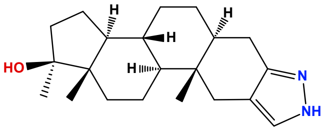

Docking Score: -5.1086

995

DrugBank ID: DB08799

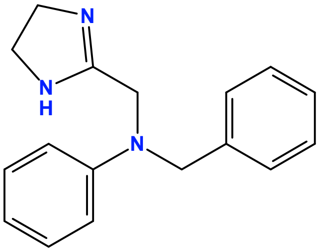

Docking Score: -5.1084

996

DrugBank ID: DB11279

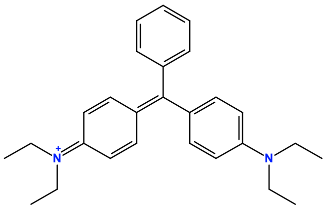

Docking Score: -5.1076

997

DrugBank ID: DB06152

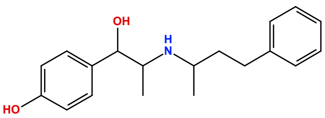

Docking Score: -5.1068

998

DrugBank ID: DB00361

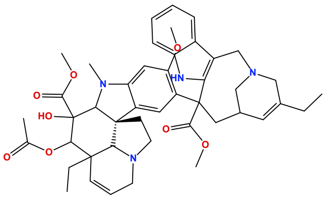

Docking Score: -5.1052

999

DrugBank ID: DB06803

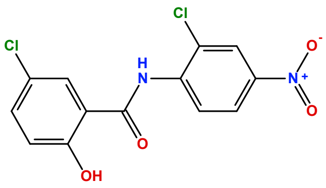

Docking Score: -5.1038

1000

DrugBank ID: DB06412

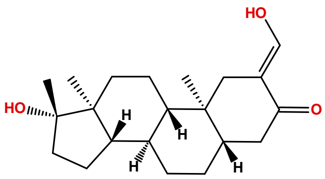

Docking Score: -5.1037
