# Supplementary material for: Purification and Inhibitor Screening of the Full-Length SARS-CoV-2 Nucleocapsid Protein
Source: Molecules. 2025 Jun 20;30(13):2679. doi: 10.3390/molecules30132679 (PMC12251317; doi:10.3390/molecules30132679)
Supplement: Supplementary file 1 [file molecules-30-02679-s001.zip › molecules-3608758-supplementary/Figure S1.pdf]

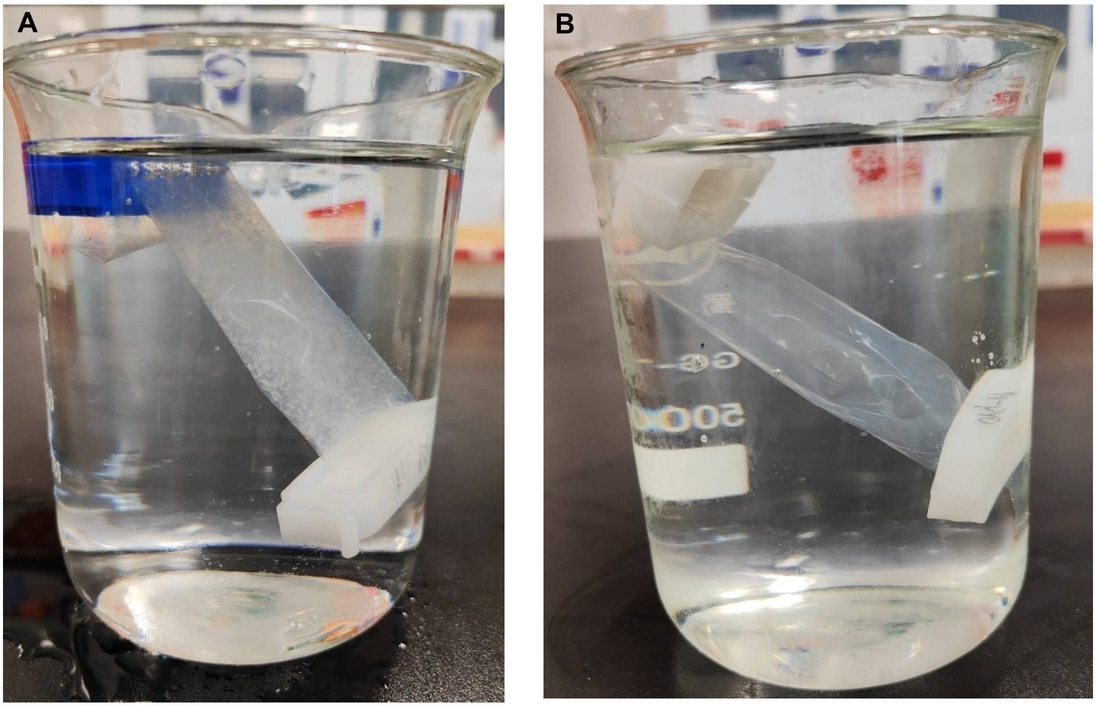

Figure S1. N protein dialysis results of different purification processes. A The N protein precipitated in dialysis step (N protein was not treated with PEI and ammonium sulfate). B The N protein treated with PEI and ammonium sulfate did not precipitate in dialysis (Figure S 1B).
