# Supplementary material for: Purification and Inhibitor Screening of the Full-Length SARS-CoV-2 Nucleocapsid Protein
Source: Molecules. 2025 Jun 20;30(13):2679. doi: 10.3390/molecules30132679 (PMC12251317; doi:10.3390/molecules30132679)
Supplement: Supplementary file 1 [file molecules-30-02679-s001.zip › molecules-3608758-supplementary/Figure S2.pdf]

1

CAS: 303-97-9

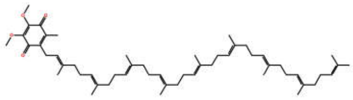

Docking Score: -9.5162

2

CAS: 4235-95-4

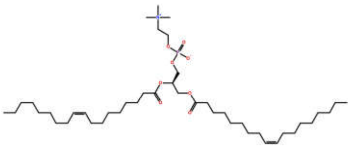

Docking Score: -9.1942

3

CAS: 145937-22-0

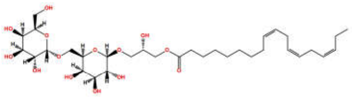

Docking Score: -9.0490

4

CAS: 13190-97-1

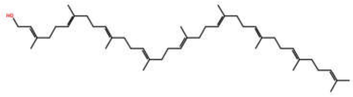

Docking Score: -9.0222

5

CAS: 111187-15-6

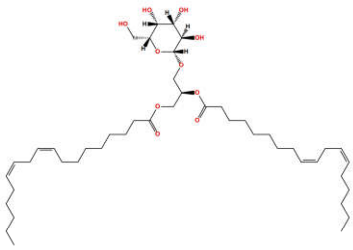

Docking Score: -8.7693

6

CAS: 154801-30-6

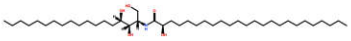

Docking Score: -8.5535

7

CAS: 164022-75-7

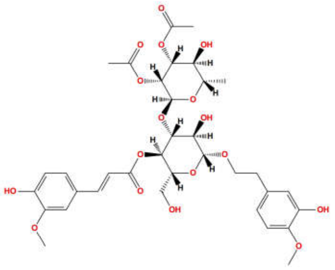

Docking Score: -8.5428

8

CAS: 1069-79-0

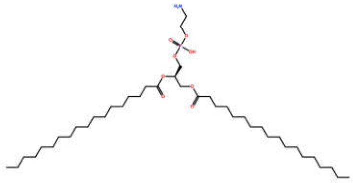

Docking Score: -8.5423

9

CAS: 122021-74-3

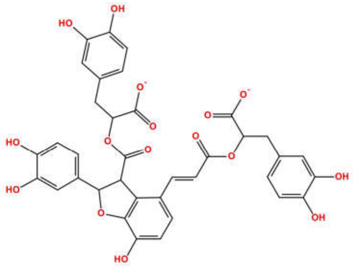

Docking Score: -8.5202

10

CAS: 555-45-3

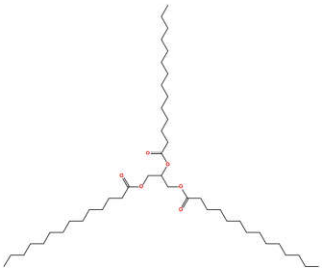

Docking Score: -8.4654

11

CAS: 116355-83-0

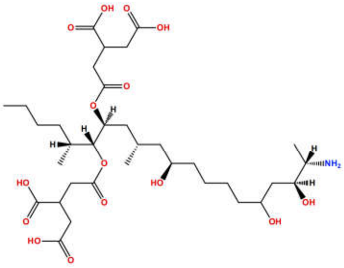

Docking Score: -8.3843

12

CAS: 114297-20-0

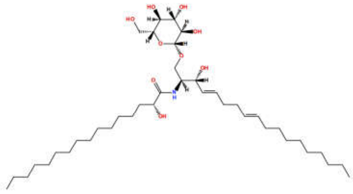

Docking Score: -8.3642

13

CAS: 125353-53-9

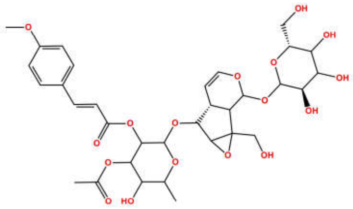

Docking Score: -8.2556

14

CAS: 604-33-1

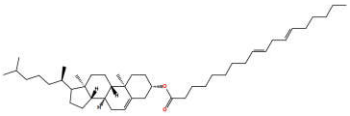

Docking Score: -8.2426

15

CAS: 187393-00-6

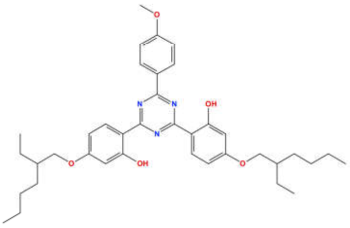

Docking Score: -8.2355

16

CAS: 127422-61-1

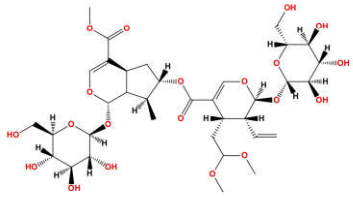

Docking Score: -8.1946

17

CAS: 606-68-8

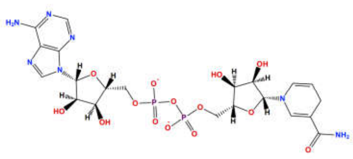

Docking Score: -8.1786

18

CAS: 111035-65-5

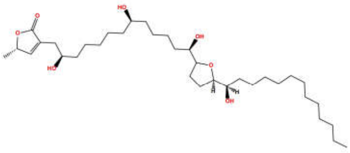

Docking Score: -8.1700

19

CAS: 156791-81-0

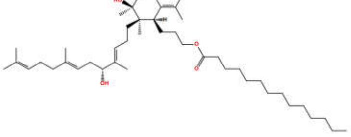

Docking Score: -8.1596

20

CAS: 301-19-9

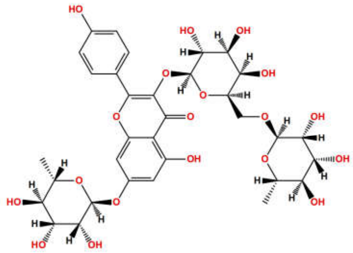

Docking Score: -8.1390
