# Supplementary material for: Purification and Inhibitor Screening of the Full-Length SARS-CoV-2 Nucleocapsid Protein
Source: Molecules. 2025 Jun 20;30(13):2679. doi: 10.3390/molecules30132679 (PMC12251317; doi:10.3390/molecules30132679)
Supplement: Supplementary file 1 [file molecules-30-02679-s001.zip › molecules-3608758-supplementary/Figure S3.pdf]

1

DrugBank ID: DB14099

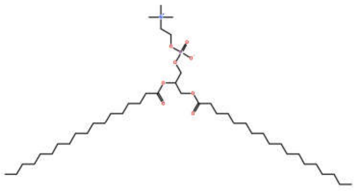

Docking Score: -9.3441

2

DrugBank ID: DB11284

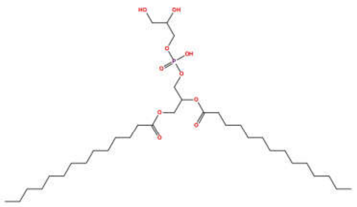

Docking Score: -8.8725

3

DrugBank ID: DB06811

Docking Score: -8.6384

4

DrugBank ID: DB09065

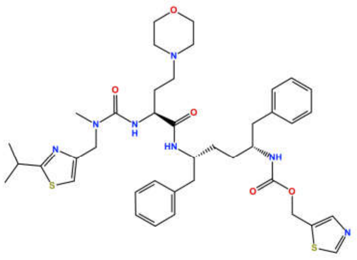

Docking Score: -8.4982

5

DrugBank ID: DB11183

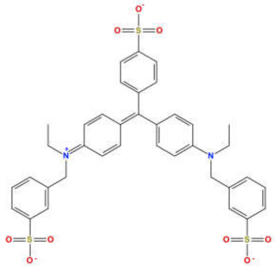

Docking Score: -8.3788

6

DrugBank ID: DB06804

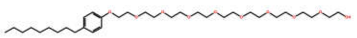

Docking Score: -8.3608

7

DrugBank ID: DB11660

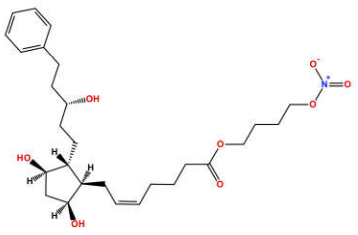

Docking Score: -8.2228

8

DrugBank ID: DB14879

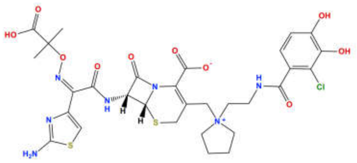

Docking Score: -8.0873

9

DrugBank ID: DB00385

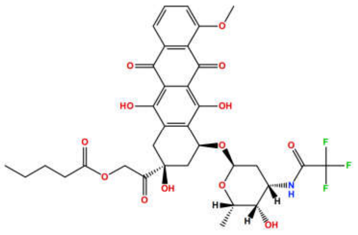

Docking Score: -8.0511

10

DrugBank ID: DB08909

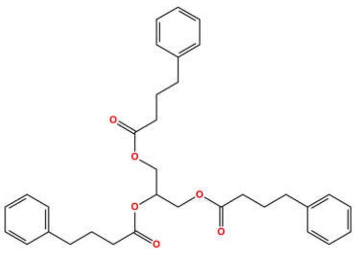

Docking Score: -7.9994

11

DrugBank ID: DB14185

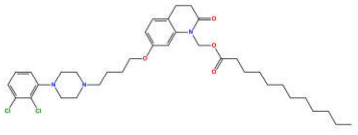

Docking Score: -7.9676

12

DrugBank ID: DB01167

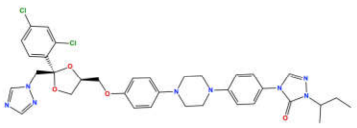

Docking Score: -7.9113

13

DrugBank ID: DB00944

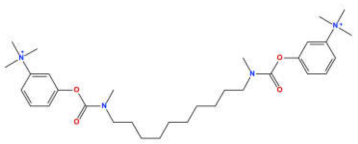

Docking Score: -7.9059

14

DrugBank ID: DB00390

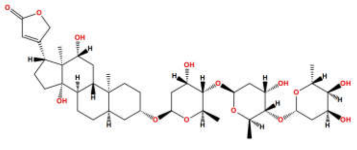

Docking Score: -7.8543

15

DrugBank ID: DB00430

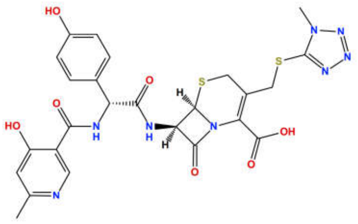

Docking Score: -7.8461

16

DrugBank ID: DB11206

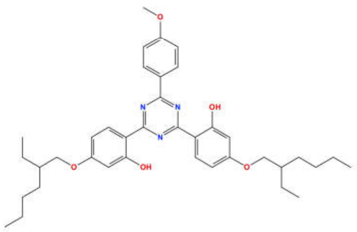

Docking Score: -7.8376

17

DrugBank ID: DB12500

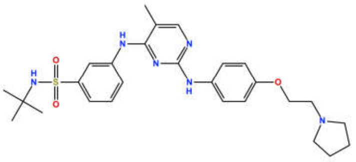

Docking Score: -7.8245

18

DrugBank ID: DB01232

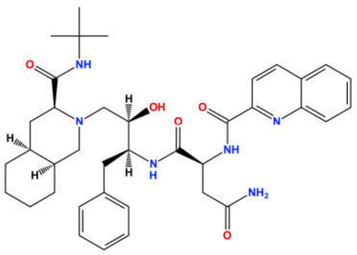

Docking Score: -7.7796

19

DrugBank ID: DB11986

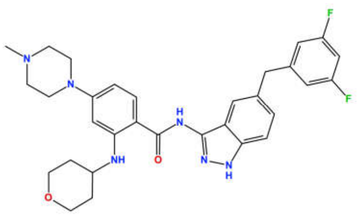

Docking Score: -7.7719

20

DrugBank ID: DB06636

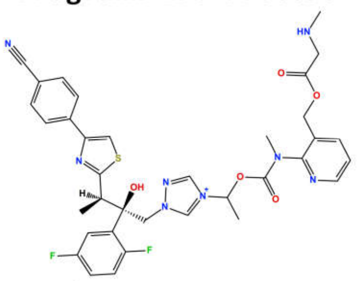

Docking Score: -7.7539
